# Revision of *Poa* L. (Poaceae, Pooideae, Poeae, Poinae) in Mexico: new records, re-evaluation of *P. ruprechtii*, and two new species, *P. palmeri* and *P. wendtii*

**DOI:** 10.3897/phytokeys.15.3084

**Published:** 2012-08-06

**Authors:** Robert J. Soreng, Paul M. Peterson

**Affiliations:** 1 Department of Botany, National Museum of Natural History, Smithsonian Institution Washington, DC, 20013-7012, USA

**Keywords:** Mexico, *Poa*, Poaceae, taxonomy

## Abstract

A revision and key to the 23 species and eight subspecies of *Poa* (including *Dissanthelium*) known to occur in Mexico is provided. All voucher specimens seen are cited for accepted taxa, except *Poa annua* for which one voucher per state is provided. Taxa not previously known from, or poorly understood in, Mexico are discussed.*Poa palmeri* **sp. nov.** is endemic to forested slopes of the Sierra Madre Oriental, and we distinguished it from *Poa ruprechtii* s.s., a species of central Mexico that is here emended to include *Poa sharpii* (syn. nov.). *Poa wendtii* **sp. nov.** is described from the Sierra Santa Rosa in northern Coahuila. *Poa tacanae* is placed in synonymy in *Poa seleri*. *Poa gymnantha* and *Poa occidentalis* are newly reported for Mexico, and material historically identified as *Poa villaroelii* areplaced in *Poa chamaeclinos*.The genus *Dissanthelium* is considered to belong within *Poa*, and the Mexican taxa, *Dissanthelium calycina* subsp. *mathewsii* and *Dissanthelium californicum*, are treated as *Poa calycina* var.* mathewsii* and *Poa thomasii*, respectively. *Poa* subsect. *Papillopoa* **subsect. nov.** is erected for *Poa mulleri*. Lectotypes are designated for *Poa conglomerata* and *Poa seleri*.

## Abstract (es)

Se realizó la revisión del género *Poa* (incluyendo *Dissanthelium*) de México, se proporciona una clave para las 23 especies y ocho subspecies encontradas. Se citan todos los ejemplares revisados para los taxa aceptados, excepto los de *Poa annua* para el cual se proporciona un ejemplar por cada estado. Los taxa no conocidos previamente, o mal entendidos de México se discuten. *Poa palmeri* **sp. nov.** es endémica de las cuestas boscosas de la Sierra Madre Oriental, la distinguimos de *Poa ruprechtii* s.s., una especie del México central que aquí se enmienda para ser incluida, esta ultima en *Poa sharpii* (sin. nov.). *Poa wendtii* **sp. nov.** se describe de la Sierra Santa Rosa del norte de Coahuila. *Poa tacanae* se coloca en sinonimia de *Poa seleri*. *Poa gymnantha* y *Poa occidentalis* se reportan nuevamente para México, y el material identificado históricamente como *Poa villaroelii* se coloca en *Poa chamaeclinos*. El género *Dissanthelium* se considera formar parte de *Poa*, y los taxa mexicanos, *Dissanthelium calycina* subsp. *mathewsii* y *Dissanthelium californicum*, se tratan como *Poa calycina* var. *mathewsii* y *Poa thomasii*, respectivamente. *Poa* subsect. *Papillopoa* **subsect. nov.** se erige para *Poa mulleri*. Se designan lectotipos para *Poa conglomerata* y *Poa seleri*.

## Introduction

Accounts of the genus *Poa* L. in Mexico are mostly the result of new species and summary treatments of all grasses in the country, and most are essentially alfa-taxonomic in nature. These started with J.F. Ruprecht’s list of names of grasses collected in Mexico by H. Galeotti (Galeotti 1842), but effectively began with Peyritsch (1859) who named two new species. Hemsley’s (1882) account, taken from a draft of Fournier’s (1886) treatment published four years later, included six species, one of which, *Poa ciliaris* L. is equal to *Eragrostis ciliaris* (L.) R.Br. Beal (1896) accounted for four species and Hitchcock (1913) reported 11 species of which five were new. Swallen (1940, 1950) described three more species of *Poa* from Mexico (and two from adjacent Guatemala), and Beetle (1977) listed 21 species. Manrique de Skendzic (1999) accounted for 12 species with a key to the genus and descriptions, leaving several formerly widely reported taxa unaccounted for. Treatments of *Poa* in modern checklists covering Mexico (Espejo Serna et al. 2000; Dávila Aranda et al. 2006) recognized 18 and 16 species, respectively. In broader geographical surveys, Soreng et al. (2003a) and Clayton et al. (2006) accepted 16 species in Mexico. Pohl and Davidse (1994) account for five species reaching the Mexican states treated in the Flora Mesoamericana. Soreng (2001) published one new species and discussed others from Baja California, and Peterson et al. (2006) added another species from the Sierra Madre Occidental. Peterson et al. (2006) provided a provisional key to 11 spp. of *Poa* in northern Mexico (excluding Baja California). In that paper we concluded that *Poa ruprechtii* sensu auct. was heterogeneous. However, until we could examine the type we could not be sure of the correct application. Here we restrict the historically broad application of *Poa ruprechtii* Peyr., and treat the material of *Poa ruprechtii* sensu auct. of northeastern Mexico as a new species. The genus *Dissanthelium* Trin. has recently been submerged into *Poa* (Refulio-Rodríguez et al. 2012), and the two Mexican species historically treated in that genus are here included in *Poa*. While examining specimens from northern Coahuila another new species was detected. Due to new discoveries and changes in identification and synonymy, we currently accept 23 species and eight subspecies of *Poa*, and provide a key to all the Mexican species.

## Materials and methods

Specimens of *Poa* of Mexico were recently loaned to US from ISC, MEXU, MO, TAES, TEX (and LL). Other critical materials seen by RJS over the years, mainly while a graduate or postgraduate student at New Mexico State University (1978−1987), are from ARIZ, CAS, DS, GH, LE, LL, NMC, MSC, NY, RSA, SD, TAES, TEX, US, W, WIS, WYAC. During this period RJS contributed to a treatment of *Poa* for A Flora of the Chihuahuan Desert (Henrickson and Johnston, unpubl. manuscript). Synonymy is mainly limited to names of taxa described from Latin America. Additional synonyms accepted by us can be found in the Catalogue of New World Grasses, vol. IV (Soreng et al. 2003a, b) and on the Catalogue of New World Grasses web site (http://www.tropicos.org/Project/CNWG) that is continually updated within Tropicos (http://www.tropicos.org). In the descriptions and key all measurements were taken from the lowest one or two florets in the best-developed spikelets, and sheath openness and ligule length is determined on the upper culm leaf (or the one below that for ligules if the upper blade is absent or rudimentary). When counting culm nodes it is best to start counting 1 cm above the base. Blade width is measured from margin to margin on a flattened blade. Spikelet and bract shapes are based on lateral view. Glabrous refers to without pubescence. Smooth indicates no prickle-hairs with broad bases and/or hooked or pointed apices (i.e. pubescence can occur on a smooth surface, and a scabrous surface can be glabrous). Excluded species and an infrageneric classification of the accepted species of *Poa* in Mexico are presented at the end.

## Taxonomic treatment

### Poa

L., Sp. Pl. 1: 67–70. 1753.

http://species-id.net/wiki/Poa

Paneion Lunell, Amer. Midl. Naturalist 4: 221. 1915; Panicularia Heist. ex Fabr., Enum. 207. 1759; Poagris Raf., Fl. Tellur. 1: 18. 1837. Lectotype: Poa pratensis, designated by Nash 1913: 252.
Anthochloa Nees & Meyen, Reise Erde 2: 14. 1834. Type: Anthochloa lepidula Nees & Meyen. [≡ Poa lepidula (Nees & Meyen.) Soreng & L.J.Gillespie].
Aphanelytrum (Hack.) Hack., Oesterr. Bot. Z. 52: 12. 1902.; Brachyelytrum subgen. Aphanelytrum Hack., Nat. Pflanzenfam. 1: 42. 1897. Type: Anthochloa procumbens (Hack.) Hack.
Austrofestuca (Tzvelev) E.B.Alexeev, Bjull. Moskovsk. Obač. Isp. Prir., Otd. Biol. 81(5): 55. 1976; Festuca subgen. Austrofestuca Tzvelev, Bot. Žurn. (Moscow & Leningrad) 56(9): 1257. 1971. Type: Anthochloa littoralis (Labill.) E.B.Alexeev [≡ Poa billarierei St.-Yves].
Dasypoa Pilg., Bot. Jahrb. Syst. 25(5): 716. 1898. Type: Dasypoa tenuis Pilg. [= Poa scaberula Hook.f.].
Dissanthelium Trin., Linnaea 10(3): 305. 1836. Type: Dissanthelium supinum Trin. [= Poa calycina (J. Presl) Kunth].
Eremopoa Roshev., Fl. URSS 2: 429, 756. 1934. Type: Eremopoa persica (Trin.) Roshev. [≡ Poa persica Trin.].
Graminastrum E.H.L.Krause, Beih. Bot. Centralbl. 32(2): 348. 1914. Type: Graminastrum macusaniense E.H.L.Krause [≡ Poa macusaniense (E.H.L.Krause) Refulio].
Neuropoa Clayton, Kew Bull. 40(4): 728. 1985. Type: Neuropoa fax (Willis & Court) Clayton [≡ Poa fax Willis & Court].
Ochlopoa (Asch. & Graebn.) H.Scholz, Ber. Inst. Lanschafts- Pflanzenokologie Univ. Hohenheim Beih. 16: 58. 2003.; Poa sect. Ochlopoa Asch. & Graebn., Syn. Mitteleur. Fl. 2: 387. 1900. Type: Ochlopoa annua (L.) H.Scholz [≡ Poa annua L.].
Oreopoa Gand., Fl. Eur. 26: 186. 1891. nom. nud., based on: Poa alpina L.
Parodiochloa C.E.Hubb., Bull. Brit. Mus. (Nat. Hist.), Bot. 8: 395. 1981. Type: Parodiochloa flabellata (Lam.) C.E.Hubb. [≡ Poa flabellata (Lam.) Raspail].
Phalaridium Nees & Meyen, Gramineae 29. 1841. Type: Phalaridium peruvianum Nees & Meyen [≡ Poa serpaiana Refulio].
Stenochloa Nutt., Proc. Acad. Nat. Sci. Philadelphia 4: 25. 1848. Type: Stenochloa californica Nutt. [≡ Poa thomasii Refulio].
Tovarochloa T.D.Macfarl. & But, Brittonia 34(4): 478. 1982. Type: Tovarochloa peruviana T.D. Macfarl. & But [≡ Poa apiculata Refulio].
Tzvelevia E.B.Alexeev, Bjull. Moskovsk. Obač. Isp. Prir., Otd. Biol. 90(5): 103. 1985. Type: Tzvelevia kerguelensis (Hook.f.) E.B.Alexeev [≡ Poa kerguelensis (Hook.f.) Hook.f.].

#### Description.

The following combination of characteristics distinguish *Poa* from other grass genera in Mexico, and in most of the world. The plants are annual or perennial, and rarely exceed 1 m in culm length. The culms are simple, hollow, and unbranched above the base. Plants sometimes have rhizomes, but only infrequently are weakly stoloniferous. The leaves are unusual in that the upper culm leaf sheath margins are fused over 5 to 100%; the blades are abaxially keeled and typically have only two channels on the adaxial surface (sometimes obscure), one on either side of the midvein, that are lined with thin-walled bulliform cells that collapse to fold the blade in dry conditions; and the blade apex is typically prow-shaped to some degree (in these features they are like *Glyceria*). The vegetative structures lack long pilose hairs and microhairs. Like all Pooideae, the blades lack Kranz anatomy. Ligules are scareous (white) to hyaline, without apical or lateral cilia (sometimes minutely ciliolate). The inflorescences are simple, paniculate, and terminal. *Poa* spikelets are unspecialized, usually between 2.5 and 8 mm long, normally with 2 and 5 florets (sometimes 1 in *Poa gymnantha*), and disarticulate above the glumes and between the florets. The spikelets and florets are laterally compressed and lack awns. The florets are all alike, but are reduced upward and the uppermost is often rudimentary. The glumes are persistent, typically the lower one is 1 or 3-veined; the upper 3-veined, slightly narrower and thinner than the lemmas, generally slightly shorter than the lower floret (longer in *Poa thomasii* and *Poa calycina*; the lower glume can be quite short in *Poa seleri*), and are often somewhat unequal. The rachilla is terete, usually at least 0.4 mm long between florets and is prolonged beyond the uppermost, well developed floret (except in *Poa calycina* where the internode is 0.2–0.3 mm long and sometimes is not prolonged above the 2^nd^ floret). The lemmas are usually distinctly keeled (except *Poa secunda*), and commonly are pubescent with hairs on the lemma and/or the callus. The lemma apex and margins are variously narrowly to broadly scareous-hyaline. The lemmas are typically 5-veined but this varies from 3 (*Poa calycina*) to 9 (*Poa mulleri*). The palea surface is thinly herbaceous (unlike *Koeleria*, in which it is hyaline). The callus of the lemma is terete or slightly pinched dorsally, blunt, and fairly indistinct in its transition to the lemma (unlike *Festuca* and *Schedonorus*, which have a dorsally compressed rachilla and a somewhat thick, annulated, glabrous callus, that is sometimes angled downward). The callus typically has an isolated dorsal tuft of woolly hairs called a web, but the callus is glabrous in some taxa (In *Poa secunda* the callus lacks a web but sometimes has a line of short hairs < 0.2 mm long or crown on the callus around the base of the lemma). The lodicules are two in number, usually ovate to lanceolate with a lateral lobe, without distinct vascularization, glabrous, and the upper portion is hyaline. Anthers are 3 in number (sometimes 1 or 2 in *Poa bigelovii*), and within pistillate flowers are typically reduced to staminodes. Ovaries are glabrous with 2 styles that are apical, approximate, plumose, and white. The caryopsis is firm (but known to contain lipid), ventrally sulcate with a short (usually less than 1/7 the grain in length) subbasal hilum, and the embryo is small (less than 1/6 the grain in length).

Chromosome base number is *x* = 7, of medium to medium-large size.

#### Distribution.

The genus is Worldwide but is usually not found in tropical countries without high mountains. In Mexico the genus is known from all states except Nayarit, Quintana Roo, Sinaloa, Tabasco, and Yucatan.

#### Ecology.

Species of *Poa* occur in cool temperate to frigid regions, cool habitats in warm temperate regions, and in dry to wet habitats.

#### Discussion.

*Poa* is a complex genus of more than 500 species with 23 occurring in Mexico. It is the type genus of the family Poaceae, subfamily Pooideae, supertribe Poodae, tribe Poeae, and subtribe Poinae (Soreng et al. 2011). The above synonymy for the genus is the most current available, however, two species of *Aphanelytrum* (and one of *Festuca* related to those) have not yet been formally transferred into *Poa*. *Poa* is known for great diversity in breeding systems (Anton and Connor 1995; Negritto and Anton 2000; Soreng and Keil 2003). In Mexico species may be strictly perfect flowered; gynomonoecious (spikelets with lower florets perfect and upper ones pistillate); possibly sequentially gynomonoecious (some plants shifting to producing more pistillate flowers as the season progresses); dioecious (sexual dimorphism was not evident in the Mexican species); or pistillate only. Apomictic/asexual reproduction by seed is common in some species of the genus, and is sometimes facultative (Clausen 1961, Kellogg 1987), and other times effectively obligate (Soreng and VanDevender 1989; Negritto et al. 2008). In Mexico *Poa chamaeclinos* and *Poa gymnantha* are strictly pistillate apomicts, and *Poa fendleriana* is pistillate and apomictic over much of its range. *Poa alpina*, *Poa compressa*, *Poa pratensis*, and *Poa secunda* are known to be facultatively apomictic elsewhere, but their breeding systems have not been studied in Mexico. *Poa strictiramea* is predicted to be apomictic in Mexico based on the high frequency of sterile anthers. No viviparous/bulbiferous spikelets were found among Mexican specimens. Other relevant literature on *Poa* includes: Soreng (1990), Gillespie and Soreng (2005), Gillespie et al. (2007, 2009), Soreng et al. (2009, 2010), Refulio-Rodríguez et al. (2012) ―molecular data; Soreng (2005) ―chromosome counts; and Tzvelev (1976), Zhu et al. (2006), Soreng (2007), Giussani et al. (2012) ―floristic accounts.

### Key to the species of *Poa* in Mexico

**Table:** 

1	Glumes exceeding the distal florets; lemmas 3 (or 5)-veined	2
–	Glumes shorter than the proximal floret (subequal in *Poa gymnantha*); lemmas 5(or 7)-veined	3
2	Anthers ca. 0.2−0.4 mm long; slender annuals; blades flat, lax; lemma surfaces crisply puberulent; plants from the Pacific coastal islands, Baja California	22. *Poa thomasii*
–	Anthers 0.5−1.1 mm long (i.e., fertile ones, which are often in the proximal floret only); small, densely tufted perennials; blades folded, moderately firm; lemmas glabrous; plants from volcanoes in Central Mexico	5. *Poa calycina* var. *mathewsi*i
3	Plants annual; palea keels distinctly pubescent in part, rarely glabrous but then smooth	4
–	Plants perennial; palea keels glabrous, or sometimes pubescent, but always scabrous in part	6
4	Floret callus with cobwebby hairs; panicles contracted; palea keels with some apical hooks; plants from around the northern deserts	4. *Poa bigelovii*
–	Floret callus glabrous; panicles open; palea keels without hooks; plants from various localities	5
5	Anthers 0.6−1 mm long; panicle branches ascending to spreading, spikelets loosely arranged; plants widespread	2. *Poa annua*
–	Anthers 0.2−0.5(–0.6) mm long; panicle branches ascending, spikelets crowded; plants from Baja California	10. *Poa infirma*
6	Anthers 0.5−1 (rarely to 1.2) mm long (sometimes with only rudimentary staminodes in upper florets within a spikelet, never throughout a spikelet); callus webbed (glabrous in *Poa seleri*)	7
–	Anthers 1.2−3 mm long, or reduced to vestigial staminodes (sacs 0.1−0.2 mm long, with minute filaments, or sacs longer but evidently sterile) in some or all florets; callus glabrous or pubescent	11
7	Panicles contracted, linear; spikelets crowded along branches; plants from central and southern Mexico	18. *Poa scaberula*
–	Panicles more open, eventually ovoid or pyramidal, spikelets remote or moderately crowded along branches	8
8	Lemmas glabrous or base of keel and marginal veins very sparsely puberulent, but glabrous elsewhere	9
–	Lemmas short villous on the keel and lateral nerves, sometimes puberulent between the nerves	10
9	Spikelets lanceolate, florets loosely arranged; glumes unequal, lower glume subulate to wedge shaped, 1/4−1/2 the length of the proximal lemma (rarely longer and narrowly lanceolate); lemma lanceolate, callus glabrous and lemma smooth or slightly scabrous on upper keel; plants from southern Mexico	20. *Poa seleri*
–	Spikelets ovate, florets compactly arranged; glumes subequal, lower glume similar in shape to upper glume, lanceolate to broadly lanceolate in side-view, 2/3−4/5 the length of the proximal lemma; lemma ovate, glabrous or sparsely short puberulent on the keel and marginal veins, callus webbed (sometimes scant and short, and only present on the proximal floret within a spikelet), and lemma usually more scabrous; plants from central and southern Mexico	14. *Poa orizabensis*
10	Ligule of upper culm leaf acute, 3−12 mm long, longer than its blade width; panicles (6–) 12−40 cm long; plants from Coahuila, Madera del Carmen	13. *Poa occidentalis*
–	Ligule of upper culm leaf obtuse or sometimes acute, 1.2−3 mm long, shorter than to equal its blade width; panicles 7−20 cm long; plants from central and southern Mexico	17. *Poa ruprechtii*
11	Plants 1.5−6 cm tall; panicles 1−2 cm long, dense; flowers all pistillate; florets glabrous, lemma body bronze-colored and scareous in the upper 1/4−1/3; plants found in alpine habitats of high elevations (above 4000 m)	12
–	Plants 9−100 cm tall; panicles 2−40 cm long, dense or loose, ovate to pyramidal; flowers perfect-flowered, staminate, or all pistillate; florets pubescent or glabrous, lemma body not or only bronze-colored in a narrow strip, scareous only in the margins and apex; plants found in lowland habitats to low alpine sites	13
12	Ligules abaxially smooth, 0.5−3.2 mm long; blades adaxially smooth or sparsely scabrous; panicles lanceolate to ovate; florets (1−)2; lemmas smooth; plants of wet habitats; plants known from Ixtaccihuatl	6. *Poa chamaeclinos*
–	Ligules abaxially scabrous, less than 1 mm long; blade margins and adaxial surface densely scabrous; panicles linear, spiciform; florets 1−2; lemmas distally finely scabrous; plants of dry habitats; plants known from Monte Tlaloc	9. *Poa gymnantha*
13	Upper glumes (3−)5−7-veined; blades and pedicles finely papilliate (×50); callus glabrous; lemmas loosely pubescent; longest panicle branches 1.4−3.8 cm long; plants 9−28(–42) cm tall; plants from the top of Cerro Potosí, Nuevo León	12. *Poa mulleri*
–	Upper glumes 3-veined; blades and pedicles without papillae (×50); callus pubescent or glabrous; lemmas glabrous or variously pubescent; longest panicle branches (1–)3−15 cm long; plants 20−100 cm tall; plants widespread	14
14	Plant vegetative shoots all intravaginal (cataphylls absent, prophylls well developed 0.5–5 cm long); plants without rhizomes or lateral shoots, densely tufted	15
–	Plant vegetative shoots extravaginal (cataphylls present above rudimentary prophylls up to 0.2 cm long) or mixed extra- and intravaginal (the later with well developed prophylls); plants with or without rhizomes; loosely to densely tufted	18
15	Blades flaccid, flat or sometimes folded, soon withering; sheaths of upper culm leaves closed 5−20% their length; lemmas somewhat rounded on back; plants of Baja California	19. *Poa secunda* subsp.* secund*a
–	Blades moderately firm, flat, folded, and involute on the margins, retaining shape on drying; sheaths of upper culm leaves closed 5−70% their length; lemmas distinctly keeled; plants of Northern Mexico to Mt. Orizaba	16
16	Spikelets ovate; palea keels puberulent to short villous for much of their length; panicles 2−6 (−8) cm long, fairly congested, proximal internode usually 0.6−1 cm long; plants of subalpine to low alpine (province uncertain, possibly Mt. Orizaba)	1. *Poa alpina*
–	Spikelets lanceolate; palea keels glabrous or medially sparsely puberulent; panicles (3−) 4−30 cm long, open or moderately congested, proximal internode 1−5 cm long; plants from uplands of Baja California and in and around the Chihuahuan Desert	17
17	Uppermost culm sheaths (2.5–)10−16 x longer than its blade; flag leaf blade vestigial or reduced, 0.1-1.5(-4) cm long; lemmas pubescent on lower half of the keel and/or keel and marginal veins; longest panicle branches 3−7 cm long; plants 20−50 cm tall; old basal blades commonly disarticulating from dense fascicles of old persisting basal sheaths; plants from the Sierra San Pedro Mártir, Baja California	3. *Poa bajaensis*
–	Uppermost culm sheaths 0.5−1.5 x longer than its blade; flag leaf blade well-developed, 4-23 cm long; lemmas glabrous or very sparsely puberulent on the keel and marginal veins near the base, infrequently sparsely puberulent between the veins near the base; longest panicle branches (1–) 7−10 (–15) cm long; plants (20–) 40−100 cm tall; old basal blades not consistently disarticulating and old sheaths not persisting in dense fascicles; plants from uplands in and around the Chihuahuan Desert	21. *Poa strictiramea*
18	Panicles contracted, fairly dense, or slightly open; uppermost culm leaf blades highly reduced or absent; populations dioecious or pistillate; callus glabrous; plants of the northern mountains	19 (8. *Poa fendleriana*)
–	Panicles loosely contracted to open; uppermost culm leaf blades usually well developed (reduced in *Poa wendtii*); plants all perfect-flowered, or if pistillate or staminate, then panicles open; callus, at least of proximal lemmas, with cobwebby hairs, or glabrous	21
19	Lemmas glabrous; plants from the Sierra Madre Occidental	8a. *Poa fendleriana* subsp. *albescen*s
–	Lemmas pubescent on the keel and marginal nerves	20
20	Ligules of upper culm leaves 0.2−1(–1.5) mm long, truncate to rounded, upper margin minutely ciliate fringed; collar margins usually distinctly scabrous; plants from the range of the species; Baja California, Chihuahua, Sonora, and Coahuila	8b. *Poa fendleriana* subsp. *fendlerian*a
–	Ligules of upper culm leaves (1.5–)1.8–11 mm long, obtuse to acuminate, upper margin without a ciliate fringe; collar margins usually smooth or sparingly scabrous; plants from Baja California	8c. *Poa fendleriana* subsp. *longiligul*a
21	Culms and nodes strongly compressed, keeled, lower nodes usually exposed; plants strongly rhizomatous; plants weedy	7. *Poa compressa*
–	Culms and nodes terete, or culms weakly compressed, lower nodes usually sheathed; plants without rhizomes or rhizomatous; plants weedy or not	22
22	Leafblades mostly involute, mostly 1−2 mm wide (expanded), both surfaces moderately to densely scabrous; callus glabrous (rarely with a few short hairs); lemma keel and marginal veins glabrous or puberulent (hairs to 0.25 mm), sometimes puberulent between the veins near the base; plants from uplands in and around the Chihuahuan Desert	21. *Poa strictiramea*
–	Leafblades flat or folded, some or most more than 2 mm wide, or if involute in part, then surfaces smooth or nearly so; callus with a dorsal tuft of wooly hairs; lemma glabrous or variously pubescent; plants widespread	23
23	Upper culm leaf blade less than 1/10 its sheath in length; lemmas distinctly pubescent between the veins; plants from Sierra Rosario, Coahuila	23. *Poa wendtii*
–	Upper culm leaf blades more than 1/5 its sheath in length; lemmas glabrous or sparsely puberulent between the veins; plants widespread	24
24	Rachilla internodes usually exposed in side view; spikelets 4.5−8 mm long; lemmas glabrous or variously pubescent; ligules truncate to obtuse to acute, sometimes irregularly dentate to lacerate, 0.8−6 mm long; rhizomes usually short and/or poorly developed; plants from the Sierra Madre Occidental and Oriental	25
–	Rachilla internodes mostly hidden from view; spikelets mostly 3−6 mm long; lemmas glabrous between the keel and marginal nerves (sparsely puberulent on the intermediate veins in subsp. *alpigena*); ligules truncate to low rounded, entire, usually 1−2 mm long; plants with an extensive and spreading rhizome system; plants widespread	16. *Poa pratensis*
25	Sheaths closed 30−65% their length (margins partly connected by an invaginated scareous-hyaline membrane for several cm); blades involute, flat or folded, mostly 2−3 mm wide; ligules truncate to obtuse, 0.8–3 mm long; lemmas distinctly puberulent along the keel and marginal veins; plants from the Sierra Madre Oriental	15. *Poa palmeri*
–	Sheaths closed 60−80% their length (margins more or less abruptly connected by a distinct herbaceous connection); blades mostly flat and 3−7 mm wide; ligules obtuse to acute, 3−6 mm long; lemmas glabrous or sparingly puberulent on the lower keel and marginal veins; plants from the Sierra Madre Occidental	26 (11. *Poa matris-occidentalis*)
26	Sheaths of lower leaves smooth, glabrous; collars smooth or with a few hooks, glabrous; lemmas finely muriculate between the veins, keel and marginal veins glabrous below; plants from Durango	11a. *Poa matris-occidentalis* subsp. *matris-occidentali*s
–	Sheaths of lower leaves retrorsely scabrous to puberulent; collars ciliate; lemmas densely scabrous between the veins, keels and marginal veins puberulent below; plants from southern Chihuahua	11b. *Poa matris-occidentalis* subsp. *mohinorensis*

## 1. Poa alpina alpina

L.,Sp. Pl. 1: 67. 1753. subsp.

http://species-id.net/wiki/Poa_alpina_alpina

Fig. 1 A–M

### Type:

Europe, in alpibus Lapponicis, Helveticis (lectotype: LINN-87.2!, designated by Soreng 2000: 254).

*Uralepis mutica* E. Fourn., Mexic. Pl. 2: 110. 1886. Type: Mexico, Liebmann *Gramineae No. 611* (holotype: C, photo US; isotype: US!, right hand culm ex C present in photo taken by A.S.Hitchcock).

*Poa violascens* Phil. Linnaea 29(1): 100. 1858. nom. illeg. hom., non. Cheval. 1827. Type: Chile, Cerca de Arauco, *C. Gay Herb. Chil. 164* (holotype: SGO-PHIL-413!; isotype: SGO-45741!).

### Description.

Hermaphroditic. **Perennials**; tufted, tufts dense, fairly narrow to medium girth, low (mostly less than 8 cm tall), dark green to slightly bluish-green; tillers intravaginal (each subtended by a single elongated, 2-keeled, longitudinally split prophyll), without cataphyllous shoots, sterile shoots more numerous than flowering shoots. **Culms** 10–40 cm tall, erect, sometimes slightly geniculate at base, leaves mostly basal, terete, smooth; nodes 1–2, 1 usually exerted. **Leaves** mostly basal; leaf sheaths terete, smooth, glabrous; butt sheaths persistent, papery, proximal sheaths densely overlapping, persistent; flag leaf sheaths 4–8 cm long, margins fused ca. 12–29% the length, much longer than its blade; collars smooth, glabrous; ligules of upper cauline leaves to 4(–5) mm long, milky white, abaxially smooth, glabrous, apices obtuse, of sterile shoots 1–2(–3) mm long; blades of cauline leaves 1–5(–12) cm long, 2–4.5 mm wide, flat, moderately thick, soft, straight, mostly basal, smooth or margins lightly scabrous, broadly prow-tipped; upper culm blades reduced in length; flag leaf blades ca. 1 cm long; sterile shoot blades widely spreading, persisting through the season. **Panicles** 2–6(–8) cm long, erect, open or loosely contracted at maturity, pyramidal to ovoid, fairly congested, proximal internode 0.6–1(–1.5) cm long; rachis with 1–2 branches per node; primary branches ascending to spreading, straight, to divaricating in flower, terete, smooth or very lightly scabrous, rarely moderately densely scabrous all around; pedicels divaricately spreading, lateral pedicels 1/5–1/2 the spikelet in length, smooth or sparsely to moderately scabrous; longest branches 1–3(–4) cm. **Spikelets** 3.9–6.2 mm long, 1.5–2.5 × long as wide, ovate, laterally compressed, but plump; florets 3–7, rarely bulbous basally and leaf-like distally (in high arctic), hermaphroditic; rachilla internodes terete, 0.5–0.8 mm long, smooth, glabrous or sparsely softly puberulent to short villous; glumes broadly lanceolate to narrowly ovate, distinctly keeled, keels lightly scabrous, acute, lower glumes 3-veined; upper glumes shorter than or subequaling lowest lemma, 3-veined; calluses glabrous; lemmas 3–5 mm long, broadly lanceolate, distinctly keeled, keels and marginal veins short to long villous, between veins sparsely to moderately short villous, intermediate veins moderately prominent, apices acute to obtuse; paleas softly puberulent to short villous over the keels for most of the length, apices scabrous. **Flowers** chasmogamous; lodicules 0.65–0.75 mm long, broadly lanceolate to ovate, with a short lateral lobe; anthers 1.3–2.3 mm long. **Caryopses** 1.9–2.2 mm long, elliptical in side-view, sulcus shallow, brown, hilum 0.25 mm long, round to oval, grain adherent to the palea. {North American counts}. 2*n* = 28, 32, 33, 34, 35, 39, 40+I, 42, ca. 48, 56.

### Distribution.

The species is circumboreal and in North America it ranges from Canada, Greenland, USA, south to Mexico. It is known only from the type collection and the location in Mexico is unknown.

### Ecology.

The species is found in disturbed sites in boreal forests, subalpine to low alpine meadows, and rocky slopes, on calcareous to acidic substrates.

### Conservation status.

The species is common in North America and rare (if extant) in Mexico.

### Discussion.

*Poa alpina* was first reported for Mexico by Hitchcock (1935) and was accepted by Hultén and Fries (1986), Beetle (1977), and Soreng et al. (2003), based solely on the identity of the type specimen of *Uralepis mutica* which Fournier (1886) described in his *Mexicanas plantas* as “Absque loco". The holotype was annotated as “*Uralepis mutica* Fournier" by Fournier, and “Liebm. Pl. México No. 6233" was added to the original ticket at C. In a United States National Herbarium author’s proof copy of Hitchcock (1913), Agnes Chase penciled in a note that the type was determined by A.S. Hitchcock as *Poa alpina*, “but that not in hb. from south of Co[lorado], Verify". The southernmost collections in the United States are from the Sangre de Cristo Range of the Rocky Mountains in northern New Mexico [UNM: Mora and Santa Fe cos.; NMC Taos Co.; in Soreng (2007) the map only shows Taos Co. in New Mexico]. The *Liebmann* specimen might have been collected in the Rocky Mountains in the former Mexican Territory (New Mexico became a territory of the United States in 1848), but we have no evidence of Liebmann collecting there (neither K. Allred nor R.W. Spellenberg have knowledge of Liebmann having collected in New Mexico, pers. comm., 2011). Furthermore, the taxon is rare in New Mexico, having been recorded for the state in only three localities, all post 1980. F.M. Liebmann (b.1813, d. 1856), Danish botanist (main herbarium at C, ca. 90000 specimens) is well known to have collected extensively in central and southern Mexico in Oaxaca, Puebla, and Veracruz between 1841 and 1843 (McVaugh 1987). A few of his collections recorded from other years in Tropicos are likely transcriptional errors (Missouri Botanical Gardens taxonomic database: http://www.tropicos.org/Home.aspx, fide Gerrit Davidse 2012). McVaugh only cites the 1841 to 1843 for Mexican collections. A brief on-line biography (http://www.nathimus.ku.dk/bot/liebmann.htm) of Liebmann does not give any suggestion that he traveled in or collected in the United States, indicating that he traveled to Cuba and Mexico between 1840 and 1843. Although (Harvard University Herbaria) HUH Index to Botanists indicates he also collected in the United States (http://kiki.huh.harvard.edu/databases/botanist_search.php?mode=details&id=1049), it seems unlikely that he would have had an opportunity to collect *Poa alpina* in the USA without having left a substantial record of collections from the country, and such a record seems to be absent. Liebmann did collect on Pico de Orizaba in 1841, and on the highest mountain in Oaxaca, Zempoaltépetl (3397 m), in 1842 (McVaugh 1987), and these seem to be the only places in Mexico the species could have been found by Liebmann between 1841 and 1843. *Liebmann* nos. *603* to *610* are known to be from Orizaba, and *611* might also be from there, however his label numbers are not sequential but were added afterward to organize his collections (McVaugh 1987). The *Poa alpina* specimen could have come into Liebmann’s herbarium and become mixed with Mexican material by some other means. We believe the reputed coastal Arauco, Chile, origin of the type of *Poa violascens* Phil. resulted from a such a mistake by Philippi; see synonymy above. Liebmann also traveled in Norway and Germany and had substantial material from other botanists. Nevertheless, it is possible for a common species like *Poa alpina* to have become established this far south in Mexico.

**Figure 1.**
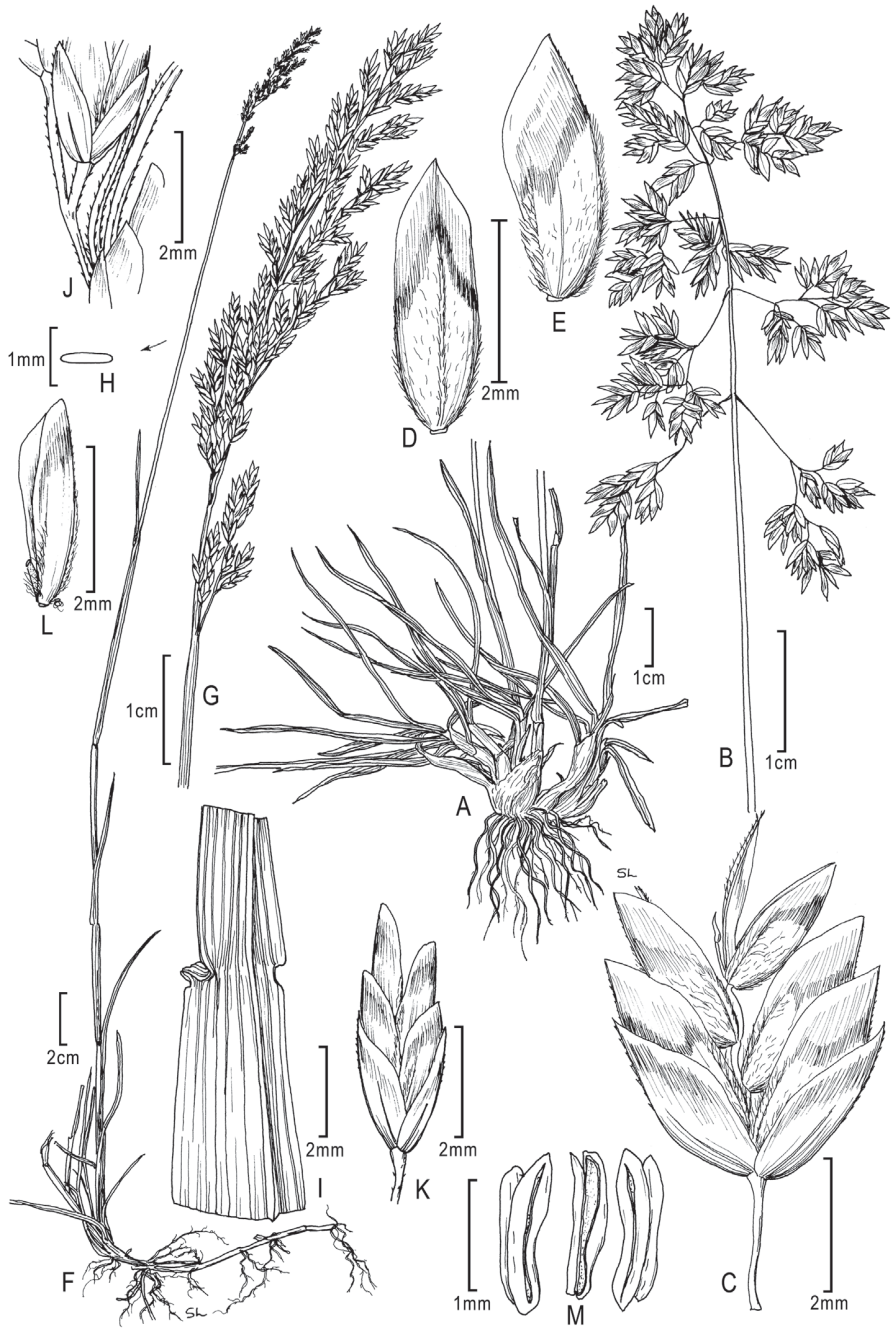
**A–E** *Poa alpina*L. **A** basal tuft **B** inflorescence **C** spikelet **D** floret dorsal view **E** floret lateral view; **F–M** *Poa compressa*L. **F** habit **G** inflorescence **H** culm cross-section outline **I** sheath, collar, blade abaxial view **J** branch segment with spikelet **K** spikelet **L** floret **M** anthers. **A–M** from Soreng (2007) **E** originally drawn from *Eggleston 11824* in Hitchcock (1935).

## 2. Poa annua

L., Sp. Pl. 1: 68 1753.

http://species-id.net/wiki/Poa_annua

Fig. 2 A–E

Ochlopoa annua (L.) H. Scholz, Ber. Inst. Lanschafts- Pflanzenokologie Univ. Hohenheim Beih. 16: 58. 2003. Type:Habitat in Europa ad vias. (lectotype: LINN-87.17!, right-hand plant, designated by Soreng 2000: 254).

### Description.

Gynomonoecious. **Annuals** (rarely surviving for a second season); tufted, infrequently short stooling, tufts generally small, bases usually narrow, green to light green; tillers intravaginal (each subtended by a single 2-keeled, longitudinally split prophyll over 0.5 cm long), without cataphyllous shoots, most shoots eventually flowering. **Culms** 2–20(–45) cm tall, spreading to erect, sometimes geniculate, leafy, slender, terete, smooth; usually 1 nodes. **Leaf** sheaths terete or weakly compressed, smooth, glabrous; butt sheaths papery, smooth glabrous; flag leaf sheaths 1–6 cm long, margins fused ca. 33% the length; throats and collars smooth, glabrous; ligules 0.5–3(–5) mm long, decurrent, abaxially smooth, glabrous, apices obtuse to truncate; blades 1–10 cm long, 1–3(–6) mm wide, flat or weakly folded, thin, soft. smooth, margins usually slightly scabrous, broadly prow-tipped; blades all about equal in length, flag leaf blades well developed. **Panicles** 1–7(–10) cm long, 1.2–1.6 × long as wide, erect, open, pyramidal to rhomboid, moderately congested to sparse; rachis with 1–2(–3) branches per node; primary branches ascending to spreading or reflexed, straight, terete or sulcate, smooth; lateral pedicels less than 1/3 the spikelet in length, smooth; longest branches 2–4(–5) cm, spikelets crowded or loosely arranged. **Spikelets** 3–5 mm long, ovate, laterally compressed; not bulbiferous; florets 2–6, proximal hermaphroditic, distal often pistillate; rachilla internodes terete, smooth, glabrous, more or less concealed or exposed, distal internode less than 1/2(–3/4) length of distal lemma; glumes unequal, smooth, distinctly keeled, keels smooth; apex acuminate to acute or obtuse, sharp pointed or slightly blunt; lower glumes 1–2.2 mm long, 1-veined, narrowly lanceolate, often slightly sickle shaped, or subulate, apex acute; upper glumes 1.5–2.5 mm long, shorter than or subequaling lowest lemma,3-veined, lanceolate to oblanceolate, apex obtuse to acute; calluses glabrous; lemmas 2.2–3.3(–4) mm long, broadly lanceolate, green to light green, sometimes slightly anthocyanic, distinctly keeled, smooth throughout, keels, marginal, and usually intermediate veins moderately to densely crisply puberulent to long villous, rarely glabrous throughout, between veins glabrous, intermediate veins prominent, margins and edges smooth, apices obtuse to acute; paleas keels smooth, short to long villous, rarely glabrous over the keels. **Flowers** cleistogamous to chasmogamous; lodicules 0.45–0.5 mm long, broadly lanceolate to ovate, with 1 (rarely 2) short lateral lobe(s); anthers 0.6–1.1 mm long, oblong prior to dehiscence, often vestigial in distal flowers of a spikelet. **Caryopses** 1.6–1.8 mm long, elliptical in side-view, subcylindrical in cross-section, light brown, sulcus almost flat, hilum 0.15 mm long, oval, grain free or adherent to the palea. 2*n* = 28.

### Distribution.

The species is distributed Worldwide, primarily in temperate and subtropical regions. In Mexico it is recorded from all states, except Campeche, Nayarit, Quintana Roo, Sinaloa, Tabasco, and Yucatán.

### Ecology.

The species is a gynomonoecious, ruderal annual (infrequently a short-lived perennial), and is found in waste ground, lawns, gardens, trails, sidewalks, roadsides, fields ranging from sea level to over 4500 m. The species can potentially flower throughout the year.

### Specimens examined.

Mexico. **Aguascalientes:** Municipio Aguascalientes, Aguascalientes, 18 Sep 1978, J.A.Zamarripa-D. (MEXU). **Baja California:** Guadalupe Island, North Twin Cañon, 50 m, 24 Apr 1958, R.Moran 6622 (US). **Baja California Sur:** reported by Espejo Serna and Lopez Ferrari (2000). **Chiapas:** Escuintla, 3 Oct 1936, E.Matuda 312 (US). **Chihuahua:** vic. of Chihuahua, 1300 m, 27 Apr 1908, E.Palmer 28 (US). **Coahuila:** Saltillo, Apr 1898, E.Palmer 6 (US). **Colima:** reported by Espejo Serna and Lopez Ferrari (2000). **Durango:** Durango, Apr 1896, E.Palmer 97 (TAES, US). **Distrito Federal:** Azcapotzalco, 27−30 Jul 1910, A.S.Hitchcock 5929 (US). **Guanajuato:** Municipio Cortazar, Ejido del Rancho del la Gavia, 2330 m, 9 Oct 1996, s. collector (MEXU). **Guerrero:** Municipio Tlacotepec, Cerro Teotepec, 3350 m, 5 Dec 1963, J.Rzedowski 18145 (MEXU, MSC). Municipio General Heliodoro Castillo, El Jilguero, 2600 m, 30 Oct 1998, N.Diego 8146, B.Ludlow & A.Acosta (MEXU, lemmas glabrous or nearly glabrous). **Hidalgo:** Pachuca, 6−7 Sep 1910, A.S.Hitchcock 6747 (US). **Jalisco:** Mt. Nevada, 23−24 Sep 1910, A.S.Hitchcock 7154 (US). **Michoacán:** 8−10 mi NW of Ciudad Hidalgo, 2850−3000 m, 18 Mar 1949, R.McVaugh 9902 (US). Municipio Tallpujahua, Presa Brockman, 2760 m, I.García 3603 & Y.H.-deG (MEXU, stooling form). **Morelos:** Morelos, El Parque, 31 Aug 1910, C.R.Orcutt 3858 (US). **Mexico:** woods near El Oro, 9000 ft [2745 m], 14 Sep 1901, C.G. Pringle 9589 (TAES).Ixtaccihuatl, Oct 1905, C.A.Purpus 1618 (US). **Nuevo León:** Cerro Potosí, 9000 ft [2745 m], 7 Jul 1963, R.L.McGregor 267, L.J.Harms, A.J.Robinson, R.del Rosario, R.Segal (US). **Oaxaca:** Oaxaca, 5000 ft [1525 m], 30 May 1894, C.G.Pringle 4671 (US). **Puebla:** Tehuacán, 9 Aug 1910, A.S.Hitchcock 6049 (US), Ixtaccihuatl S flank, in circ E of the “portal" N of La Amacuilecatl “Los Pies", 19.1543°N; 98.6307°W, 4400 m, 2 Oct 1987, R.J.Soreng 3323 & N.Soreng (US, stooling form). **Querétaro:** Querétaro, 24−25 Jul 1910, A.S.Hitchcock 5829 (US). **San Luis Potosí:** Alvarez, 28 Sep to 3 Oct 1902, E.Palmer 173 (US). **Sonora:** Guadalajara, Jul−Oct 1886, E.Palmer 483 (US). **Tamaulipas:** Reported (Espejo Serna and Lopez Ferrari 2000). **Tlaxcala:** Municipio A. Arista, 5 km al sur de San. Felipe Hidalgo, 3000 m, 15 Dec 1988, R.Acosta 2689 & M.Sánchez (MEXU). **Veracruz:** Jalapa, 4600’ [1400 m], 2−4 Sep 1910, A.S.Hitchcock 6625 (US). **Zacatecas:** near Plateado, 1 Sep 1897, J.N.Rose 2712 (US).

### Discussion.

This Old World species is naturalized throughout temperate regions of the New World. The species was already widespread in Mexico by the mid-1800’s: Popocatepetl, *H.G. Galeotti 5828*, *5854* (US fragm. ex CAEN in 1837−1847); Orizaba, *F. Müller 2094* (US fragm. ex P in the 1850’s); and San Luis Potosí, *M. Virlet-d’Aoust 1372*, *1394* (US fragm. ex P in 1851). Above we list a single specimen per state for this species, except when there is notable morphological variation. We cite the oldest voucher seen that came from the state. The species probably occurs sporadically in additional states and is surely in Sinaloa but without a collection. Plants from wet areas at high altitudes can perenniate by rooting at the nodes (noted in specimens cited from Michoacán and Puebla), and such forms have been called *Poa annua* var. *reptans* Hausskn. We suspect that such forms arise repeatedly in plastic response to the environmental conditions, rather than from unique genetic variation, and are not particularly useful to recognize taxonomically. A form with the lemmas and paleas glabrous or nearly glabrous occurs sporadically around the world, and has been found in Guerrero. All collections from Mexico we have seen identified as “*Poa infirma*" belong to *Poa annua*, and only one specimen was redetermined by us as *Poa infirma* from among hundreds of *Poa annua* specimens. DNA data support the hypothesis that *Poa annua*, a tetraploid species, arose from hybridization between two Eurasian diploid species, *Poa infirma* × *supina* Schrad. (Soreng et al. 2010). *Poa infirma* is a short-lived annual and *Poa supina* is a stoloniferous perennial. Species of this complex [*Poa* sect. *Micrantherae* Stapf; syn. *Poa* sect. *Ochlopoa* Asch. & Graebn., *Ochlopoa* (Asch. & Graebn.) H. Scholz.] have spikelets with perfect lower florets and pistillate upper florets, smooth branches and spikelet bracts, and soft puberulent lemma keels and marginal veins, soft puberulent palea keels, and glabrous calluses. *Poa annua* frequently approaches *Poa infirma* in form, presumably in part due to expression of particular genes, or segregation of those genes toward the parental species. This hypothesis explains the difficulty of identifying true diploid *Poa infirma*. In a separate review of US New World specimens previously identified as *Poa annua* or *Poa infirma* by Hildemar Scholz (B, dets. of 2007), only one specimen from Central America was identified as other than *Poa annua* (see *Poa infirma* discussion below).

**Figure 2.**
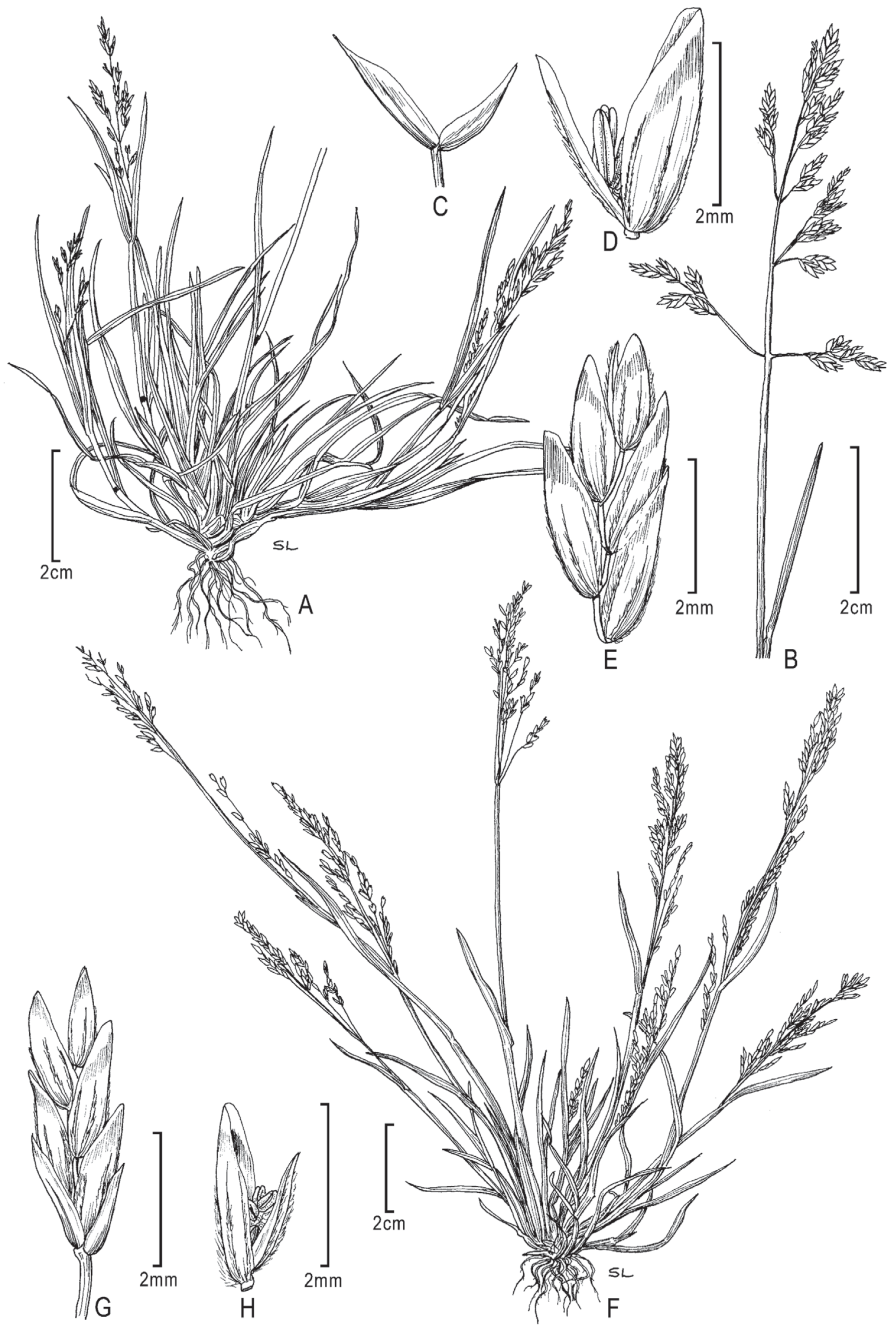
**A–E** *Poa annua*L. **A** habit **B** inflorescence **C** glumes **D** floret with palea and anthers **E** florets **F–H** *Poa infirma* Kunth **F** habit **G** spikelet **H** floret with palea and anthers. Drawings from Soreng (2007).

## 3. Poa bajaensis

Soreng, Madroño 48(2): 123, f. 1. 2001 [2002].

http://species-id.net/wiki/Poa_bajaensis

Fig. 3 A, B

### Type:

Mexico, Baja California, Sierra San Pedro Mártir, E rim above Yerba Buena, 31°01'N, 115°W, 2700 m, 1 Jun 1968, *R. Moran 15070* (holotype: US-259736!; isotypes: SD-69304!, TAES-16825!).

### Description.

Hermaphroditic. **Perennials**; tufted, tufts dense, fairly narrow to medium girth and low to medium height (5–12 [–15] cm tall), pale green to bluish green; tillers intravaginal (each subtended by a single elongated, 2-keeled, longitudinally split prophyll), without cataphyllous shoots, sterile shoots more numerous than flowering shoots. **Culms** 20–50 cm tall, erect, blades strongly reduced upward, terete, smooth; nodes 2–3, smooth, upper (0–)1–2 exerted, uppermost at mid-culm. **Leaves** mostly basal; leaf sheaths slightly keeled, very sparsely to moderately (rarely densely, evenly, finely scabrous; butt sheaths persisting, grayish-brown, papery, their blades disarticulating, the bases smooth, glabrous; flag leaf sheaths 8–15 cm long, margins fused 29–36% their length, (2.5–)10–16 × longer than its blade; collars vestiture like that of the sheath; ligules 0.25–0.5 mm long on basal leaves, to 0.5–1.5(–2) mm long on upper culm leaves, abaxial surface and upper margins densely scabrous, apices truncate to obtuse; blades to 10(–15) cm long, 1.5–2.75 mm wide, folded or flat, margins inrolling, moderately thick abaxially smooth or lightly scaberulous (denser apically), margins scabrous, adaxially smooth or moderately to densely scaberulous, narrowly and abruptly prow-tipped; culm blades sharply reduced upward, flag leaf blades 0.1–1.5(–4) cm long. **Panicles** 4–13 cm long, erect, open, pyramidal, well exerted, sparse, with 16–50 spikelets, peduncles smooth, proximal internode 1.8–3.9(–5.2) cm long, smooth; rachis with branches 2–3(–5) per node; primary branches widely spreading to reflexed, fairly strict, terete to weakly angled, smooth or sparsely scaberulous proximally, smooth or moderately (rarely densely) distally, hooks not confined to rows on angles; lateral pedicels mostly about half the spikelet length, smooth or sparsely scabrous, prickles fine; longest branches 3–7 cm, with 3–15 spikelets in the distal 1/2. **Spikelets** 3.75–8 mm long, lanceolate, laterally compressed not bulbiferous, pale green to strongly anthocyanic; florets (1–)2–4(–6), hermaphroditic; rachilla internodes terete, 1.25–2 mm long, smooth, glabrous; glumes, lanceolate to narrowly lanceolate unequal to subequal, shorter than the first lemma, distinctly keeled, thin, smooth or lightly scabrous distally, lateral veins and apical surfaces smooth or lightly scabrous, sharply acute; lower glumes 2.5–3 mm long, ca. 1/2 the adjacent lemma in length, 1(–3)-veined; upper glumes 2.8–3.5 mm long, 3-veined, often 2 × wider than the lower one, ca. 3/4 the adjacent lemma in length, with a broad scarious-hyaline margin; calluses glabrous or webbed, web sparse, hairs to 2 mm long, woolly; lemmas 3.2–4.2 mm long, lanceolate, 5 veined, green to strongly anthocyanic, distinctly keeled, (rarely glabrous throughout), keels to 4/5 and marginal veins to 1/2 sparsely to densely sericate, between veins glabrous or sparsely puberulent in lower 1/2, smooth except for hooks on the upper keel, intermediate veins faint, margins scarious hyaline, edges smooth or with a few fine hooks, apex acute; paleas finely and closely scabrous, glabrous or in some plants medially sparsely softly puberulent, intercostal area glabrous or sparsely puberulent. **Flowers** chasmogamous; lodicules 1 mm long, lanceolate, apex obtuse, unlobed; anthers 1.7–3.2 mm long (rarely sterile, but then 1.7–1.8 mm long). **Caryopses** 1.9–2.3 mm long, elliptical in side-view, compressed, light honey-brown, sulcus narrow, shallow, hilum 0.25 mm long, oval, grain free from the palea. 2*n* = unknown.

### Distribution.

The species is known only from Baja California and is endemic to Sierra San Pedro Mártir.

### Ecology.

This species occurs on mountain slopes, flats, and drainages, and is associated with *Salix* and *Populus tremuloides* Michx. thickets, *Quercus*−*Pinus jeffreyi* forests, and *Quercus*−*Pinus jeffreyi*−*Poa lambertiana*−*Abies concolor* forests, and open meadows in *Pinus jeffreyi* Balf. forests. It is found in sandy to rocky to clay, granitic, often duff-covered soils, between 1450−2950 m. Flowering May to June.

### Conservation status.

The species is a narrow endemic and locally frequent.

### Specimens examined.

Mexico. **Baja California:** Sierra San Pedro Mártir crest of range N of observatory, head of Cañada el Copal and S slope of Cerro Venado Blanco, 2500−2700 m, 3 Jun 1988, S.Boyd 2311, T.Ross, K.McCulloh (RSA). La Concepcíon, 31°01'N, 115°37'W, ca. 1450 m, 31 May 1968, R. Moran 15006 (SD, TAES). open W slope of Cerro 2828, ca. 31°02'N, 115°27'W, ca. 2800 m, 31 May 1968, R. Moran 15060 (BH, SD, TAES). 2 mi W of Vallecitos, 31°00'N, 115°29'W, ca. 2250 m, 2 Jun 1968, R. Moran 15083 (SD, TAES). 3 km NE of El Alto de Corona, 31°00'N, 115°41'W, ca. 2400 m, 20 Aug 1977, R. Moran 24555 (SD). W slope below summit of El Picacho, 30°59'30"N, 115°22'30"W, ca. 2950 m, 5 May 1978, R. Moran 25611 (SD, TAES). end of road into high end of northern sierra, ca. 64 mi. from end of paved road to Ensenada, 7200 ft [2210 m], 6 Jun 1962, J.D.Olmsted 4561 (RSA; somewhat intermediate to *Poa secunda*). central [region], ca. 3 mi ESE of Prado del Corona, ca. 1 mi up canyon from southernmost aspen colony, tributary of Rio San Rafael, 8100 ft [2490 m], 9 Jun 1962, J.D.Olmsted 4711 (RSA). S of Vallecitos near Cerro la Botella Azul, 30°57'20"N; 115°25'26"W, ca. 2440 m, 27 Jun 1998, J.Rebman 5384 & A. Russell (US). near crest of mountain range, approx. 2 mi SE of the Observatory, 31°14'N, 115°64'W, ca. 2985 m, 28 Jun 1998, J.Rebman 5384 & A.Russell (SD); “Corral Meadow", 7.5 km NW (340°) of the Observatory, 31°06'45"N, 115°29'50"W, 16 Jun 1988, A.C.Sanders 7895, R.Minnich, E.Franco, M.Salazar (RSA, SD, TAES). Vallecitos, ca. 31°02'N, 115°28'W, ca. 2430 m, 18 Jun 1985, R.F.Thorne 60858, R.Dahlgren, S.Boyd & D.Charlton (MEXU, RSA,SD); ditto, ca. 31°02'N, 115°27.5'W, ca. 2430 m, 1 Sep 1985, R.F.Thorne 61394, M.Z.Thorne, L.Thorne & T.Petrella (RSA). just above Observatory living quarters, ca. 31°02'N, 115°28'W, ca. 2600 m, 7 May 1986, R.F.Thorne 61967, T.S.Elias & P.Rojas (MO-3333160, RSA); near gate to UNAM Observatory, 31°02'N, 115°29'30"W, 2520 m, 29 May 1982, G.Yatskievych 82–190, S.Forbes, M.Gallagher, J.Evans & A.Kelley (SD). 55 mi SE of highway 1 on road towards Villecentos at Arroyo Los Alamillos, 31°0.67'N, 115°32.28'W, 2270 m, 24 Sep 2000, P.M.Peterson 15189 & J.Cayouette (US, not flowering). 62 mi SE of highway 1 towards on road towards Villecentos, 31°1.99'N, 115°20.35'W, 2490 m, 24 Sep 2000, P.M.Peterson 15197 & J.Cayouette (US, not flowering).

### Discussion.

The species is endemic to the upper elevations of the Sierra San Pedro Mártir, Baja California but apparently was not collected until 1962. Reported and described as *Poa orcuttiana* Vasey (= *Poa secunda*) by Gould and Moran (1981); the five collections they mentioned are all *Poa bajaensis*. Specimens were sometimes labeled as “*Poa interior* Rydb." (e.g., at SD and TAES). It is well marked by its sparse open panicles, short flag leaf blades, and dense fascicles of old persisting sheaths from which the blades tend to disarticulate.

**Figure 3.**
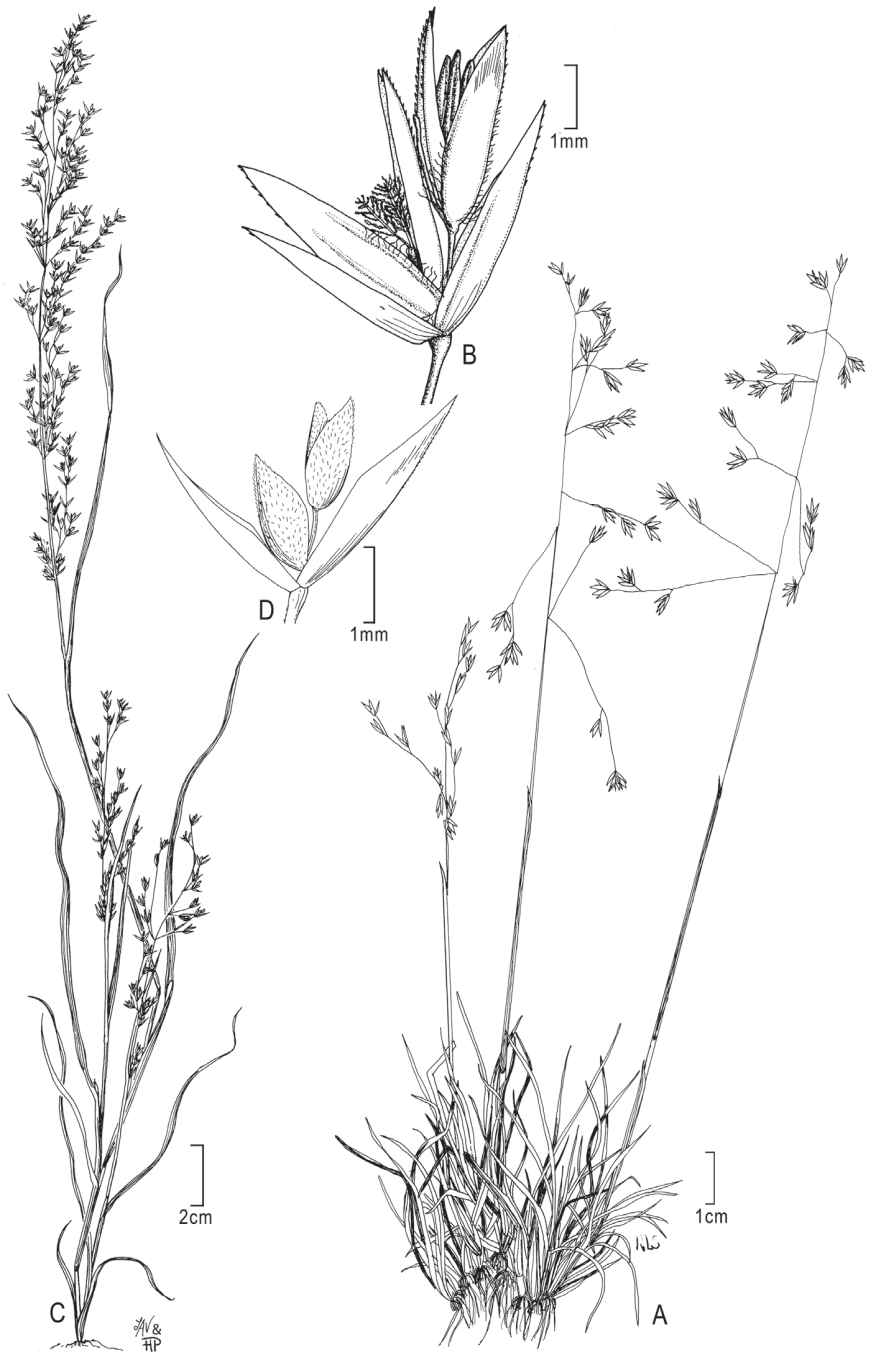
**A, B** *Poa bajaensis* Soreng **A** habit **B** spikelet **C, D** *Poa thomasii* Refulio **C** habit **D** spikelet. **A, B** from Soreng (2002), drawn from holotype collection (*Moran 15070*) **C, D** from Refulio-Rodríguez (2007) as *Dissanthelium californicum*, originally drawn from *Trask 324* in Hitchcock (1935).

## 4. Poa bigelovii

Vasey & Scribn., Descr. Cat. Grass. U.S. 81. 1885.

http://species-id.net/wiki/Poa_bigelovii

Fig. 4 A–E

Poa annua var. *stricta* Vasey ex Scribn., Bull. Torrey Bot. Club 10(1): 31. 1883. Type: USA, Arizona, banks of the Rillita, 9 Apr 1881, *C.G. Pringle s.n*. (holotype: US-81668!; isotypes: NY-431203!, NY-431204!, US-723316!, US-824699!, US-914298!).
Poa bigelovii Vasey & Scribn., Contr. U.S. Natl. Herb. 1(8): 270. 1893 (isonym). Type: USA, New Mexico, 3 Jul 1847, *A. Fendler 931* (holotype: US!; isotypes: MO-3048732!, MO-3048575!, NY-431217!,S-G-4935!).

### Description.

Hermaphroditic. **Annuals** (rarely longer-lived); tufted, tufts sparse, small, bases narrow, green; tillers intravaginal (each subtended by a single elongated, 2-keeled, longitudinally split prophyll), without cataphyllous shoots, most shoots flowering. **Culms** (2–)5–60(–70) cm tall, erect or infrequently geniculate at base, slender, leafy, slightly compressed, smooth (or lightly scabrous above); nodes terete, 2–4, usually 1–2 exerted eventually. **Leaf** sheaths usually compressed and keeled, smooth or keels scabrous; butt sheaths papery, smooth, glabrous; flag leaf sheaths 2–10 cm long, margins fused 25–50% their length, 4 × longer to slightly shorter than its blade; throats and collars smooth, glabrous; ligules 2–6 mm long, usually decurrent, abaxially smooth or scabrous, apices obtuse to acute; blades of cauline leaves (1–)4–15 cm long blades 1–5(–6.5) mm wide, flat or folded, thin, soft, finely scabrous, broadly prow-tipped; flag leaf blade 1–7(–13) cm long, flag leaf blades 1–4(–8) cm long. **Panicles** (1–)5–15 cm long, mostly over 8 × longer than wide, erect, contracted, cylindrical, sometimes interrupted, congested to loosely congested; rachis with 2–3(–5) branches per node; primary branches erect or steeply ascending, more or less angled, sparsely to densely scabrous; lateral pedicels mostly less than 1/4 the spikelet in length, moderately scabrous, prickles moderately coarse; longest branches 1–7 cm, with 3–15(–25) spikelets in the distal 1/4–1/2. **Spikelets** 4–7 mm long, ovate to lanceolate, laterally compressed; not bulbiferous; florets 3–7, hermaphroditic; rachilla internodes terete, up to 1 mm long, smooth, glabrous; glumes narrowly lanceolate to lanceolate, subequal, distinctly keeled, keels and sometimes lateral veins scabrous, acute to acuminate; lower glumes 2.2–3.8 mm long, 1–3-veined; upper glumes 2.5–4.8 mm long, subequaling lowest lemma or slightly exceeding it, 3-viened; calluses dorsally webbed, web distinct, hairs woolly; lemmas 2.6–4.3 mm long, lanceolate, green to light green, sometimes slightly anthocyanic, distinctly keeled, smooth, keels to near the top, marginal veins to 2/3, and sometimes intermediate veins short to long villous, between veins glabrous or softly puberulent, intermediate veins obscure to moderately prominent, upper margins white to hyaline, apices acute, pointed or slightly blunt; paleas keels distally with a few hooks, medially softly puberulent to short villous over the keels, intercostal region usually softly puberulent. **Flowers** mainly cleistogamous; lodicules 0.45 mm long, lanceolate, with a slight lateral lobe at middle; anthers (1–2–)3, 0.2–1.0 mm long. **Caryopses** 1.8 mm long, narrowly elliptical in side-view, laterally compressed, light brown, sulcus narrow, hilum 0.3 mm long, elliptical, grain adherent to the palea. 2*n* = 28, 28+I.

### Distribution.

The species is endemic to the southwestern USA (Arizona, California, Colorado, Nevada, New Mexico, Oklahoma, Texas, and Utah) and northern Mexico. In Mexico it occurs in Baja California, Baja California Sur, Chihuahua, Coahuila, Nuevo León, and Sonora.

### Ecology.

The species occurs in shady sites among rocks and shrubs, usually in loose soils in springtime-moist desert uplands to open slopes lower *Pinus*-*Quercus* forests. This species is associated with the Chihuahuan, Sonoran, and Mojave desert vegetation. It occurs between 420–2200 m, and possibly higher. Flowering January to May.

### Specimens examined.

Mexico. **Baja California:** El Rancho Viejo, 30 Apr 1889, J.S.Brandegee 24 (US). hills behind Bahia de Los Angeles, 3700 ft [1130 m], 24 Feb 1963, R.S.Cowan 2344 (MEXU). Sierra Juárez, 32°27.5'N, 116°09.25'W, 1300 m, 8 May 1982, R.Moran 30600 (MEXU). Sierra La Libertad, Canyon La Borreguera, 9.5 miles E of Mission San Borja, 28°50'N, 113°40'W, 2400 ft [730 m], 27 Mar 1978, K.Nixon 1130, C.P.Cowan & M.L.Sauls (MEXU, TEX). Arroyo San Pedro near La Bocana, 28°28'N, 113°25'W, 250 m, 11 Mar 1966, R.Moran 12512 (TAES). **Baja California Sur:** N slope of Cerro Azufre, 27°30'N, 112°36'W, 1650 m, 14 Apr 1973, R.Moran 20509 (MEXU, TAES); ditto, 17 Feb 1973, R.Moran 20173 & J.L.Reveal (TAES). Volcán las Tres Vírgenes, 1150 m, R.Moran 20411 (TAES, TEX, US). **Chihuahua:** ca. del Rio Chihuahua, 5 Apr 1886, G.G.Pringle (MEXU). Mountains. near Paso del Norte, G.R.Vasey s.n. (US). Canon de Tinaja de Corazon, east flank of Sierra de Mulato on Rio Grande 29°13'N, 103°45'W, 750 m, 13 Mar 1986, M.C.Johnston 12898 (TEX). canyon in north face of Sierra Rica, south of Rancho La Consolacion, 19°12'N, 104°05'W, 1400–2000 m, 3 May 1973, M.C.Johnston 10776 (LL). **Coahuila:** Saltillo, 9 Apr 1905, E.Palmer 532 (NY, US). ca. 2 km N of Estacion Carneros, E flank of Sierra El Chorreadero, 25°08'00"N, 101°07'20"W, 2150 m, 29 Mar 1973, M.C.Johnston 10498, T.L.Wendt & F.ChiangC. (MEXU, MO, TEX). Rio Grande, Tule Canyon above Upper Madison Falls, 29°44−45'N, 102°23'30"W, 475 m, 10 Apr 1973, M.C.Johnston 10618, T.L.Wendt & F.Chiang-C. (MEXU, TEX). Municipio Ramos Arizpe, Sierra de la Paila, 5 Jan 1966, Cano 15 (TAES). [Sierra de Santa Rosa], Rincón de María, on Hacienda La Babia, ca. 70 rd. mi. NW from Múzquiz, 28°27'30"N, 102°05'W, 1900 m, southwest part of rincón, southsouthwest of “Slump Spring", 27 Apr 1975, T.Wendt 901A & D.Riskind (LL). Sierra del Carmen, Rancho Moreros y Rancho San Isidro, ca 178 km de Múzquiz por la brecha Músquiz-Boquillas del Carmen (carretera 53), 28°47'N, 102°30'W, 1300 m, 27 Mar 1992, M.A. Carranzal 398, J.Noriega & L.García (TEX). **Nuevo León:** Monterey, 17−26 Feb 1880, E.Palmer 1365 (US). Sierra Madre above Monterey, 3000 ft [misprint for 8000 ft? = 2440 m], 31 Mar 1906, C.G.Pringle 13748 (MO, TEX, US). **Sonora:** N end of Sierra El Viejo, in the large canyon containing Mina Santa Cruz, 25 miles SW of Caborca, vic. 30°22'N, 112°22'W, 1700 ft [520 m], 7 Mar 1983, A.C.Sanders 3527, M.Dimmitt & G.Montgomery et al. (MEXU). SW of Sonoyta on Mexico Highway 8, 31°50'N, 112°56'W, 420 m, 30 Mar 1988, R.S.Felger 88-151 & A.D.Zimmerman (MEXU). Municipio Yecora, 3.5 km W of Santa Ana road, 4.8 km E of San Nicolas road, on Mexico 16, 28°25'40"N, 109°08'50" W, 720 m, 31 Mar 1997, A.L.Reina-G. 97-466 & T.R.VanDevender (MEXU, TEX). 1.5 km SW of Santa Ana on road to Guadalupe Tayopa, 28°22'54"N, 109°09'30"W, 775 m, 21 Feb 1997, T.R.VanDevender 97-207 & A.L.Reina-G. (MEXU, TEX).

### Discussion.

Presumably because of its early spring flowering and dependence on winter and early spring precipitation, this species is infrequently collected in Mexico.Its annual habit and narrow, congested panicles, combined with a web on the callus of the lemma, distinguish it from all other species of *Poa*. The *Cano 15* specimen from Coahuila is unusual because the panicle is open and the branches are naked in the lower half, but otherwise it aligns near *Poa bigelovii*. It was originally determined as “*Poa chapmaniana* Scribn.?" (but it has 3 anthers ca. 1 mm long, versus 1 ca. 0.2 mm long), then as *Poa annua* (by Gould in 1966), but it is quite scabrous on the branches and has a strong web on the callus like *Poa bigelovii*. Plants from Municipio Ramos Arizpe should be examined more closely as a possible new species.

**Figure 4.**
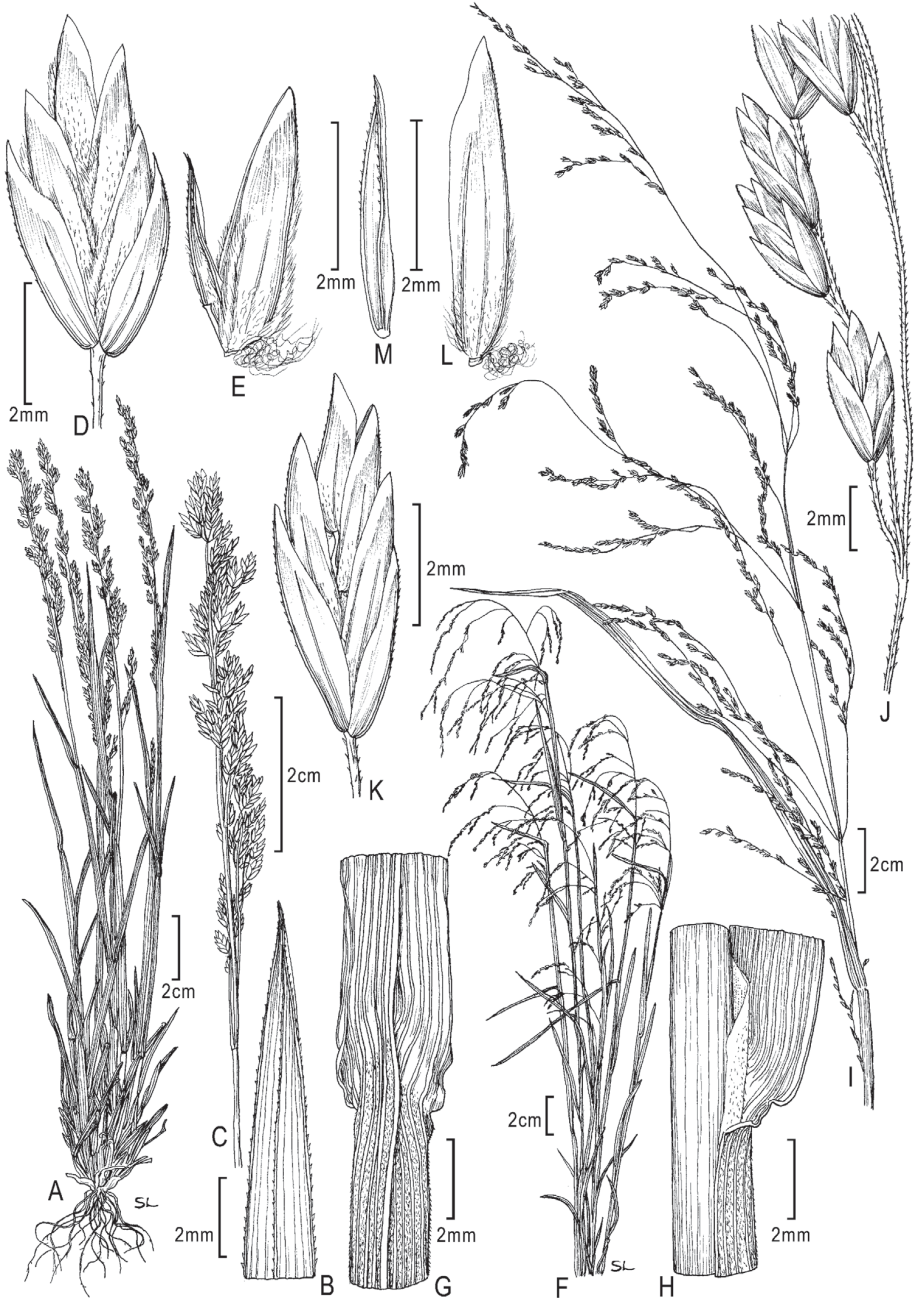
**A–E** *Poa bigelovii* Vasey & Scribn. **A** habit **B** blade apex abaxial view **C** inflorescence **D** spikelet **E** lemma and palea **F–M** *Poa occidentalis* (Vasey) Vasey **F** habit **G** sheath, collar, blade, abaxial view **H** sheath, ligule, blade **I** inflorescence **J** branch segment with spikelets **K** spikelet **L** floret **M** palea. Drawings from Soreng (2007).

## 5. Poa calycina mathewsii

(Ball) Refulio, Syst. Bot. 37(1): 130. 2012.

Figs 5 6 N–P

Deschampsia mathewsii Ball, J. Linn. Soc., Bot. 22: 60. 1885. *Dissanthelium calycinum* subsp.* mathewsii* (Ball) Soreng, Novon 8(2): 201 1998. Type: Peru, above Caspalta, 14000−14300 ft [4270−4360 m], 22 Apr 1882, *J. Ball s.n*. (holotype: K!; isotypes: GH!, US-908709! fragm. ex K).
Dissanthelium sclerochloides Steud. ex E. Fourn., Mexic. Pl. 2: 112 1886. Type: Mexico, Nevado de Toluca, 1865–1866, *M. Hahn s.n.* (lectotype: P!, designated by Soreng 1998: 201; isolectotypes: P!, US-865890! fragm. ex P-Fourn-258).
Dissanthelium semitectum Swallen & Tovar, Phytologia 11: 370 1965.Type: Peru, Junín, Huaron, in clumps on northeastern rock ledges, about 14000 ft [4270 m], 12 Jun 1922, *J.F. MacBride & W.Featherstone 1155* (holotype: US-1161061!).

### Description.

Gynomonoecious. **Perennials**; tufted, tufts dense, small, low (mostly 3–8 cm tall), with panicles mostly included among the leaves, pallid green to bluish-grey-green, sub-lustrous; tillers intravaginal (each subtended by a single elongated, 2-keeled, longitudinally split prophyll), without cataphyllous shoots, sterile shoots more numerous than flowering shoots. **Culms** 4–9 cm tall, erect or ascending, sometimes slightly decumbent or geniculate, leafy, terete, smooth; nodes 0–1, not exerted. **Leaves** mostly basal; leaf sheaths slightly compressed, smooth, glabrous, lustrous; butt sheaths papery, smooth, glabrous; flag leaf sheaths 1.5–4.5 cm long, margins fused ca. 30% their length, ca. equaling its blade; throats and collars smooth, glabrous; ligules (0.5–)1–2.5 mm long, hyaline, abaxially smooth or scabrous, apex obtuse to acute, entire to dentate, sterile shoot ligules like those of the culm leaves; blades 1–6 cm long, 1.5–3 mm wide (expanded), folded, often with strongly involute margins, moderately thick and firm, abaxially smooth sub-lustrous, veins slightly expressed, margins scabrous, adaxially smooth or moderately to densely scaberulous, apex slender prow-tipped; flag leaf blades 1–6 cm long; sterile shoot blades like those of the culm. **Panicles** 1.5–2.5(–3) cm long, 0.7–1.1 cm wide, erect, contracted to loosely contracted, mostly included in the foliage, congested to moderately congested, with 10–25 spikelets, proximal internode 0.4–0.7 cm long; rachis with 2–6 branches per node; primary branches sub-erect to ascending, stout, more or less terete, moderately densely stiff scabrous all around; lateral pedicels 1/4–1/2 the spikelet length, smooth or sparsely to moderately scabrous, prickles fine, sometimes sub-ciliolate; longest branches 0.8–1.5 cm, with up to 6 spikelets in the distal 1/2. **Spikelets** (3–)3.5–6(–5.5) mm long, 2–3 × as long as wide, elliptical in side view, to cunniate at maturity, laterally compressed, not bulbiferous, green, sub-lustrous; florets 2, lower hermaphroditic, upper often pistillate; rachilla internodes terete, 0.2–0.3 mm long, smooth, glabrous; glumes broadly lanceolate, central portion green, margins broadly creamy-white scarious, equal, both exceeding the florets, chartaceous on back, smooth, edges obscurely scaberulous, apex firm, acute, sometimes a bit anthocyanic; both glumes (2.5–)3–6(–5.5) mm long, 3-veined; calluses indistinct, glabrous; lemmas 2.3–2.8 mm long, 3-veined, elliptic to oval, pale green, not lustrous, strongly keeled, keel moderately to densely, and upper 2/3 surfaces lightly scaberulous, intermediate veins absent, margins and apex narrowly and briefly scarious-hyaline, edges moderately to sparsely scaberulous; apex obtuse to acute, sometimes denticulate in the upper margin; palea keels finely scabrous, between veins smooth or lightly scaberulous. **Flowers**; lodicules 0.5 mm long, narrowly lanceolate, unlobed; anthers 0.5–1.1 mm long, those of the upper flower vestigial or sometimes poorly formed but not much reduced. **Caryopses** 1.3 mm long, elliptical in side-view, laterally compressed, slightly sulcate, hilum oval about 0.2 mm long, grain adherent to the palea. 2*n* = unknown.

### Distribution.

The species occurs in Bolivia and Peru, and in Mexico it is known from the states of Mexico, Puebla, San Luis Potosí, Tlaxcala, and Veracruz.

### Ecology.

This species is found on fairly well drained alpine volcanic slopes between 3800–4550 m. Flowering August to September.

### Specimens examined.

Mexico. **San Luis Potosí:** Virlet d’Aoust 1434 (US fragm.). **Puebla:** Ixtaccihuatl, Oct 1905, C.A.Purpus 1633 (US). Ixtaccihuatl S flank, in circ E of the “portal" N of La Amacuilecatl (Los Pies), 4400−4450 m, 19.15426°N, 98.63072°W, 3 Oct 1987, R.J.Soreng 3317 & N.Soreng (US). falda SO de Ixtaccihuatl, 3800 m, 1 May 1952, E.Matuda 26104 (US). Municipio de San Nicoas de los Ranchos La Joya, Volcán Ixtaccihuatl, 7 km a N de al carretara pavimento a Amecameca, 3920–4000 m, 28 Oct 1976, S.D.Koch 76236 (US). north side of Popocatepetl, 11 Sep 1957, J.H.Beaman 1732 (US). **Mexico:** Tlaloc, near summit, 4100–4140 m, 22 Aug 1958, J.H.Beaman 2329 (US). Nevado de Toluca, 1865–1866, Hann s.n. (P, US fragm. ex P-STEUD). Nevado de Toluca, 13500 ft [4115 m], (19°06'00"N, 99°45'36"W), bottom of the crater, 01 Sep 1892, C.G.Pringle 4222 (MO not seen, US, US, US); ditto, summit, 4545 m., 09 Oct 1986, P.M.Peterson 04655 & C.R.Annable (US); ditto, on south rim of crater, 8 Sep 1957, J.H.Beaman 1692 (US). Laguna de Sol, 13 miles east of Mex. highway 3 on road to Nevado de Toluca., 4000 m., 08 Oct 1986, P.M.Peterson 4646 & C.R.Annable (US). **Tlaxcala:** Malinche, North rim of the crater, 4400–4450 m, 10 Aug 1958, J.H.Beaman 2240 (US). **Veracruz:** Summit of Cofre de Perote, 19°29'42"N, 97°08'55"W, 4140 m, 20 Sep 1997, S.J.Darbyshire 4806 & M.Gonzáles-Ledesma (US).

### Discussion.

First reported from Mexico by Fournier (1886) as *Dissanthelium sclerochloides*, Beetle (1987) accepted *Dissanthelium mathewsii* Ball as the correct name. Soreng (1998) and Refulio-Rodríguez et al. (2012) concluded there were too many intermediates between this and the smaller and shorter spikeleted *Dissanthelium calycina* (J. Presl) Kunth, and therefore, treated the taxon as a subspecies or variety. *Poa calycina* var. *calycina* and var. *mathewsii* are known from Bolivia and Peru. Historically, the species was treated within *Dissanthelium*. However, DNA analyses have confirmed that all elements of that genus belong within *Poa* (Refulio-Rodríguez et al. 2012). All but one species formerly placed in *Dissanthelium* differ from other *Poa* by having spikelets with two florets, lemmas 3-nerved (rare in *Poa*), and glumes that are longer than at least the lowest lemma (a feature that occurs infrequently in other groups of *Poa*). *Poa calycina* belongs to *Poa* sect. *Dissanthelium* (Trin.) Refulio (see also *Poa thomasii* below).

**Figure 5.**
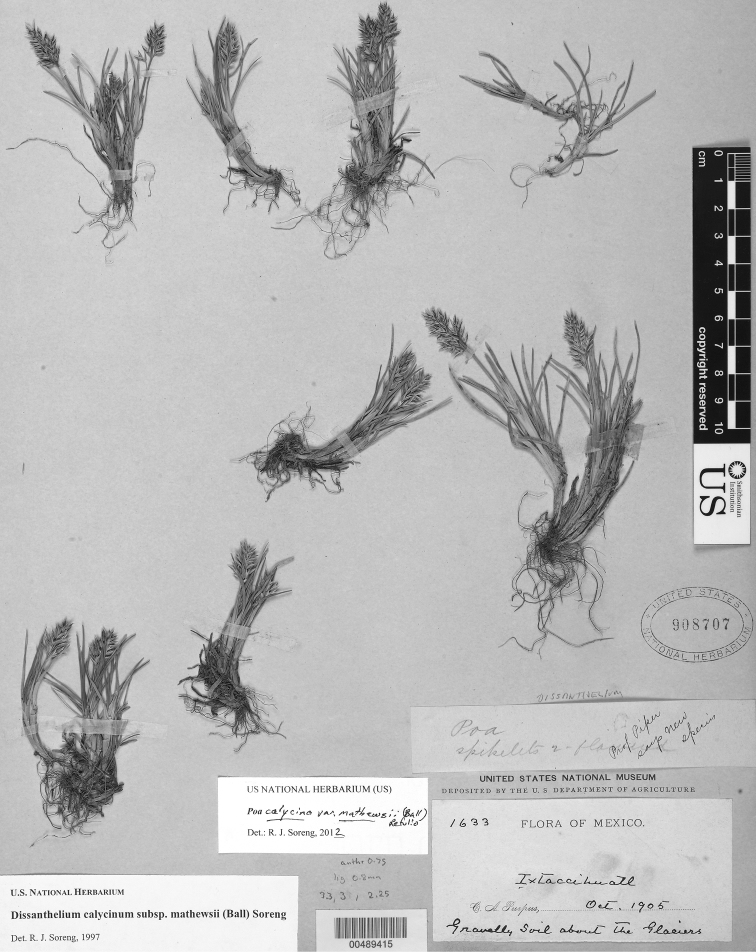
*Poa calycina* var. *mathewsii* (Ball) Refulio. Photo of *Purpus 1633*.

## 6. Poa chamaeclinos

Pilg., Bot. Jahrb. Syst. 37: 379. 1906.

http://species-id.net/wiki/Poa_chamaeclinos

Figs 6 F–I 7

### Type:

Peru, in andibus elevatis supra Lima ad 4500 m, Mar 1904, *A. Weberbauer 5118* (lectotype: USM!, designated by Anton and Negritto 1997: 237; isolectotypes: BAA-2510!, US-89685! fragm. ex B).

### Description.

Pistillate. **Perennials**; mat forming, mats dense (to 20 cm across), low (mostly 1.5–3 cm tall), green; tillers intravaginal (each subtended by a single elongated, tubular prophyll), without cataphyllous shoots. **Culms** 1–3.5(–5) cm tall, erect or arching, leafy, terete or weakly compressed, smooth; nodes 0–1, not exerted, deeply buried in basal tuft. **Leaves** mostly basal; leaf sheaths laterally slightly compressed, indistinctly keeled, basal ones with cross-veins, smooth or with sparse hooks, glabrous; butt sheaths becoming papery to somewhat fibrous, smooth, glabrous; flag leaf sheaths 0.8–2 cm long, margins fused ca. 50–60% their length, 2–3 × longer than its blade; throats and collars smooth or slightly scabrous, glabrous; ligules 0.5–3.2 mm long, decurrent, scarious, milky-white, abaxially smooth or scaberulous, upper margin entire or denticulate, apices obtuse to acute, sterile shoot ligules equaling or shorter than those of the upper culm leaves; blades 1–2.2 cm long, 1–1.5 mm wide (expanded), folded, often with involute margins, slightly thick, soft, abaxially smooth, veins slightly expressed, margins proximally scabrous, distally smooth, adaxially smooth or lightly scaberulous, with two rows of buliform cells, but otherwise level, apices abruptly prow-tipped; flag leaf blades like the others; sterile shoot blades like those of the culm. **Panicles** 0.8–1.5 cm long, 3–7 mm wide, erect, densely contracted, narrowly elliptic to ovoid, erect, slightly secund, included in the leaves or eventually exerted by up to 3(–4) cm, congested, with 5–20 spikelets, peduncle smooth; rachis with 1–2 branches per node; primary branches erect, appressed, stiff, slightly angled, smooth or distally slightly to moderately scaberulous on the angles; lateral pedicels less than 1/4 their spikelet in length, scabrous angled, hooks moderate in size to sub-ciliate; longest branches 0.3–0.4 cm, with up to 4 spikelets, flowered from near the base. **Spikelets** 4–4.5 mm long, 1.5–2.5 mm wide; 2–3 × as long as wide, ovate, laterally compressed, not bulbiferous, slightly lustrous, two toned; florets (1–)2, pistillate; rachilla internodes terete, mostly 0.2–0.5 mm long, smooth, glabrous; glumes obovate to sub-flabellate, herbaceous and pale green below, scarious bronzy and sometimes anthocyanic in margins and apex, veins distinct, equal to subequal, distinctly keeled, sometimes a bit asymmetrical, smooth, margins broadly scarious-hyaline, edges entire or dentate, smooth, apices dentate to shortly lacerate; lower glumes 2.9–3.3 mm long, 3-veined; upper glumes 3.2–3.3 mm long, 3-veined; calluses glabrous; lemmas 3.5–4.2(–4.5) mm long, 5-veined, obovate to sub-flabellate, thinly herbaceous and pale green below, strongly keeled, surfaces smooth, glabrous intermediate veins distinct, upper margins broadly bronzy-anthocyanic, apices obtuse, sometimes lacerated or dentate; paleas glabrous, keels smooth, closely spaced, between keels smooth (or with a few hooks), Flowers; lodicules 0.4–0.5 mm long, broadly lanceolate, apex rounded; anthers vestigial, 0.1–0.2 mm long. **Caryopses** 1.8 mm long, elliptical in side-view, cylindrical in cross-section, sulcus indistinct, hilum 0.4 mm long, oval, grain free from the palea, stigma densely plumose. 2*n* = unknown.

### Distribution.

The species occurs in Bolivia, Peru, and Mexico. In Mexico it is restricted to Volcán Ixtaccihuatl, and possibly to the state of Puebla.

### Ecology.

The species occurs as isolated mats in wet meadows and on gentle slopes between 4300−4450 m. The spikelets of this species are strictly pistillate and seed is produced apomicticly. Flowering September to October.

### Specimens examined.

Mexico. **Mexico or Puebla:** Ixtaccihuatl, Jan 1909, C.A.Purpus 3772 (US-924996). **Puebla:** Ixtaccihuatl, south side of mountain, ca. ½ km NE of summit of Pies, 4300−4350 m, 16 Sep 1958, J.H.Beaman 2555 (MEXU-58016, MSC, US-2381604, TEX, WIS); ditto, in the circ E of the “portal" N of La Amacuilecatl (Los Pies), 19.1507°N; 98.6294°W, 4400−4450 m, 3 Oct 1987, R.J.Soreng 3315 & N.Soreng (US, Soreng 1990, cpDNA voucher).

### Discussion.

In Mexico, the *Purpus 3772* collection long passed under the name *Poa villaroelii* Phil. (Hitchcock 1913, Espejo Serna et al. 2000, Dávila Aranda et al. 2006). However, the first author has studied *Poa villaroelii* in Chile and the herbarium (including types), and concludes the Mexican specimens are not applicable (Soreng et al. 2003, Soreng and Peterson 2007). *Poa chamaeclinos* has strictly pistillate spikelets, reproduces apomicticly, and has broad, bronze-colored, scareous-hyaline lemma apices, whereas *Poa villaroelii* (now treated as *Poa acinaciphylla* E. Desv.) has perfect flowers (anthers 2.2−2.8 mm long) with narrow, whitish, scareous-hyaline apices, and is a much more robust species. Clayton et al. (2006 onwards; accessed Dec. 2011) mistakenly accepted *Poa acinaciphylla* in Mexico and South America and *Poa chamaeclinos* only in South America.

A further taxonomic problem arises when trying to reliably distinguish *Poa chamaeclinos* from *Poa perligulata* Pilg., a closely related species of South America that also has pistillate spikelets and reproduces apomicticly. Negritto and Anton (2000) argued that *Poa chamaeclinos* differs by lacking rhizomes, and by having short ligules [0.3−1 (–3) mm long] that have truncate apices with entire, erose or denticulate margins versus longer ligules [1.5−3 (–6) mm long] with acute apices and entire margins in *Poa perligulata*. The Mexican plants lack rhizomes and the vegetative branching seems to be entirely intravaginal (in *Poa perligulata* some vegetative extravaginal branching is always present). The ligules in the Mexican specimens are ca. 3 mm long, albeit with the denticulate margins. The ligule length overlaps between these taxa and the margin character is not considered reliable for separating the species. Soreng (1990) initially accepted the Mexican plants as *Poa chamaeclinos*. However, Soreng et al. (2003a) decided to treat the Mexican plants as *Poa perligulata* since the *Poa chamaeclinos* isolectotype at US seemed to be a plant of drier habitat with shorter ligules, stiffer leaves, and slightly scabrous lemmas that are slightly firmer and scareous near the apex. The lectotype of *Poa chamaeclinos* at USM, illustrated by Anton and Negritto (1997, Fig. 5) seems to fit the Mexican material, whereas their lectotype for *Poa perligulata* does not (Negritto and Anton 2000). They also indicate that the distinction between the two taxa is difficult and needs further work (Negritto and Anton 2000). If the lack of rhizomes is a good diagnostic character for *Poa chamaeclinos*, then the Mexican material should be referred to that taxon. Beaman (accompanying notes with the US specimens) considered naming the Mexican plants as a new species with the epithet “cordylina". Unlike *Poa gymnantha*, which does not tolerate poorly drained soils, *Poa chamaeclinos* and *Poa perligulata* grow in perennially wet or “waterlogged" habitats, often with densely-interwoven vegetation. On Ixtaccihuatl, RJS recalls a large population to have occurred over much of the wet floor of the southeastern glacial circ, forming discrete, low mats to about 20 cm in diameter.

**Figure 6.**
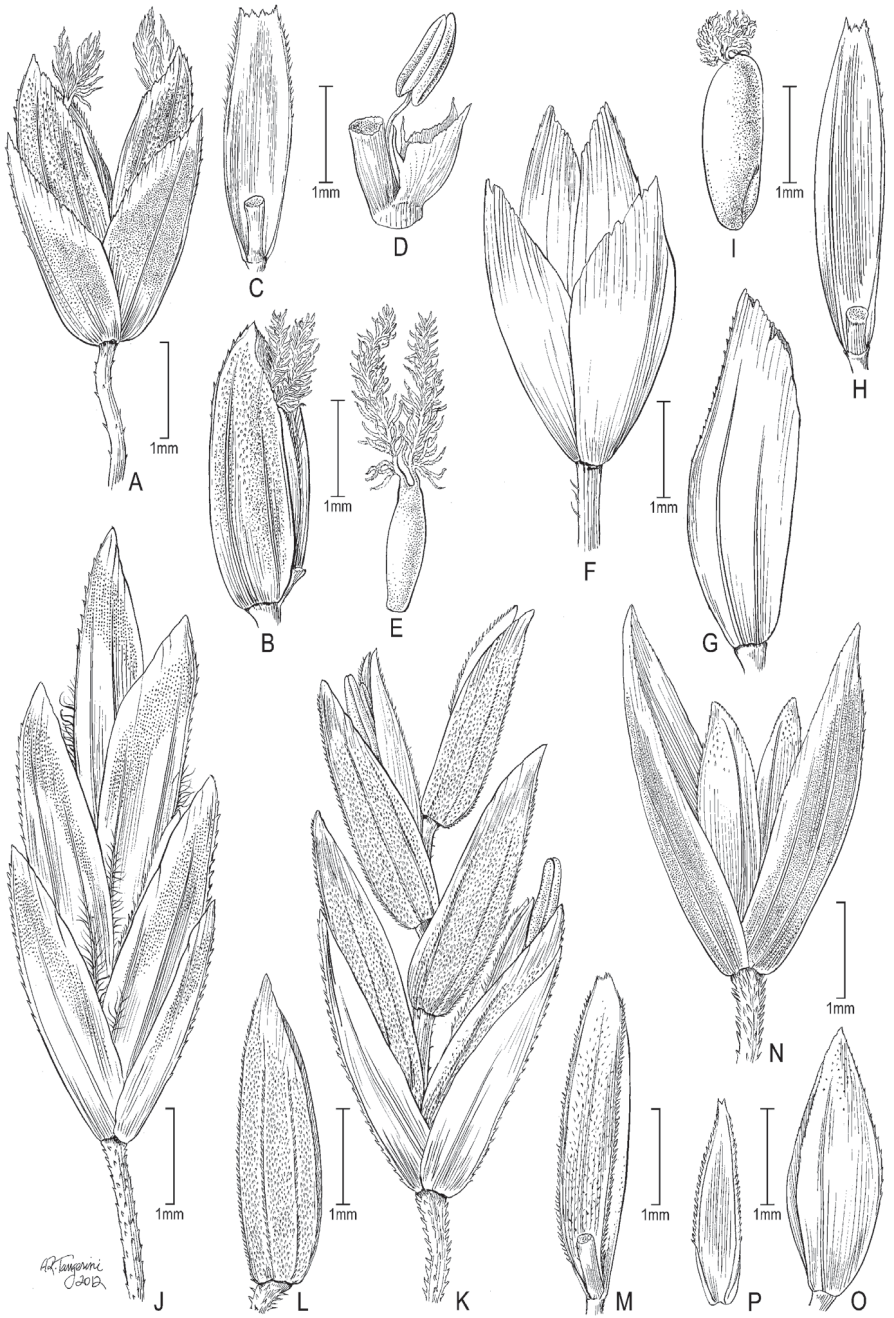
**A–E** *Poa gymnantha* Pilg. **A** spikelet **B** lemma and palea **C** palea **D** staminode and lodicules (pistillate-flower) **E** pistil (pistillate-flower) **F–I** *Poa chamaeclinos* Pilg. **F** spikelet **G** floret **H** palea **I **pistil (pistillate-flower) **J** *Poa palmeri* Soreng & P.M.Peterson **J** spikelet **K–M** *Poa strictiramea* Hitchc. **K** spikelet **L** floret **M** palea **N–P** *Poa calycina* var. *mathewsii* (Ball) Refulio **N** spikelet **O** floret **P** palea. **A–E** drawn from *Peterson 12863 et al.* from Peru **F–I** drawn from *Soreng 3315 & Soreng*; **J** drawn from *Peterson 18790 & Valdés-Reyna* **K–M** drawn from *Soreng 3204 & Spellenberg* **N–P** drawn from *Beaman 1732*.

**Figure 7.**
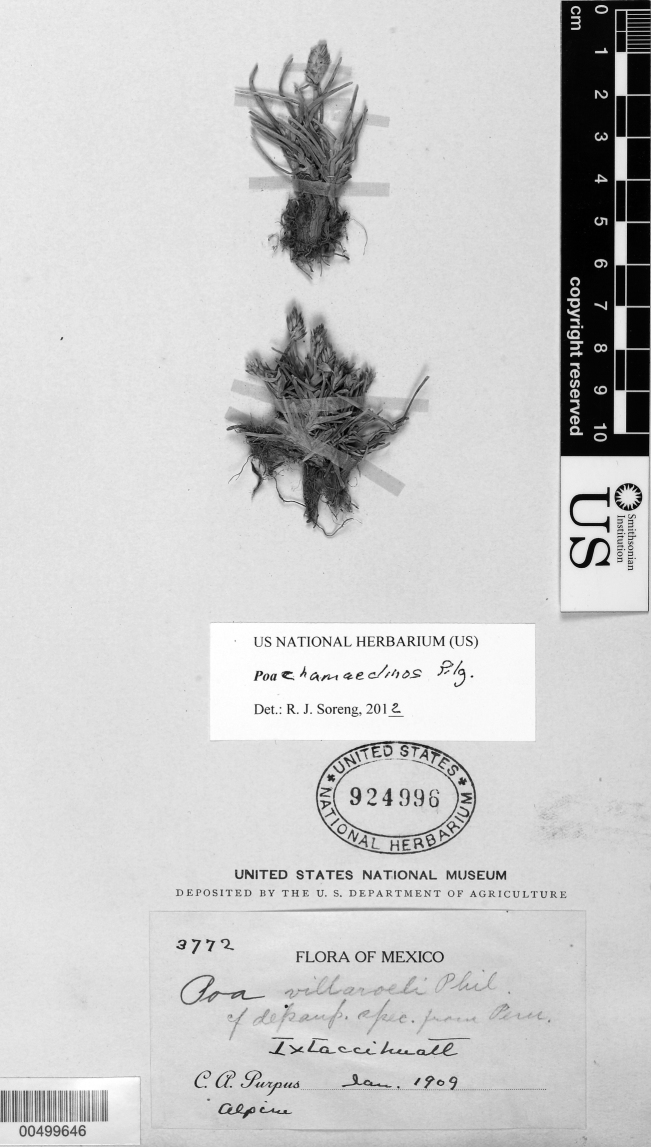
*Poa chamaeclinos* Pilg. Photo of *Purpus 3772*.

## 7. Poa compressa

L., Sp. Pl. 1: 69. 1753.

http://species-id.net/wiki/Poa_compressa

Fig. 1 F–M

### Type:

Habitat in Europae and Americae septentrionalis, (lectotype: LINN-87.41!, designated by Soreng 2000: 255).

### Description.

Hermaphroditic. **Perennials**; extensively rhizomatous, shoots solitary, green or bluish-grey-green; tillers extravaginal (basally cataphyllous), with lateral and downward tending, cataphyllous shoots. **Culms** 15–60 cm tall, erect, bases usually geniculate, wiry, leafy, strongly compressed, smooth; nodes strongly compressed, 3–4 nodes usually exerted. **Leaf** sheaths distinctly compressed, minutely rough; butt sheaths papery, smooth, glabrous; flag leaf sheaths ca. 2–6 cm long, margins fused 10–20% the length, subequal to its blade; throats and collars smooth or slightly scabrous, glabrous; ligules 1–3 mm long, abaxially moderately to densely scabrous, upper margin ciliolate, apices obtuse; blades 1.5–4 mm wide, flat or folded, abaxially smooth, veins slightly expressed, margins scabrous, adaxially lightly scabrous over the veins, apices abruptly prow-tipped; cauline blades subequal; sterile shoot blades like those of the culm. **Panicles** 2–10 cm long, generally 1/6–1/3 as broad as long, erect, contracted or slightly open, linear, lanceoloid to ovoid, often interrupted, sparse to congested, with 15 to 80 spikelets; rachis with mostly 1–3 branches per node; primary branches erect to ascending, or infrequently spreading, fairly strict, 2–3 angled, angles distinctly scabrous (at least in part); lateral pedicels 1/5–2/3 their spikelet in length, scabrous, prickles moderately coarse; longest branches 0.5–3 cm, with 1–15 spikelets. **Spikelets** (2.3–)3.5–7 mm long, laterally compressed; not bulbiferous; grayish, often anthocyanic tinged, not lustrous; florets 3–7, hermaphroditic; rachilla internodes terete, mostly less than 1 mm long, smooth to muriculate; glumes lanceolate, subequal, distinctly keeled, keels scabrous; apices acute; lower glumes ca. 2 mm long, 3-veined; upper glumes ca. 2.1 mm long, 3-veined; calluses glabrous or more often webbed; web distinct, hairs short, woolly, sparse; lemmas 2.3–3.5 mm long, lanceolate, distinctly keeled, keels and marginal veins short villous proximally, between veins smooth, glabrous, intermediate veins obscure, margins narrowly scarious-hyaline, edges smooth or with sparsely scaberulous, apices obtuse to acute; paleas densely scabrous over the keels, between keels smooth. **Flowers** chasmogamous; lodicules ca. 0.6 mm long, lanceolate, with a subequal lateral lobe in the upper 2/3; anthers 1.3–1.8 mm long. **Caryopses** 1.4–1.5 mm long, elliptical in side-view, subtrigonous to subcylindrical in cross-section, brown, shallowly sulcate, hilum 0.2 mm long, oval, grain adherent to the palea. 2*n* = 35, 42, 49, 50, 56.

### Distribution.

This species is circumboreal in distribution and in North America it occurs in Canada, USA, and Mexico (Coahuila).

### Ecology.

This strongly rhizomatous, ruderal species occurs in mesic, cool temperate, semi-shaded to open habitats in seasonally soggy soils, sands to clays, both derived from calcareous and igneous substrates. Once established, this species readily spreads by rhizomes.

### Specimens examined.

Mexico. **Coahuila:** 51.6 km SE of Saltillo and 13 km SE of Jamé on road to Sierra La Viga, 3240 m, 26 Sep 1990, P.M.Peterson 10045, C.R.Annable & J.Valdes-Reyna (US).

### Discussion.

This species has been introduced into the New World for soil stabilization and it is presumed also to be native in northern USA and southern Canada (Beal 1896), but has only recently been collected in Mexico. *Poa compressa* is presumed to be an introduction in Mexico, although the second author has observed this species in other northern Mexico states but did not obtain vouchers because it was not flowering.

## 8. Poa fendleriana

(Steud.) Vasey, U.S.D.A. Div. Bot. Bull. 13(2): t. 74. 1893.

http://species-id.net/wiki/Poa_fendleriana

Fig. 8

Eragrostis fendleriana Steud. Syn. Pl. Glumac. 1: 278. 1854. *Uralepis poaeoides* Buckley, Proc. Acad. Nat. Sci. Philadelphia 14: 94. 1862. *Atropis fendleriana* (Steud.) Beal, Grass. N. Amer. 2: 576. 1896. *Panicularia fendleriana* (Steud.) Kuntze, Revis. Gen. Pl. 2: 782. 1891. *Puccinellia fendleriana* (Steud.) Ponert, Feddes Repert. 84(9–10): 739. 1974. Type: USA, New Mexico (probably in Santa Fe Canyon above Santa Fe), 1847, *Fendler 932* (holotype: P-STEUD; isotypes: CAEN, GH!, NY!, US-2891469! specimen with fragm. ex CAEN, fragm. ex NY, and fragm. ex P-STEUD).

### Description.

Dioecious (sometimes strictly pistillate). **Perennials**; tufted, sometimes noticeably sub-rhizomatous to long-rhizomatous, tufts dense to a bit loose, generally of medium girth and height (mostly 10–25 cm tall), pale green to bluish-green; tillers mainly intravaginal (each subtended by a single elongated, 2-keeled, longitudinally split prophyll), usually some extravaginal (basally cataphyllous), with lateral or downward tending, cataphyllous shoots, sterile shoots more numerous than flowering shoots. **Culms** 15–70 cm tall, erect or bases decumbent, slender or sometimes stout, blades strongly reduced upward, terete or weakly compressed, smooth or slightly scabrous above; nodes terete, 0–1 exerted. **Leaves** mostly basal; leaf sheaths terete, smooth or scabrous, glabrous or occasionally retrorsely puberulent; bases of butt sheaths thick papery, smooth, glabrous, sub-lustrous; flag leaf sheaths (6–)10–20 or more cm long, margins fused ca. 33% the length, usually more than (5–)9 × long as its blade; collars smooth or scabrous, glabrous or hispidulous; ligules 0.2–18 mm long, decurrent or not, abaxially smooth or scabrous, upper margin ciliolate or glabrous, apices truncate to acuminate; blades of cauline leaves (0.5–)1–3(–4) mm wide, folded, usually involute on the margins, moderately thick and firm, infrequently moderately thin, abaxially smooth or infrequently scabrous, narrowly prow-tipped; cauline blades steeply reduced in length distally along the culm, flag leaf blades usually absent or very reduced, or some up to 1(–3) cm long; sterile shoot blades usually moderately to densely scabrous or hispidulous on and between the veins, or infrequently nearly smooth and glabrous. **Panicles** 2–12(–30) cm long, erect, contracted (open in anthesis), narrowly lanceoloid to ovoid, congested (except in flowering), with (15–)25–80 (more than 100) spikelets; rachis with 1–2 branches per node; primary branches erect, fairly stout, terete to weakly angled, smooth or scabrous, prickles not confined to angles; lateral pedicels 1/5–1/4 the spikelet in length, sparsely to fairly densely scabrous, prickles fine to fairly coarse; longest branches 1–8 cm, with 3–15(–25) spikelets. **Spikelets** (3–)4–8(–12) mm long, to 3 × long as wide, sub-lustrous, broadly lanceolate to ovate, laterally compressed, not sexually dimorphic; not bulbiferous; florets 2–7(–13), pistillate or staminate; rachilla internodes terete, usually 0.8–1.3 mm long, smooth, glabrous, sparsely hispidulous, or sparsely softly puberulent to short villous; glumes lanceolate, distinctly keeled, keel smooth or sparingly scabrous, margins fairly broadly scarious, edges smooth or lightly scabrous; apices obtuse to acute; lower glumes distinctly shorter than the lowest lemma, 1–3-veined; upper glumes lanceolate, slightly shorter than lowest lemma, 3-veined; calluses glabrous; lemmas (3–)4.5–6 mm long, lanceolate, pallid green, commonly anthocyanic distinctly keeled, keels and marginal veins short to long villous or softly puberulent, or glabrous, intermediate veins short villous to softly puberulent or glabrous, between veins softly puberulent or glabrous, smooth or sparsely finely scabrous, intermediate veins obscure to moderately prominent, margins narrowly scarious to hyaline, edges smooth or lightly scabrous, apices acute to obtuse, sometimes retusely notched (to 0.25 mm deep); palea keels coarsely scabrous, medially sometimes softly puberulent or long villous, between keels glabrous or infrequently puberulent. **Flowers** chasmogamous; lodicules of pistillate plants 0.6–0.85 mm long, broadly lanceolate to ovate, with or without a brief lateral lobe from below the middle (lodicules rudimentary in staminate plants); anthers 2–3.2 mm long, or all vestigial and 0.1–0.2 mm long. **Caryopses** 2–2.5 mm long, elliptical in side-view, subtrigonous in cross-section, honey-brown, sulcus broad and shallow, hilum 0.25 mm long, round, grain adherent to the palea.

### Distribution.

The species is widespread in North America and occurs in southwestern and south central Canada, western USA, and northern Mexico in Baja California, Chihuahua, Coahuila, and Sonora.

### Ecology.

This dioecious, weakly rhizomatous species occurs in the mountains on open forested slopes derived from calcareous and igneous substrates. It is sometimes strictly pistillate and apomictic. Flowering principally in the spring.

### Discussion.

This species provides good springtime forage where it is abundant and all three subspecies occur in Northern Mexico (Soreng and Van Devender 1989; Soreng et al. 2003a). The sex of specimens is indicated here for purposes of estimating the breeding system (geographical and ecological extent of sexual versus asexual reproduction), along with numbers of individuals of each sex where populations were evaluated. Where only or predominately pistillate plants are found, the species usually reproduces apomicticly by seed. No pollen is required to stimulate seed development in apomictic plants of this species (i.e., they are not pseudogamous). Sexual reproduction predominates where staminate plants are found and relatively numerous. Seed is commonly set in 20% or more of flowers in the apomictic populations and usually less than 10% in pistillate plants in sexual populations. Sex-expression is apparently stable in the species of *Poa* sect. *Madropoa*, to which *Poa fendleriana* belongs. The first author grew samples of the species in a common garden over several years and found that plants did not change sex. Individuals of *Poa fendleriana* occasionally have a few perfect-flowered spikelets, and a few populations containing these unusual plants have been found in *Poa fendleriana* subsp. *fendleriana* in New Mexico and Colorado. *Poa fendleriana* subsp. *albescens* is a tetraploid whereas the other two subspecies are principally octoploid (Soreng 2005).

A specimen from 20 mi N of Durango, Oct 7 1955, *B.Emery 334A* (TEX), has one flowering culm of *Poa fendleriana* mixed in with vegetative parts of *Trachypogon*. The flowers are pistillate and the lemmas are glabrous to sparsely pubescent on the keel; so it appears intermediate between *Poa fendleriana* subsp. *albescens* and *Poa fendleriana* subsp. *fendleriana*. However, the habitat seems wrong (low spots in grassland of mesquite, *Opuntia*, and short grasses), making us wonder if the origin of the one culm stems from a collection sorting error.

**Figure 8.**
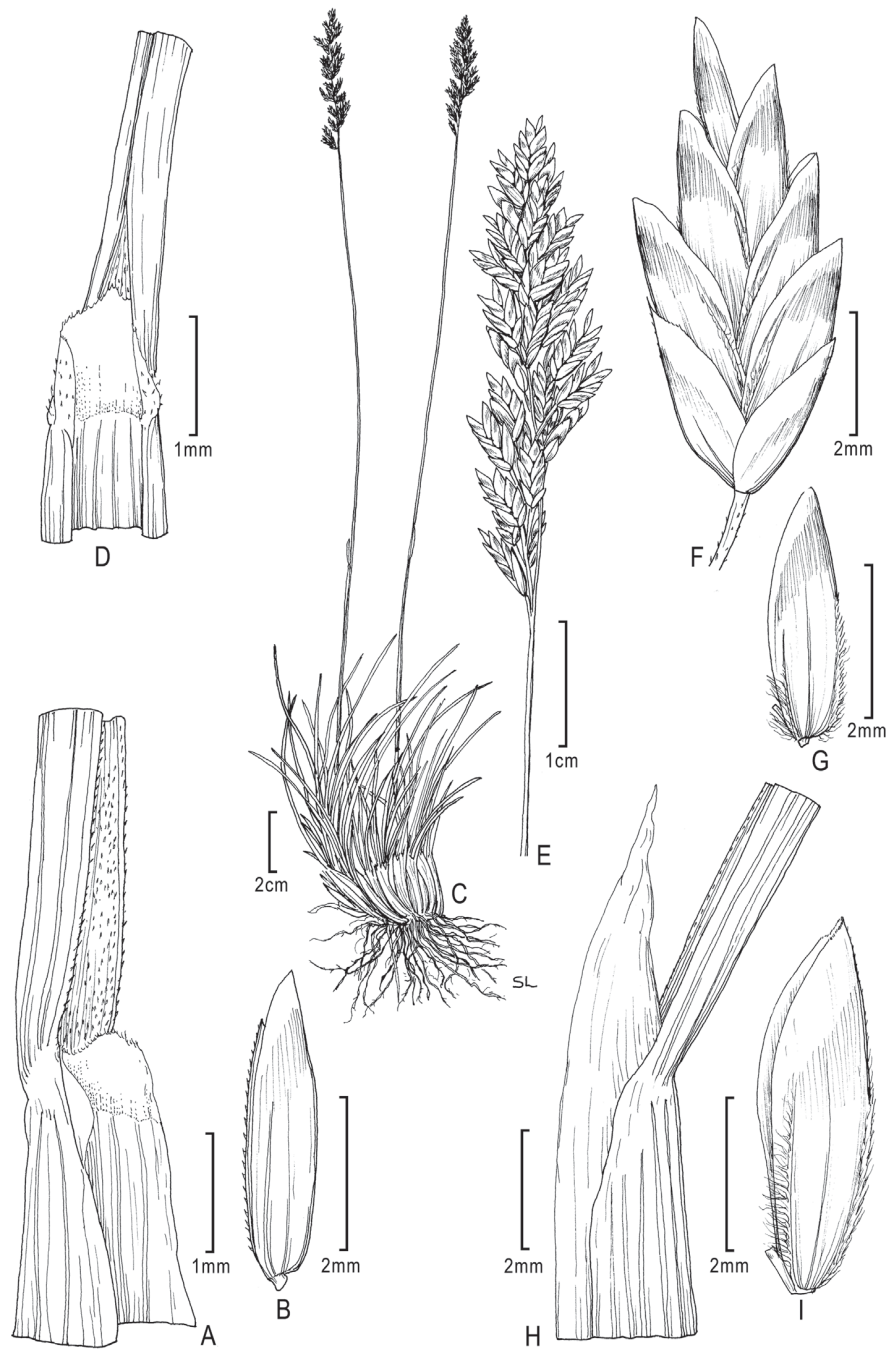
*Poa fendleriana* (Steud.) Vasey. **A, B** *Poa fendleriana* subsp. *albescens* (Hitchc.) Soreng **A** ligule **B** floret **C–G** subsp. *fendleriana***C** habit **D** inflorescence **E** ligule **F** spikelet **G** floret **H, I** subsp. *longiligula* (Scribn. & T.A.Williams) Soreng **H** ligule **I** floret. Drawings from Soreng (2007).

### Key to the subspecies of *Poa fendleriana*

**Table:** 

1	Lemmas glabrous; plants from the Sierra Madre Occidental	8a. *Poa fendleriana* subsp. *albescen*s
–	Lemmas pubescent on the keel and marginal nerves	2
2	Ligules of upper culm leaves 0.2−1(–1.5) mm long, truncate to rounded, upper margin minutely ciliate fringed; collar margins usually distinctly scabrous; plants from the range of the species; Baja California, Chihuahua, Sonora, and Coahuila	8b. *Poa fendleriana* subsp. *fendlerian*a
–	Ligules of upper culm leaves (1.5–)1.8–11 mm long, obtuse to acuminate, upper margin without a ciliate fringe; collar margins usually smooth or sparingly scabrous; plants from Baja California	8c. *Poa fendleriana* subsp. *longiligul*a

## 8a. Poa fendleriana albescens

(Hitchc.) Soreng, Great Basin Naturalist 45(3): 407 1985.

http://species-id.net/wiki/Poa_fendleriana_albescens

Fig. 8 A, B

Poa albescens Hitchc. Contr. U.S. Natl. Herb. 17(3): 375 1913. Type: Mexico, Chihuahua, at Miñaca, 1 Apr 1908, *J.N.Rose 11648* (holotype: US-454361!♀).
Poa griffithsii Hitchc. Contr. U.S. Natl. Herb. 17(3): 375 1913. Type: Mexico, Sonora, Cananea, 7−8 Jul 1903, *D.Griffiths 4865* (holotype: US-691228!♀; isotype: US-3063989!♀).

### Description.

**Leaf** collars often scabrous or hispidulous near the throat; ligules of middle cauline leaves 0.2–1.5 mm long, not decurrent, abaxially smooth or scabrous, upper margin scabrous or ciliolate or glabrous, apices truncate; sterile shoot blades frequently glabrous adaxially. **Spikelet** rachilla internodes smooth, glabrous; lemma keels and marginal veins smooth or sparingly scabrous, glabrous or sparsely short villous to softly puberulent; palea keels glabrous, between keels glabrous. **Lodicules** 0.6–0.7 mm long, broadly lanceolate to ovate, with or without a brief lateral lobe from below the middle. 2*n* = 28, 28+II.

### Distribution.

The subspecies is endemic to southeastern Arizona, southwestern New Mexico, USA, and the northern Sierra Madre Occidental of Mexico (Chihuahua, Sonora).

### Ecology.

The subspecies is found in canyons and rocky slopes in forests and is associated with: *Cupressus*, *Juniperus*, *Pinus arizonica* Engelm., *Poa discolor* D.K. Bailey & Hawksw., *Poa engelmannii* Carrière, *Poa leiophylla* Schiede ex Schltdl. & Cham., *Poa strobiformis* Engelm., *Populus tremuloides*, *Quercus hypoleucoides* A. Camus, *Quercus rugosa* Née, *Quercus sideroxyla* Bonpl., *Abies*, *Pseudotsuga*, and *Picea chihuahuana* Martínez. The subspecies ranges in elevation from 1380–2850 m, and is primarily restricted to regions with summer monsoons and occasional winter snows. Flowering in spring.

### Specimens examined.

Mexico. **Chihuahua:** Arroyo del Gato, 1 mi W of Talayotes, 27°55'N, 107°49' W, 17 mi SW of San Juanito, 7626 ft [2325 m], 29 Apr 1985, R.J.Soreng 2615, R.W.Spellenberg & R.Corrales (US, population sample: 4♀ & 3♂). Basasiachic, in deep barranca below ca. 240 m waterfall, ca. 6500 ft [1890 m], 28°1'N, 108°15'W, 27 Apr 1985, R.J.Soreng 2606, R.W.Spellenberg & R.Corrales (US, population sample: 14♀ & 4♂). 10 mi SE of Basasiachic on road to San Juanito, 8 mi SE of junction with Yecora–Tomochic road, ca. 8900 ft [2715 m], 28 Apr 1985, R.J.Soreng 2609a, R.W.Spellenberg & R.Corrales (US, population sample: 25♀, 19♂). Municipio Bocoyana, S of San Ignacio Arareco, S of Creel air strip, [27.7°N, 107.7°W], on steep north facing rocky cliffs, ca. 7400 ft [2255 m], 20 Jul 1972, R.Bye Jr. 2404 (TAES♂); ditto, along Rio Oteros, west of Creel, R.A. Bye 3673 (MEXU, TEX ♀). Miñaca, vic. of, [107.35°N, 28.45°W], 1 Apr 1908, J.N.Rose 11648 (US-454361♀). Creel air strip 3 mi due S of Creel, 27°43'N, 107°45'W, 7800 ft [2380 m], 15 Apr 1984, R.J.Soreng 2309 & Spellenberg (NMC, US, sexual population: ♂ & ♀; 2*n* = 28; Soreng 1990, cpDNA voucher). 5 km SSW of San Juanito, El Rialito, [107.6°N, 27.9°W], 2400 m, 13 May 1974, W.G.Spaulding s.n., P.S.Martin & P.M.Wiseman (ARIZ ♂ & ♀). Rancho Blanco, 28.2°N, 107.6°W, 19 mi N of San Juanito toward La Junta, ca. 6900 ft [2105 m], 29 Apr 1985, R.J.Soreng 2620, R.W.Spellenberg & R.Corrales (US, population sample: 5♀ with high seed, 0♂). 4 mi N of Rancho Blanco, 24 mi N of San Juanito, ca. 6800 ft [2075 m], 29 Apr 1985, R.J.Soreng 2623, R.W.Spellenberg & R.Corrales (US, population sample: 9♀ with much seed, 0♂). Sierra Las Manzanas, 2 km SW of Tosanachic on road to Agua Caliente, ca. 53 km due W of Ciudad Guerrero, 28.30'N, 108°05W, 6396 ft [1950 m], 13 Apr 1984, R.J.Soreng 2305 & R.W.Spellenberg (NMC, US, population sample: 44♀, 34♂; Soreng 1990, cpDNA voucher). Rio Oteros origin above Arroyo El Ranchito, 27°57.5'N, 107°45'W, 11 road mi SW of San Juanito, 7954 ft [2425 m], 29 Apr 1985, R.J.Soreng 2618, R.W.Spellenberg & R.Corrales (US, population sample: 26♀, 9♂). Tomachic, 6.7 mi E and 5 mi W of Cieneguita, on road to Cuauhtemoc, 30 km SW of Ciudad Guerrero, 28°20'N, 107°43'W, 7400 ft [2255 m], 14 Apr 1984, R.J.Soreng 2307 & R.W.Spellenberg (NMC ♀, US ♀, population all pistillate, apomictic, lemmas all glabrous, 2*n* = 28). crest of pass between Yepomera and Babicora, [29.2°N, 107.9°W], 8 May 1959, D.S.Correll & I.M.Johnston 21635-a (LL♀, very sparsely pubescent to glabrous, intermediate, US♀). between Yepomera and Babicora, [29.2°N, 107.9°W], 8 May 1959, D.S.Correll & I.M.Johnston 21626 (LL, ♀ sparsely pubescent to glabrous, intermediate, US). 9 mi SE of Yoquivo on Basasiachic–San Juanito road, 28.0311°N, 107.9234°W, ca. 7900 ft [2410 m], 28 Apr 1985, R.J.Soreng 2610, R.W.Spellenberg & R.Corrales (US, population sample: 9♀, 10♂). Tal des Rios Tecorichic [garbled] Tarahumare, 4 Apr 1906, Endlich 1209 (US ex B). **Sonora:** Cananea, 7–8 Jul 1903, D.Griffiths 4865 (US-691228♀; US-306389♀). ca. 3 mi NW of Cananea, road to microwave station N of Mexico Highway 2, ca. 7000ft [2135 m], 31.036°N, 110.375°W, 19 Mar 1982, R.J.Soreng 1780 & R.W.Spellenberg (NMC, US, population sample: 5♂, 2*n* = 28; Soreng 1990, cpDNA voucher). Santa Cruz, Parry s.n., [Mexican Boundary Survey ] (GH, ♂ very sparse pubescent, ♀ pubescent to sparse pubescent, intermediates; another label on same GH sheet says Munro 165, G.Thurber Herb.). Sierra del Pinito, [30.5°N, 109.5°W], 2500 m, 9 Apr 1977, J.L.Fernandez 3 (ARIZ♀). Sierra Guacomea, Pozo del Santo Nino, Rancho La Alameda, 31°03'22"N, 110°58'06"W, 1472 m, 5 Apr 2005, A.L.Reina-G. 2005-562, T.R.VanDevender & J.Ruiz-C. (ARIZ, US♂); ditto, Rancho La Arboleda, Arroyo El Volteadero, 31°03'10"N, 110°57'04"W, 1385 m, 2 Apr 2005, T.R.VanDevender 2005-493, A.L.Reina-G. & J.Ruiz-C. (ARIZ, US♀).

### Discussion.

This subspecies has glabrous lemmas and short ligules, and is the most common subspecies of *Poa fendleriana* in Mexico. It is often sexually reproducing with dioecious populations and staminate plants are common, but sometimes apomictic individuals with pistillate spikelets are found. Intermediates have sparsely pubescent lemmas and are almost always pistillate. Distribution maps of the subspecies, distribution of staminate and pistillate plants, and a discussion of the breeding system and fossil record is given in Soreng and Van Devender (1989). Hitchcock (1913) suggested this taxon (*Poa albescens*) is allied to *Poa chilensis* Trin. (= *Poa holciformis* J. Presl) from Argentina and Chile, a member of *Poa* sect. *Dioicopoa* E. Desv., but DNA data have shown that is not the case (Gillespie et al. 2009).

## 8b. Poa fendleriana fendleriana

http://species-id.net/wiki/Poa_fendleriana_fendleriana

Fig. 8 C–G

### Description.

**Leaf** collars often scabrous or hispidulous near the throat; ligules of middle cauline leaves 0.2–1.2(–1.5) mm long, not decurrent abaxially scabrous, upper margin usually scabrous or ciliolate, apices truncate to rounded; sterile shoot blades usually scabrous or softly puberulent adaxially. **Spikelet** rachilla usually smooth and glabrous; lemmas long villous on keels and marginal veins, between veins glabrous or infrequently softly puberulent; palea keels and between keels infrequently puberulent. 2*n* = 56.

### Distribution.

The subspecies is found throughout the range of the species, but is rare to absent from much of the westernmost part of the USA range. In Mexico the subspecies is found in Baja California, Chihuahua, Coahuila, and Sonora.

### Ecology.

Where the ranges of the subspecies overlap this subspecies often occurs in slightly drier and more open habitats than *Poa* subsp. *albescens*, and more mesic and cooler habitats than *Poa*. subsp. *longiligula*. In Mexico this subspecies can tolerate some disturbance as most of the plants are apomictic, except in Coahuila. The subspecies is found in canyons and rocky slopes, from upper arid grasslands (margins) and chaparral of with scrubby species of *Quercus*, *Arbutus*, *Juniperus*, *Vaqualina*, and *Cercocarpus* to Hudsonian coniferous forests. Like *Poa fendleriana* subsp. *albescens*, this subspecies is primarily restricted to regions with summer monsoons and some winter snows, and ranges from 1900–2200 m. Flowering in spring.

### Specimens examined.

Mexico. **Baja California:** 4.5 mi S of summit of Cerro 1905, Portezuelo de Jamau, 31°34'N, 115°36'W, 1900 m, 20 Apr 1974, R.Moran 21232 (TAES♀, WYAC♀ intermediate between *Poa fendleriana* subsp. *fendleriana* and *longiligula*, pubescent rachilla, short truncate ligule, probably apomictic). Sierra Juarez, Hansen's Ranch, 21 Jun 1885, C.R.Orcutt 1276a (DS, US♀). **Chihuahua:** Barranca del Cobre, SE of Creel 28 mi, N of Rio Urique crossing, ca. 7000ft [2135 m], ca. 27.507°N, 107.498°W, 14 Apr 1984, R.J.Soreng 2312 & R.W.Spellenberg (US, population sample: 10♀ 9 subsp. *fendleriana*; 1 subsp. *albescens*); ditto, ca. 25 mi SE Creel, ca. 27.534°N, 107.508°W, 2313 (US, population sample: 2♀). N of Basuchil, ca. 10 mi NW of Miñaca, loose crumbling red clay on dry ravine side, near ditch, plateau, arid grassland, 2200 m, 8 May 1929, Y.Mexia 2511 (CAS♀, MO♀). Tomachic, 4.2 mi E on road from La Junta to Yecora, ca. 7000 ft [2135 m], 28.375°N, 107.7865°W, 13 Apr 1984, R.J.Soreng 2306b & R.W.Spellenberg (US, population sample: 24♀ subsp. *fendleriana*, 2*n* = 59; 1 subsp. *albescens*). S of Rancho La Consolacion, canyon in north face of Sierra Rica, 29°12'N, 104°7'W, 1400−2000 m, 3 May 1973, M.C.Johnston, T.L.Wendt & F.Chiang-C. 10776A (LL♀ toward subsp. *albescens*); ditto, 10773C (LL♀); ditto, 10777 (LL♀). **Coahuila:** Sierra del Carmen, south peaks of range, NW side of upper Carboneras Canyon, 28°57'N, 102°34'W, 2100 m, 2 Apr 1974, T.Wendt 125, E.Lott & D.Riskind (TEX♂). Municipio de Ocampo, east side of Sierra del Carmen, 28°53'N, 102°28'W, 5200 ft [1590 m], 8 May 1981, D.H.Riskind 2391 (TEX♀).

**Discussion.** This is the most widespread but least common subspecies of *Poa fendleriana* in Mexico. Staminate plants are rare or absent, except in Coahuila where a staminate plant has been collected.* Poa fendleriana* subsp. *fendleriana* intergrades with *Poa fendleriana* subsp. *albescens* in Chihuahua and in southeast Arizona and southwest New Mexico.

## 8c. Poa fendleriana longiligula

(Scribn. & T.A.Williams) Soreng, Great Basin Naturalist 45(3): 408. 1985.

http://species-id.net/wiki/Poa_fendleriana_longiligula

Fig. 8 H–I

Poa longiligula Scribn. & T.A.Williams, Circ. Div. Agrostol. U.S.D.A. 9: 3. 1899. *Paneion longiligulum* (Scribn. & T.A. Williams) Lunell, Amer. Midl. Naturalist 4: 222. 1915. *Poa fendleriana* var. *longiligula* (Scribn. & T.A.Williams) Gould, Madroño 10(3): 94. 1949. Type: USA, Utah, Washington Co., Silver Reef, gravel, 3500 ft [1070 m], 3 May 1894, *M.E.Jones 5149* (holotype: US-278727!; isotypes: MO!, NY-431282!, OSC!, US-922924!).

### Description.

**Leaf** collars smooth to scabrous near the throat; ligules of middle cauline leaves (1.5–)1.8–18 mm long, decurrent, abaxially smooth or lightly scabrous, upper margin usually smooth, glabrous, apices obtuse to acuminate; sterile shoot blades usually scabrous or softly puberulent adaxially. **Spikelet** rachilla internodes usually sparsely hispidulous or sparsely softly puberulent; lemmas long villous on keels and marginal veins and sometimes intermediate veins, between veins glabrous or softly puberulent (sometimes densely so); palea keels and between keels sometimes puberulent. **Lodicules** 0.85 mm long. 2*n* = 56.

### Distribution.

The subspecies occurs in North America, southwestern Canada, western USA, and in Baja California, Mexico.

### Ecology.

Where their ranges overlap *Poa fendleriana* subsp. *longiligula* is fairly restricted to elevations below *Poa fendleriana* subsp. *fendleriana* but where there is some winter snow. In Mexico this subspecies is strictly pistillate, apomictic, and distributed between 1300–1900 m. Flowering in spring.

### Specimens examined.

Mexico. **Baja California:** Hansen’s Ranch, 21 Jun 1885, C.R.Orcutt 1276 (DS♀, DS♀, US). 63 mi SE of Ensenada, 2–3 mi upstream of Rincon, 4.5 mi NE of Santa Catarina, canyon, 4300 ft [1310 m] 22 Apr 1962, R.E.Broder 772 (DS♀, US♀). 4 1/2 mi S of Portezuelo de Jamau, N of Cerro 1905, ca. 31°34'N, 115°36'W, 1775 m, 20 Apr 1974, R.Moran 21226 (CAS♀, ARIZ♀, TAES♀, US). Sierra Juarez, El Progresso, ca. 32°17'N, 115°56'W, 1450 m, 24 May 1975, R.Moran 22044 (TAES♀); ditto, N slope just below summit of Cerro Jamau, ca. 31°34'N, 115°35.5'W, 1890 m, 23 May 1976, R.Moran 23257 (TAES♀); ditto, in steep north slope of Cerro Taraizo, southernmost peak of range, ca. 31°21.75'N, 115°31'W, 1550 m, R.Moran 23007 (TAES♀, ARIZ♀, US); ditto, vicinity of Rancho La Mora, 32°01'N, 115°47'W, 12 Apr 1987, C.Brey 192 (TAES♀). Rancho El Topo, 2 May 1981, A.A.Beetle & R.Alcaraz M-6649 (ARIZ♀, WYAC♀). Sierra San Pedro Mártir, Cañon del Diablo, 31°00'N, 115°24'W, 1700 m, 6 May 1978, R.Moran 25626 (TAES♀).

### Discussion.

This taxon was accepted as *Poa longiligula* by Espejo Serna et al. (2000). Some plants in Baja California of this subspecies are intermediate to *Poa fendleriana* subsp. *fendleriana*, but in general the longer smoother margined ligules and puberulent rachillas are diagnostic. Where the two taxa occur in the same area *Poa fendleriana* subsp. *longiligula* occurs in more xeric habitats, and *Poa fendleriana* subsp. *fendleriana* is found in higher elevations.

## 9. Poa gymnantha

Pilg., Bot. Jahrb. Syst. 56 (Beibl. 123): 28. 1920.

http://species-id.net/wiki/Poa_gymnantha

Figs 6 A–E 9

### Type:

Peru, 15°50' to 16°00'S, südlich von Sumbay, Eisenbahn Arequipa–Puno, Tola–Heide, 4000 m, Apr 1914, *A*.*Weberbauer 6905* (lectotype: S! designated by Anton and Negritto 1997: 236; isolectotypes: BAA-2555!, MOL!, US-1498091!, US-2947085! specimen & fragm. ex B, USM!).

*Poa ovata* Tovar, Mem. Mus. Hist. Nat. “Javier Prado" 15: 17, t.3A. 1965. Type: Peru, Cuzco, Prov. Quispicanchis, en el Paso de Hualla-hualla, 4700 m, 29 Jan 1943, *C.Vargas 3187* (holotype: US1865932!).

*Poa pseudoaequigluma* Tovar, Bol. Soc. Peruana Bot. 7: 8. 1874.Type: Peru, Ayacucho, Prov. Lucanas, Pampa Galeras, Reserva Nacional de Vicunas, entre Nazca y Puquio, Valle de Cupitay, 4000 m, 4 Apr 1970, *O.Tovar & Franklin 6631* (holotype: USM!; isotypes: CORD!, MO-3812380!, US-2942178!, US-3029235!).

### Description.

Pistillate. **Perennials**; tufted, tufts dense, usually narrow, low (4–6 cm tall), pale green; tillers intravaginal (each subtended by a single elongated, 2-keeled, longitudinally split prophyll), without cataphyllous shoots, sterile shoots more numerous than flowering shoots. **Culms** 4–6 (45) cm tall, erect or arching, leaves mostly basal, terete or weakly compressed, smooth; nodes terete, 0–1, not exerted, deeply buried in basal tuft. **Leaves** mostly basal; leaf sheaths laterally slightly compressed, indistinctly keeled, basal ones with cross-veins, smooth, glabrous; butt sheaths becoming papery to somewhat fibrous, smooth, glabrous; flag leaf sheaths 2–3.5(–10) cm long, margins fused 30–40% their length, ca. 2.5 × longer than its blade; throats and collars smooth or slightly scabrous, glabrous; ligules to 1–0.5(–7) mm long, decurrent, scarious, colorless, abaxially moderately densely scabrous to hirtellous, apex truncate to obtuse, upper margin erose to denticulate, sterile shoot ligules equaling or shorter than those of the upper culm leaves; blades 1.5–3(–12) cm long, 0.6–1.5(–3) mm wide (expanded), folded to involute, slightly thick, slightly firm, margins involute, abaxially smooth, veins not expressed, margins long scabrous for most of the length, adaxially densely scaberulous, with 2 rows of buliform cells, apex slightly prow-tipped; flag leaf blades like the others; sterile shoot blades like those of the culm. **Panicles** 1.5–1.7(–8) cm long, 2–2.5(–1.2) mm wide, erect, tightly contracted, linear, slightly secund, included in the leaves or slightly exerted, congested, with 7–10 (many) spikelets, peduncle smooth, proximal internode 0.4–0.7 cm long; rachis with 1–2(–3) branches per node; primary branches erect, appressed, stout, slightly angled, smooth or distally slightly to moderately scabrous to hirtellous on the angles; lateral pedicels less than 1/2 their spikelet in length, moderately scabrous, prickles fine; longest branches 0.3–0.8 cm (?), with 1 to 2 spikelets (?), flowered from near the base. **Spikelets** 3 mm 6.5 long, 1–1.3(–2.5) mm wide; 2–3 × as long as wide, lanceolate to ovate, laterally compressed, not bulbiferous, slightly lustrous, two toned; florets 1–2(–3), pistillate; rachilla internodes terete, mostly 0.2–0.4 (?) mm long, smooth or scabrous, glabrous; glumes broadly lanceolate, herbaceous and pale green below, scarious bronzy and sometimes anthocyanic in margins and apex, veins distinct, equal to subequal, distinctly keeled, sometimes a bit asymmetrical, subequal to the spikelet, smooth (or scabrous), margins broadly scarious-hyaline, edges entire or dentate, smooth, apices entire; lower glumes 2.5–3(–3.4) mm long, (1–)3-veined; upper glumes 2.7–3(–4.8) mm long, 3-veined; calluses glabrous; lemmas 2.5–3(–5) mm long, 5-veined, (ovate) elliptical (lanceolate), chartaceous green below keeled, surfaces glabrous, proximally smooth, keel and sides distally moderately to densely scabrous (prickle hairs sometimes a bit flexuous) to scaberulous, intermediate veins indistinct, upper margins broadly bronzy-anthocyanic, apex entire, obtuse to acute, paleas glabrous, keels distally scabrous. **Flowers**; lodicules broadly lanceolate, apex acute, with or without a lateral lobe; anthers vestigial, 0.1–0.2(–0.8) mm long. **Caryopses** 1.7–1.8 mm long, elliptical in side-view, subcylindrical in cross-section, light honey-brown, sulcus indistinct, hilum 0.25 mm long, round, grain free from the palea. 2*n* = 70.

### Distribution.

In South America the species occurs Argentina, Bolivia, Chile, and Peru; and is known only from the state of Mexico.

### Ecology.

This species is typically found on well drained slopes, in loam, sandy loam, scree, or rocky crevices, on alpine volcanic slopes between 4000–4200 m. Flowering in August.

### Specimens examined.

Mexico. **Mexico:** Monte Tlaloc, near summit of mountain, 4100−4140 m, 22 Aug 1958, J.H.Beaman 2342 (US-2381582, TEX, WIS).

### Discussion.

**Figure 9.**
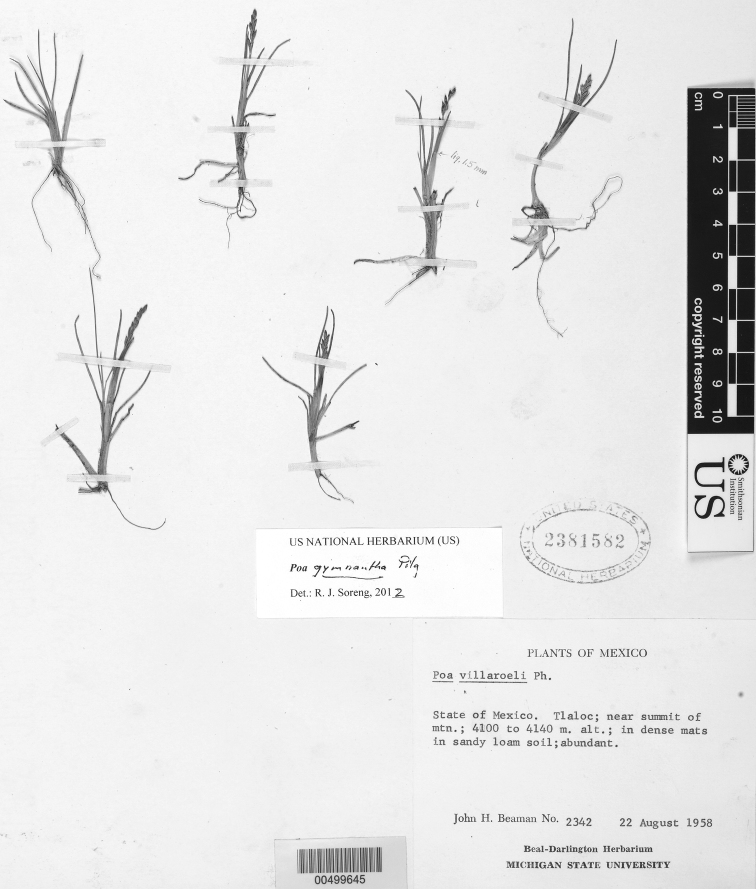
*Poa gymnantha* Pilg. Photo of *Beaman 2342*.

This is the first report of this species for Mexico. *Poa gymnantha* is known from the high Andes (ca. 8–16°S lat.; Negritto et al. 2008) in Argentina (Jujuy and Salta), Chile (Region 1 and Parinacota), Bolivia (La Paz, Oruro, and Potosí), Peru (Ancash, Apurimac, Arequipa, Ayacucho, Cuzco, Huancavelica, Junín, Moquegua, Puno, and Tacna). Negritto et al. (2008) discusses the taxonomy and reproductive biology of this high polyploid, pistillate, apomictic species. Although low growing forms, often treated as *Poa ovata* and *Poa pseudoaequigluma* (see synonyms above)are excluded from *Poa gymnantha* sensu Negritto et al. (2008), we have made over 84 collections of this species from across its Andean range, and examined many other collections at LPB, US, USM. We cannot find a single morphological feature that can be used to separate these taxa, and instead only see a range or continuum of these features across the entire range. Negritto in Giussani et al. (2012) now accepts *Poa ovata* and *Poa pseudoaequigluma* as synonyms with expressed need for further study. The description provided here is based on one small Mexican collection, with extreme ranges from South American material noted in parentheses [given as “(?)" where the full character state range was not documented for South America samples]. In South America small and large plants (*Poa gymnantha* s.s.) are often mixed within populations, and the stature appears to depend on elevation and microhabitat variations in moisture and light intensity, and exposure to herbivory. Although the type and few other specimens of *Poa ovata* have well developed stamens, hundreds of other specimens examined have only staminodes and regularly produce seed, a situation that indicates apomixis (Soreng and Van Devender 1989; Negritto et al. 2008). John Beaman (notes in US herbarium) intended to describe his no. *2342* as a new species, with the epithet “acrophila". The features that join the Mexican collection with *Poa gymnantha* s.l. are the small stature (5 to 6 cm tall); very narrow, contracted panicles (most like the type of *Poa pseudoaequigluma*); basal sheaths that become a bit fibrous in age; leaf-blades involute, abaxially smooth, with scabrous margins and densely scaberulous adaxial surfaces; ligules abaxially scabrous; lemmas that are glabrous, the apical 1/3−1/4 portion brown, scareous, and scaberulous; and florets pistillate. In contrast to *Poa chamaeclinos*, the tufts of *Poa gymnantha* are erect, not mat forming, leaf blades are erect to ascending, involute and adaxially densely scaberulous, the lemmas are distally scabrous with indistinct lateral veins. Although both species generally occur between 4000–5000 m, from our experience in the Andes, *Poa gymnantha* grows on dry slopes and plains, instead of perennially wet or “waterlogged" habitats. We provide a photo of the Beaman collection from Mexico (Fig. 9) but chose to illustrate a Peruvian specimen with 2-flowered spikelets (Fig. 6 A–E) because the Beaman specimens are quite depauperate and immature. In South America depauperate specimens of the species with one-flowered spikelets are fairly common.

## 10. Poa infirma

Kunth, Nov. Gen. Sp. (quarto ed.) 1: 158. 1815 [1816].

http://species-id.net/wiki/Poa_infirma

Fig. 2 F–H

Megastachya infirma (Kunth) Roem. & Schult., Syst. Veg., editio decima sexta 2: 585. 1817. *Eragrostis infirma* (Kunth) Steud., Nomencl. Bot. (ed. 2) 1: 563. 1840. *Ochlopoa infirma* (Kunth) H.Scholz, Ber. Inst. Lanschafts-Pflanzenokologie Univ. Hohenheim Beih. 16: 59. 2003.Type: Nova Granada, Aug 1801, *Humboldt& Bonpland 134* (holotype P-HUMB!; isotypes: B-WILLD*-*1974! pl. 223, LE-TRIN-2638.01 fragm. & illustr.!, US-1851276! fragm. ex P, US-2851277! fragm. ex P-HUMB).

### Description.

Gynomonoecious or hermaphroditic. **Annuals**; tufted, tufts mostly small, bases narrow, light green; tillers intravaginal (each subtended by a single 2-keeled, longitudinally split prophyll over 0.5 cm long), without cataphyllous shoots, most shoots flowering. **Culms** 2–18 cm tall, spreading to erect, sometimes geniculate, slender, leafy, terete, smooth; usually 1 node exerted. **Leaf** sheaths terete or weakly compressed, smooth, glabrous; butt sheaths thin papery; flag leaf sheaths 1–5 cm long, margins fused ca. 33% their length; throats and collars smooth, glabrous; ligules 0.5–3 mm long, decurrent, abaxially smooth, glabrous, apices obtuse to truncate; blades 1–7 cm long, 1–3(–4) mm wide, flat or weakly folded, thin, soft. smooth, margins usually slightly scabrous, broadly prow-tipped; blades all about equal in length, flag leaf blades well developed. **Panicles** 1–6 cm long, 1.5–3 × long as wide, erect, more or less open, rhomboid, moderately congested; rachis with 1–2(–5) branches per node; primary branches mostly ascending, straight, terete or sulcate, smooth; lateral pedicels less than 1/5 the spikelet in length, smooth; longest branches 1.5–3 cm, spikelets crowded along the branches, with up to 10 spikelets from the base to distal 1/2. **Spikelets** 3–5 mm long, lanceolate, laterally compressed; not bulbiferous; florets 2–6, proximal hermaphroditic, distal sometimes pistillate; rachilla internodes terete, smooth, glabrous, usually exposed in side view, distal internode 1/2–3/4 length of distal lemma; glumes unequal, smooth, distinctly keeled, keels smooth, apex acuminate to acute or obtuse, sharp pointed or slightly blunt; lower glumes 1–1.5 mm long, 1-veined, narrowly lanceolate, often slightly sickle shaped, or subulate; upper glumes 2–2.5 mm long, usually shorter than or subequaling lowest lemma, 3-veined, lanceolate to oblanceolate; calluses glabrous; lemmas 2–2.5 mm long, broadly lanceolate, light green, distinctly keeled, smooth throughout, keels, marginal, and intermediate veins densely crisply puberulent to long villous, between veins glabrous, intermediate veins prominent, margins and edges smooth, apices obtuse to acute; paleas keels smooth, short to long villous over the keels. **Flowers** cleistogamous to weakly chasmogamous; lodicules 0.15–0.2 mm long (the upper sometimes rudimentary); anthers 0.1–0.55 mm long, more or less spherical to short elliptical prior to dehiscence, distal flower ones sometimes vestigial. **Caryopses** 1.4 mm long, elliptical in side-view, subcylindrical in cross-section, pale green, sulcus almost flat, hilum 0.1 mm long, round to oval, grain slightly adherent to the palea. 2n = 14.

### Distribution.

The species is indigenous to western Eurasia, Middle East (especially Mediterranean countries), and North Africa; introduced in Australia and the Americas. In North America the species is known from sporadic locations in British Columbia, Canada; California, Georgia, Oregon in the USA; and Baja California, Mexico. In South America the species is known from Argentina, Bolivia, Chile, Columbia, Peru, and in Central America it has been reported from Guatemala (Soreng et al. 2003b).

### Ecology.

This species occurs from near sea level in temperate regions with Mediterranean climates, to elevations with cool temperate to frigid climates in tropical latitudes (to 4400 m). Flowering late winter to early spring.

### Specimens examined.

Mexico. **Baja California:** between Maneadaro and San Carlos Hot Springs, 18 Apr 1973, A.A.Beetle M-2838 (TAES).

### Discussion.

This diploid species name was applied to various early collections from Mexico, and later treated as synonym of *Poa annua* (Hitchcock 1913, 1935). *Poa annua* is a tetraploid species derived from *Poa infirma* × *Poa supina* (Soreng et al. 2010) that sometimes looks quite similar to its parental types, making identifications challenging. *Poa infirma* has more crowded and small spikelets on branches that are more ascending, in addition to shorter anthers [0.2−0.5(–0.6) mm], and is a short-lived ephemeral. Soreng et al. (2003a) cited *Poa infirma* for Mexico, but review of the US vouchers by Hildemar Scholz (B), and subsequently again by RJS and also for MO and MEXU vouchers did not reveal any authentic material. Since then, one authentic specimen was found in a loan from TAES. It is expected to be present elsewhere in Mexico. It is well established in lower elevations of central and southern California west of the Sierra Nevada, and occurs at scattered high elevation locations from Colombia south to Argentina. One old collection from Guatemala, 1880s, *H.vonTürckheim 907* (US) originally distributed as *Poa infirma*, was redetermined by H. Scholz (det. 2007) as *Ochlopoa maroccana* (Nannf.) H. Scholz (≡*Poa maroccana* Nannf.). This is the only US specimen from the New World that he determined as this species, and RJS redetermined (det. 2011) it as “*Poa infirma*?" Only one anther (0.3 mm long) was found on this specimen, pointing to *Poa infirma*, but the panicles are short and spreading, and more similar in aspect to *Poa annua*.

## 11. Poa matris-occidentalis

P.M. Peterson & Soreng, Sida 22(2): 906, 908, f. 1a–c, 2c–l, 3a–c, 4. 2006.

http://species-id.net/wiki/Poa_matris-occidentalis

Figs 10 11

### Type:

Mexico, Durango, Sierra Madre Occidental, southwest slope of Cerro Gordo, just below twin rock outcrops, 23°12'32.5" N, 104°56'54.1"W, 3130−3200 m, 26 Sep 2005, *P.M.Peterson & F.Sánchez-Alvarado 19145* (holotype: US!; isotypes: CIIDIR!, MEXU!).

### Description.

Hermaphroditic. **Perennials**; tufted, sub-rhizomatous, tufts fairly dense to loose, of moderate girth and height, dark green; tillers mainly extravaginal (basally cataphyllous), with lateral or downward tending, brownish, cataphyllous shoots. **Culms** 45–80 cm tall, erect or bases slightly decumbent, leafy, terete or weakly compressed, smooth; nodes terete, 2–4, 1–3 exerted. **Leaf** sheaths compressed, distinctly keeled with a short wing to 0.5 mm deep, smooth, glabrous, or the lower ones sometimes retrorsely scabrous or puberulent; butt sheaths cataphyllous, brownish, smooth, glabrous; flag leaf sheaths 10–15 cm long, margins fused 66–80% the length, 0.4–1.1 × longer than its blade; collars smooth or lightly scabrous, glabrous or ciliate; ligules 3.5–6 mm long, scarious-white to hyaline, abaxially smooth, glabrous, or sometimes puberulent, apex obtuse to acute, entire; blades mostly 10–30 cm long, 2–6 mm wide, flat, to broad-V shaped, thin, lax, abaxial surface and margins lightly scabrous along the veins, adaxially smooth, glabrous throughout, with about 17 veins expressed, apices narrowly prow-tipped; mid-cauline blades 20–30 cm long, ca. 2 × longer than the flag leaf blades, flag leaf blades 12–22 cm long; sterile shoot blades similar to cauline blades. **Panicles** 12–26 cm long, nodding, open, pyramidal, sparse, with 24–85 spikelets, proximal internode 2.5–5.5 cm long; rachis with (1–)2(–3) branches per node; primary branches ascending to spreading, slender, flexuous, lax, angled, angles sparsely to moderately scabrous; lateral pedicels on average as long as spikelets, moderately scabrous, prickles of moderate coarseness; longest branches 5.5–10 cm, with 3–15 spikelets in the distal 1/3–1/2. **Spikelets** 4–8 mm long, 1.8–2.7 mm wide, broadly lanceolate, laterally compressed, not bulbiferous, greenish to stramineous; florets 2–3, hermaphroditic; rachilla internodes terete, 1–2 mm long, usually hidden, smooth, glabrous; glumes lanceolate, sub-lustrous, equal to subequal, distinctly keeled, keels scabrous distally, upper surfaces often lightly scabrous, edges smooth or lightly scabrous, apex narrowly acute, lower glumes 3–5 mm long, (1–)3-veined (laterals often short), narrowly lanceolate; upper glumes 3.7–5.6 mm long, distinctly 3-veined, lanceolate to oblanceolate; calluses dorsally webbed, web distinct, hairs 2–3 mm long, woolly; lemmas 4.6–6.3 mm long, lanceolate, 5-veined, green, distinctly keeled, keel and marginal veins glabrous or sometimes proximally sparsely puberulent, distally scabrous, between veins, muriculate to densely scabrous from near the base, intermediate veins distinct, upper margins narrowly scarious-hyaline, edges lightly scabrous, apices acute to narrowly acute, sometimes briefly purple and bronze tinged; paleas 4.4–6 mm long, usually distinctly shorter than the lemma, keels long scabrous for most of the length, between the keels moderately muriculate to short aculeolate. **Flowers** chasmogamous; lodicules (0.3–)0.6–0.8 mm long, broadly lanceolate to ovate, with a lateral lobe; anthers 2–2.2 mm long, infrequently those of distal flower abortive. **Caryopses** 2.6–3 mm long, fusiform in side-view, laterally compressed, subtrigonous in cross-section, light brown to olivaceous, sulcus distinct narrow, hilum 0.2–0.25 mm long, oval, grain adherent to the palea. 2*n* = unknown.

### Discussion.

Originally spelled as *Poa* “*matri-occidentalis*", the epithet is correctly spelled as *matris-occidentalis* (fide Kanchi Gandhi). The species is endemic to high mountains on the west side of the central Sierra Madre Occidental in southern Chihuahua to southwestern Durango (Peterson et al. 2006), and is only known from two peaks that are over 300 km apart. Specimens have sometimes passed under the name *Poa tracyi* Vasey, a species of the mountains of Colorado and New Mexico (Soreng and Hatch 1983, Soreng 1985, 2007). DNA data (Gillespie and Soreng, unpublished, from the holotype) supports the species placement within *Poa* subgen. *Poa* supersect. *Homalopoa*, rather than in *Poa* sect. *Sylvestres* as originally postulated in Peterson et al. (2006) based on tenuous morphological connections.

**Figure 10.**
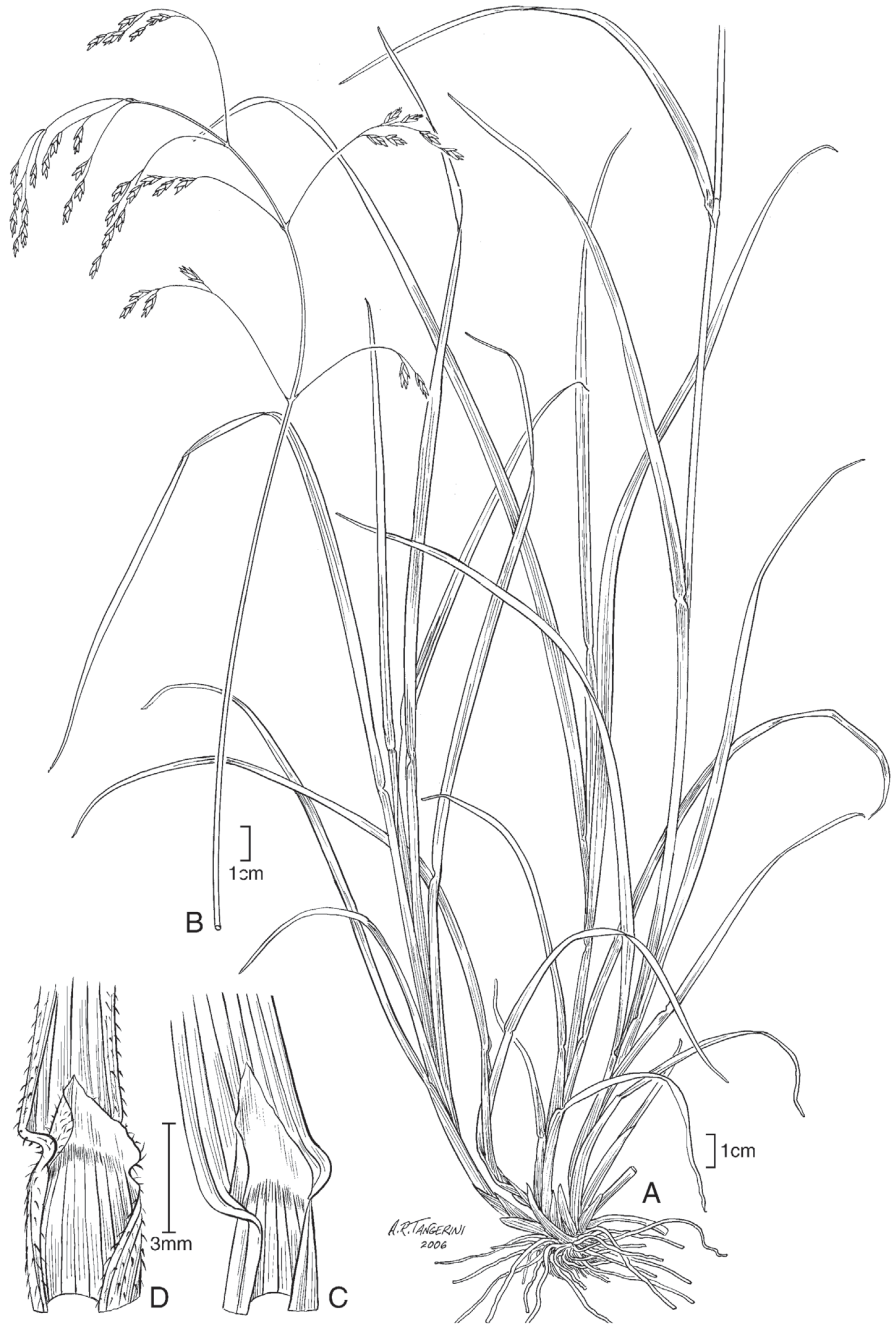
*Poa matris-occidentalis* P.M. Peterson & Soreng. **A–C** *Poa matris-occidentalis* subsp. *matris-occidentalis* **A** habit **B** inflorescence **C** sheath, ligule, and blade **D** subsp. *mohinorensis* Soreng & P.M. Peterson **D** sheath, ligule, and blade. Drawings from Peterson et al. (2006), **A–C** drawn from holotype collection (*Peterson 19145 & Sánchez-Alvarado*) **D** drawn from holotype collection (*Nesom 6475 & McDonald*).

**Figure 11.**
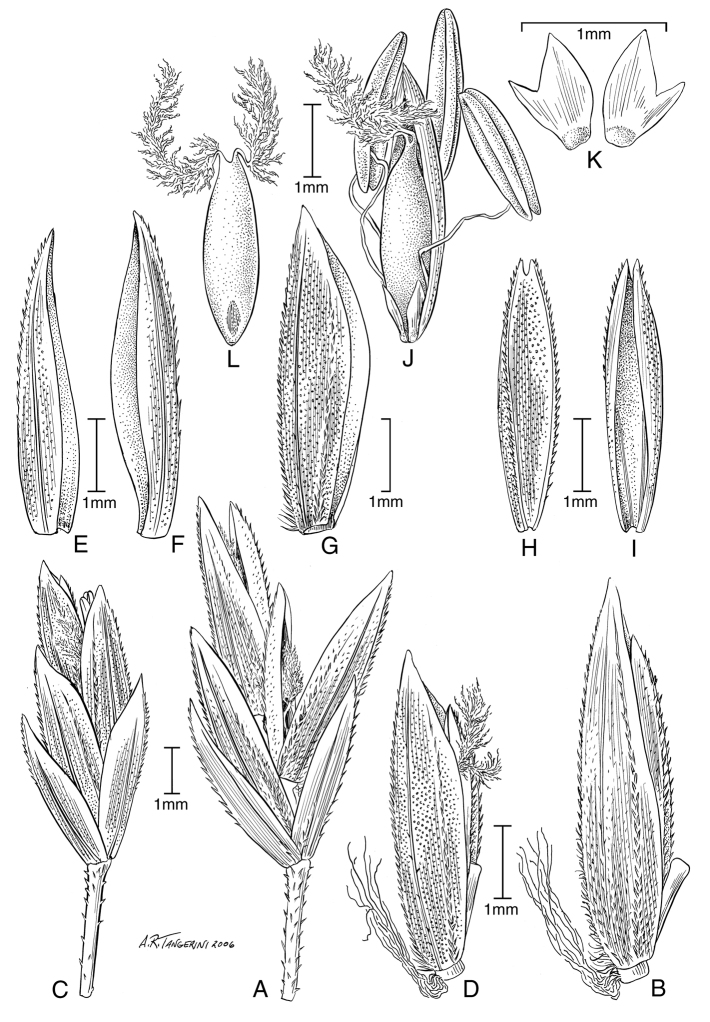
*Poa matris-occidentalis* P.M. Peterson & Soreng. **A, B** *Poa matris-occidentalis* subsp. *mohinorensis* Soreng & P.M. Peterson **A** spikelet **B** floret; **C–L** subsp. *matris-occidentalis* **C** spikelet **D** floret **E **lower glume **F** upper glume **G** floret **H** palea dorsal view **I** palea ventral view **J** perfect flower, stamens, ovary, and lodicules, all enclosed in palea **K** lodicules **L** young caryopsis. Drawings from Peterson et al. (2006), **A, B** drawn from holotype collection (*Nesom 6475 & McDonald*) **C–L** drawn from holotype collection (*Peterson 19145 & Sánchez-Alvarado*).

### Key to the subspecies of *Poa matris-occidentalis*

**Table:** 

1	Sheaths of lower leaves smooth, glabrous; collars smooth or with a few hooks, glabrous; lemmas finely muriculate between the veins, keel and marginal veins glabrous below; plants from Durango	11a. *Poa matris-occidentalis* subsp. *matris-occidentali*s
–	Sheaths of lower leaves retrorsely scabrous to puberulent; collars ciliate; lemmas densely scabrous between the veins, keels and marginal veins puberulent below; plants from southern Chihuahua	11b. *Poa matris-occidentalis* subsp. *mohinorensis*

## 11a. Poa matris-occidentalis matris-occidentalis

http://species-id.net/wiki/Poa_matris-occidentalis_matris-occidentalis

Figs 10 A–C 11 C–L

### Description.

**Leaf** sheaths smooth, glabrous; collars glabrous; ligules abaxially glabrous. **Lemma** keel and marginal veins glabrous, between veins muriculate. **Lodicules** 0.3–0.4 mm long, broadly lanceolate, with a lateral lobe.

### Distribution.

This subspecies is known only from Cerro Gordo, Durango.

### Ecology.

Known only from a single locality at Cerro Gordo, between 3130–3200 m where the species was found growing on steep, rocky, and grassy slopes beneath open forests of *Pseudotsuga menziesii* (Mirb.) Franco, *Quercus* spp., *Pinus* spp., with other associates, such as: *Arctostaphylos pungens* Kunth, *Bromus carinatus* Hook. & Arn., *Bromus richardsonii* Link, *Muhlenbergia cenchroides* (Humb. & Bonpl. ex Willd.) P.M. Peterson, *Trisetum viride* (Kunth) Kunth, *Festuca* sp., *Carex* sp., and *Agrostis* sp. Flowering September and October.

### Specimens examined.

Mexico. **Durango:** Transect from Paseo de Cerro Gordo to the top (cumbre), 23°12'31.1"N, 104°56'53.0"W, 3136−3348 m, 9 Sep 2006, P.M.Peterson 20011 & F.Sánchez-Alvarado (CIIDIR, US).

### Discussion.

Cerro Gordo is quite remote from other high peaks in the area as it takes nearly 10 hours of driving from Durango along a dirt road to reach the base of the mountain, just south of Maiz Gordo. There are other mountains in Durango that approach this height and it would seem reasonable to assume that this species might occur on these.

## 11b. Poa matris-occidentalis mohinorensis

Soreng & P.M. Peterson, Sida 22(2): 911, f. 1d, 2a–b. 2006

http://species-id.net/wiki/Poa_matris-occidentalis_mohinorensis

Figs 10 D 11 A, B

### Type:

Mexico, Chihuahua, Municipio de Guadalupe y Calvo, Sierra Madre Occidental, N side of Cerro Mohinora, ca. 13 mi SW of Guadalupe y Calvo, open pine-fir woods with scattered spruce, nearly vertical, N facing rock wall, very moist with many bryophytes and rich herbaceous flora, 25°57'N, 107°03’W, 2950 m, 20 Aug 1988, *G.Nesom & A.McDonald 6475* (holotype: TEX!; isotype: ARIZ!).

### Description.

**Leaf** sheaths retrorsely scabrous to puberulent; collars ciliate; ligules abaxially puberulent. **Lemma** keel and marginal veins proximally puberulent, between veins densely scabrous. **Lodicules** 0.75 mm long, broadly ovate with a broad lateral lobe.

### Distribution.

This subspecies is known only from the Sierra Mohinora, Chihuahua.

### Ecology.

The subspecies is found on moist rocky ledges and cliffs associated with *Juniperus*, *Pinus*, *Abies*, *Picea*, *Holodiscus*, *Silene*, *Cerastium*, *Deschampsia cespitosa* (L.) P. Beauv., *Bromus carinatus*, *Muhlenbergia vaginata* Swallen, and *Muhlenbergia cenchroides*; from 2950–3300 m (see below, Correll’s estimation is too high according to the geodetic mark at the top of the peak). Flowering August to October.

### Specimens examined.

Mexico. **Chihuahua:** Sierra Mohinora, near Cumbre Mohinora, 25°57'34"N, 107°03'49"W, 3250−3300 m, 13 Sep 2006, P.M.Peterson 20048, F.Sánchez-Alvarado & E.P.Gómez-Ruíz (US); ditto, 20049 (US); ditto, on summit, 10,000–12,000 ft [3050−3660 m], 16−17 Oct 1959, D.S.Correll 23177 & H.S.Gentry (LL).

### Discussion.

When the second author visited the type locality in 2006, the habitat within 5 km of the peak was found to be quite disturbed by grazing and logging. The holotype was initially determined as *Poa tracyi* “vel. aff." by J.R & C.G. Reeder in 1989).

## 12. Poa mulleri

Swallen, J. Wash. Acad. Sci. 30(5): 211. 1940.

http://species-id.net/wiki/Poa_mulleri

Figs 12 13 A−D

### Type:

Mexico, Nuevo León, Municipio de Galeana, collected in pine woods on the Peak of Cerro Potosí, 21 Jul 1935, *C.H. Meuller 2251* (holotype: US-1645320!; isotypes: GH!, US-1646008!).

### Description.

Hermaphroditic. **Perennials**; tufted, tufts dense, fairly small and low (mostly 6–10 cm tall), pale green, to slightly bluish-grey-green; tillers intravaginal (each subtended by a single elongated, 2-keeled, longitudinally split prophyll), and extravaginal (basally cataphyllous), without lateral or downward tending, cataphyllous shoots, sterile shoots more numerous than flowering shoots. **Culms** 9–28(–42) cm tall, erect to loosely ascending, sometimes decumbent or geniculate, terete, smooth; nodes 2–3, upper 0–1(–2) exerted, uppermost at mid-culm. **Leaves** mostly basal; leaf sheaths slightly keeled, smooth, glabrous; butt sheaths papery or becoming slightly fibrous in age, smooth glabrous; flag leaf sheaths 4–8(–11) cm long, margins fused 13–25% their length, sheath ca. 2–6 × longer than its blade; collars smooth, glabrous; ligules 0.25–0.8 mm long, abaxially minutely scabrous, upper margin irregular, minutely scabrous; apex truncate, sterile shoot ligules like those of the culm leaves; blades to 8(–12) cm long, 1–2 mm wide, involute, margins inrolling, soft, moderately thick, abaxially smooth, papilliate, veins slightly expressed, margins scabrous, adaxially papilliate, slightly scabrous over the costae, apex prow-tipped; culm blades more or less equal in length or the middle ones longest, flag leaf blades 0.9–3.5 cm long; sterile shoot blades like those of the culm blades. **Panicles** 3.4–8 cm long, erect, open, pyramidal, exerted, fairly sparse, with 20–60 spikelet, peduncles and axis smooth or sparsely scabrous, proximal internode 0.7–2 cm long; rachis with branches (1–)2 per node; primary branches widely spreading to reflexed, slightly flexuous, weakly to distinctly angled, moderately to densely scabrous, hooks mostly on the angles, minutely papilliate; lateral pedicels mostly 1/5–1/2 the spikelet in length, sparsely to moderately coarse scabrous, densely papilliate; longest branches 1.4–3.8 cm, with 3–8 spikelets somewhat clustered, in the distal 1/2 to quarter. **Spikelets** 4–5 mm long, broadly lanceolate to lanceolate, laterally compressed, not bulbiferous, pale green, slightly anthocyanic; florets (1–)2, hermaphroditic; rachilla internodes to 1 mm long (extension to 1.5 mm), smooth, glabrous; glumes broadly lanceolate to lanceolate, usually somewhat anthocyanic, subequal to equal, slightly shorter than adjacent lemmas, distinctly veined, distinctly keeled, slightly thinner than lemmas, smooth or keel apically slightly scabrous, surfaces obscurely papilliate, apex acute; lower glumes 2.5–3.2 mm long, 3-veined; upper glumes 3–3.5 mm long, 2 × to equal lower glumes in width, (3–)5(–7)-veined; calluses glabrous; lemmas 3–4 mm long, broadly lanceolate, lower one 5–7-veined, upper 5-veined, green with or without anthocyanic flush, distinctly keeled, keel smooth or distally sparsely scabrous, keel and marginal veins glabrous or thinly and loosely puberulent, between veins glabrous or loosely puberulent in lower 1/3, intermediate veins prominent, margins and apex narrowly scarious hyaline, edges smooth, apices acute, incurved; palea keels smooth or sparsely to moderately scabrous, glabrous, between keels glabrous or sparsely puberulent. **Flowers** chasmogamous; lodicules 0.75 mm long, broadly lanceolate, apex sharp, with a well developed lateral lobe; anthers 1.6–2 mm long, sometimes poorly formed. **Caryopses** 1.8 mm long, elliptical in side-view, round on back, almost cylindrical in cross-section, pale brown, sulcus very shallow, hilum 0.25 mm long, round to oval to elliptical, grain free from the palea. 2*n* = unknown.

### Distribution.

The species is known only from the Cerro Potosí, Nuevo León.

### Ecology.

The species occurs on open or sparsely wooded slopes derived from calcareous rocks on the upper slopes of Cerro Potosí, and is associated with *Pinus culminicola* Andresen & Beaman, *Trisetum spicatum* (L.) K. Richt., *Senecio* sp., and *Festuca hephaestophila* Nees ex Steud., and *Festuca hintoniana* E.B. Alexeev; between 3650−3800 m. Flowering July to August.

### Conservation status.

This narrow endemic is locally uncommon.

### Specimens examined.

Mexico. **Nuevo León:** Cerro Potosí, ca. 20 mi NE of Galeana, ascent of Sierra Potosi by the north hogback, 26 Jul 1934, C.H.Mueller 1248 & M.T.Mueller (GH, MEXU, TEX); ditto, summit, ca. 3650 m, 1 Jul 1959, J.H.Beaman 2643 (GH, MSC, TEX, US). at NE summit of mountain, ca. 3650 m, 13 Sep 1960, J.H.Beaman 4470 (GH, MSC, TEX, US); ditto, 25 Mar 1962, A.A.Beetle M-475 & P.Rojas-M. (WYAC); ditto, 20°52'23"N, 100°13'48"W, 3650 m, 15 Aug 1998, Ing.M.Castillo-B. 345 & Ing.J.Garza-C. (MEXU & MEXU p.p. “b", p.p. “a" is *Poa pratensis* subsp. *alpigena* fide RJS on both sheets); ditto, near summit, 3674 m, 21 Oct 2007, P.M.Peterson 21459, J.M.Saarela, & D.Stančik (US; DNA voucher, unpublished); ditto, just below summit, 26 Jul 1985, S.Ginzbarg 217, A.Whittemore & A.McDonald (TEX). Municipio Galeana, 3800 m, 21 Aug 1969, G.B.Hinton 17253 et al. (TEX); ditto, Cima del Cerro Potosí, 3670 m, 3 Aug 1988, A.Garcia 70 (MEXU). 3660 m, 15 Aug 1989, A.Garcia 163, S.Gonzales & M.Gonzalez (MEXU).

### Discussion.

*Poa mulleri* is an odd species perhaps related to *Poa orizabensis*, but the characters are quite unusual, and it deserves a subsection of its own. Papillae on long cells of the leaf blade in *Poa* are known from some species of *Poa* sect. *Secundae* subsect. *Halophyllae* Soreng, and from *Poa arida* Vasey and a few other species. No other species of the genus are known to have multiple small papillae per cell, a character state that to our knowledge is novel in the tribe Poeae, and possibly in Pooideae but is common in Bambusoideae and Ehrhartoideae (Metcalfe 1960). Upper glumes in *Poa* are almost invariably 3-viened, and when 3 to 5 veins occur, the 5-veined state is infrequent within a species (e.g., *Poa macrantha* Vasey). In *Poa mulleri*, the upper glumes are 5–7-veined and the lower lemmas of each spikelet are 5–7-veined. Preliminary DNA data place it in the large supersect. *Homalopoa* clade (L. Gillespie and N. Amiri pers. com. 2012), for which the sectional and infrasectional taxonomy remains poorly resolved (Gillespie et al. 2009; Soreng et al. 2009).

**Figure 12.**
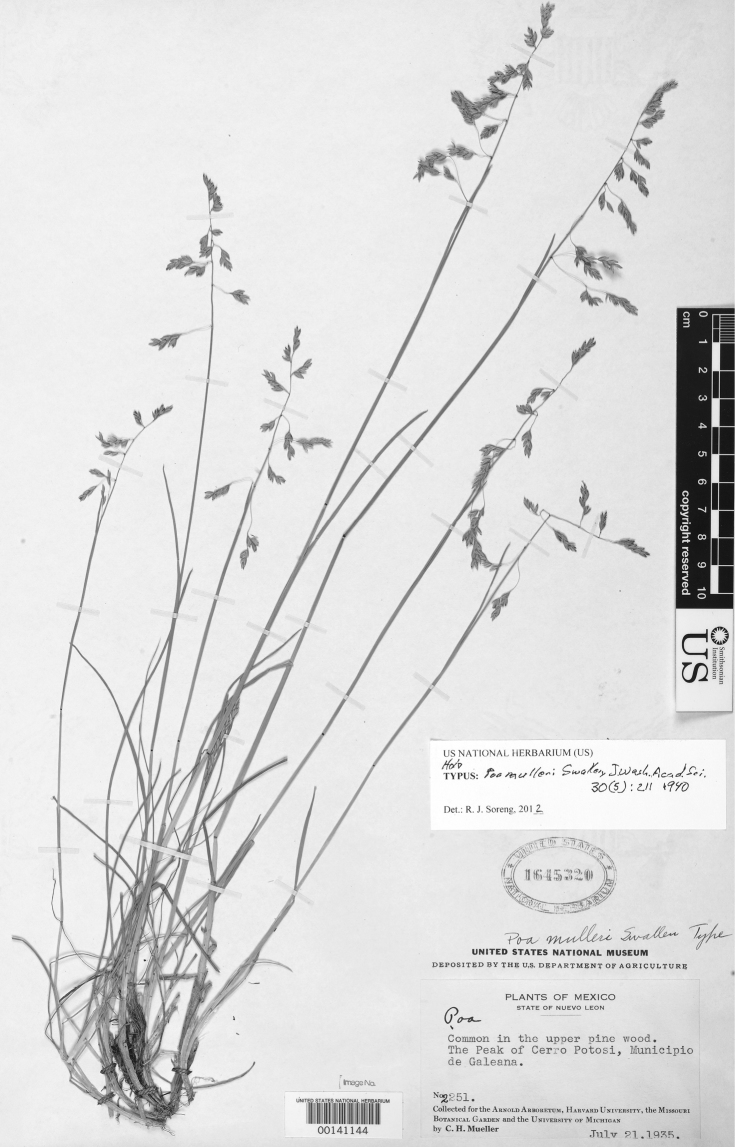
*Poa mulleri* Swallen. Photo of holotype collection (*Mueller 2251*).

**Figure 13.**
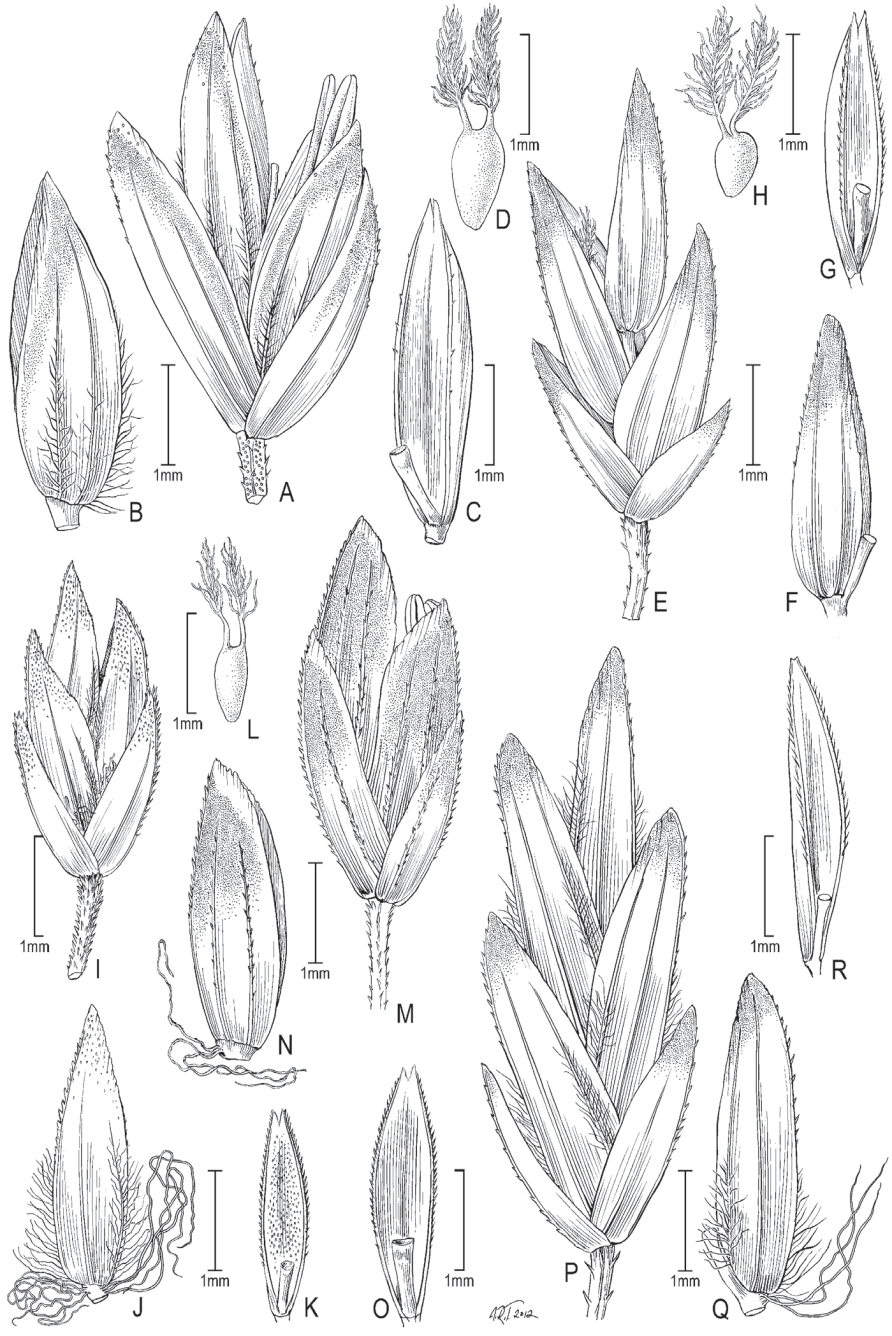
**A–D** *Poa mulleri* Swallen **A** spikelet **B** floret **C** palea **D** pistil **E–H** *Poa seleri* Pilg. **E** spikelet **F** floret **G** palea **H** pistil **I–L** *Poa scaberula* Hook.f. **I** spikelet **J** floret **K** palea **L** pistil **M–O** *Poa orizabensis* Hitchc. **M** spikelet **N** floret **O** palea **P–R** *Poa ruprechtii* Peyr. **P** spikelet **Q** floret **R** palea. **A–D** drawn from *Beaman 4470* **E–H** drawn from*deKoninck 134* from Guatemala **I–L** drawn from *Peterson 11087 et al* **M–O** drawn from *Soreng 3314 & Soreng* **P–R** drawn from *Davidse 9771*.

## Poa Papillopoa

Soreng subsect. nov.

urn:lsid:ipni.org:names:77121281-1

### Type.

*Poa mulleri* Swallen.

### Diagnosis.

From other infrageneric taxa of *Poa*, differing by the 5−7-veined upper glumes, and long cells of the leaf blade abaxial epidermis with a central line of 6–9 papillae per cell.

## 13. Poa occidentalis

(Vasey) Vasey, Contr. U.S. Natl. Herb. 1(8): 274−275. 1893.

http://species-id.net/wiki/Poa_occidentalis

Fig. 4 F–M

Poa trivialis var. *occidentalis* Vasey, Descr. Cat. Grass. U.S. 85. 1885. Type: USA, New Mexico, Las Vegas, 1881, *G.R. Vasey s.n*. (lectotype: US-79610! designated by Vasey 1893: 275; isolectotypes: S-G-6757! fragm., S-G-6758! fragm., US-156871!, US-824855!, US-919188!).

### Description.

Hermaphroditic. **Perennials**, short-lived; tufted, tufts fairly dense, usually medium girth and height, bases narrow or moderately wide, green, to bluish-green; tillers intravaginal (each subtended by a single elongated, 2-keeled, longitudinally split prophyll), and extravaginal (basally cataphyllous), all erect. **Culms** 20–110 cm tall, erect, slender to stout, leafy, slightly compressed, smooth or scabrous; nodes terete, (3–)4–6, (1–)2–4 exerted. **Leaf** sheaths distinctly compressed and keeled, usually densely retrorsely scabrous, rarely lightly scabrous, margins not ciliate; butt sheaths papery in age, smooth, glabrous; flag leaf sheaths 5–22 cm long, margins fused (20–)15–50(–60)% the length, subequal to its blade; ligules 3–12 mm long, abaxially densely scabrous, apices acute to acuminate; blades (1.2–)1.5–6(–10) mm wide, flat, lax, abaxial and abaxial surfaces and margins scabrous along the veins, broadly prow-tipped; flag leaf blades 4–18(–30) cm long. **Panicles** (6–)12–40 cm long, nodding, lax, eventually open, pyramidal, moderately congested, spikelets numerous, to 200+; rachis with (2–)3–7 branches per node; primary branches eventually spreading, drooping, angled, angles densely scabrous; lateral pedicels mostly 1/4–1/2 the spikelet in length, scabrous; longest branches (3–)5–18(–23) cm, with (5–)8–40(–120) spikelets. **Spikelets** (3–)4–7(–8) mm long, ovate, laterally compressed; not bulbiferous; florets 3–7, hermaphroditic; rachilla internodes terete, less than 1 mm long, smooth or slightly muriculate, glabrous; glumes distinctly keeled, keels scabrous, apex acuminate; lower glumes 2–3.5 mm long, 1–3-veined; upper glumes 2.5–4.2 mm long, shorter than or subequaling lowest lemma, 3-veined; calluses dorsally webbed, web distinct, hairs woolly; lemmas 2.6–4.2 mm long, narrowly lanceolate, distinctly keeled, scabrous distally, keels to 1/2 and marginal veins to 1/3 short to long villous, intermediate veins and between veins usually sparsely softly puberulent, intermediate veins prominent, apices narrowly acute; paleas scabrous, glabrous over the keels. **Flowers** mainly cleistogamous; lodicules 0.6 mm long, lanceolate; anthers 0.3–1.0 mm long. **Caryopses** 2 mm long, elliptical-fusiform in side-view, strongly laterally compressed, light brown, sulcus narrow, shallow, hilum 0.2 mm long, elliptical, grain adherent to the palea. 2*n* = 14, 28.

### Distribution.

The species is known from Arizona, Colorado, New Mexico, and Texas, USA; and Coahuila, Mexico (Madera del Carmen).

### Ecology.

The species occurs on slopes in wooded canyons with *Pinus*, *Pseudotsuga*, *Cupressus*, *Abies*, *Ceanothus*, *Cornus stolonifera* Michx., *Bromus richardsonii* Link, *Festuca viridula* Vasey, *Festuca thurberi* Vasey, and *Quercus*;ranging between 2280–2550 m. Flowering August to September.

### Conservation status.

The species is rare in Mexico.

### Specimens examined.

Mexico. **Coahuila:** Madera del Carmen; canyon above Campo El Dos, 28°59'23.6"N, 102°36'43.0"W, 2280−2320 m, 8 Sep 2005, P.M.Peterson 18918 & J.Valdes-Reyna (US; DNA voucher, unpublished). 13.8 mi NE of Los Pilares, 28°57'13.0"N, 102°35'7.3"W, 2335 m, 21 Sep 2007, P.M.Peterson 20979, J.M.Saarela, S.Lara-Contreras & J.Reyna-Alvarez (US). 1.8 mi from Campo Uno, up the road towards the summit, 29°0'14.2"N, 102°36'22.7"W, 2547 m, 23 Sep 2007, P.M.Peterson 21036 J.M.Saarela, S.Lara-Contreras & J.Reyna-Alvarez (US).

### Discussion.

This is the first report *Poa occidentalis* for Mexico, and is a range extension of 460 km to the southeast. This species was previously known to occur from eastern Arizona (White Mountains) to southern Colorado, south through the mountains of New Mexico to the Guadalupe Mountains of Eddy County, Texas (Soreng 1985, 2007). Three new collections were gathered in northern Mexico in 2005 and 2007, in the upper coniferous forest belt of the Madera del Carmen, and these presumably represent a relictual population. The first glume of *Poa occidentalis* was reported as 1-veined (Soreng ibid.), whereas it is principally 3-veined in the Mexican specimens, but re-examination of New Mexican specimens revealed this character state to vary from 1 to 3 veins. *Poa occidentalis* can be distinguished from other species *Poa* in Mexico by the following characters: its tall stature; short-lived perennial nature; long acute to acuminate, abaxially scabrous ligules that are longer than the leaf blades are wide; long and widely open, many spikeleted panicles; and anthers 0.5−1.0 mm long. Vasey (1893) considered his *Poa trivialis* var. *occidentalis* published in 1885 to be “without description", and taxonomists have traditionally accepted this. However, in 1885, he provided an adequate diagnosis of his indigenous *Poa occidentalis* var. *occidentalis*, from Colorado and New Mexico, by comparing it to the introduced *Poa trivialis* L., which he described and noted had been established in the United States outside of cultivation (mainly in the eastern USA at low to moderate elevations). The isolectotypes indicate “July 1881", and “Mts. near Las Vegas", or “Mts. west of Las Vegas", but there is no habitat “at Las Vegas" for the species, we presume all the material was collected in the mountains near or west of Las Vegas and to be of the same gathering as the lectotype. Vasey’s collection notebooks at US are not helpful and there is only one listing (no. 566) as *Poa trivialis* under Arizona and New Mexico 1881 (without other details).

## 14. Poa orizabensis

Hitchc. Contr. U.S. Natl. Herb. 17(3): 374 1913.

http://species-id.net/wiki/Poa_orizabensis

Figs 13 M–O 14

### Type:

Mexico, Puebla, Mt. Orizaba, bald hill, 13,000 ft [3940 m], spreading, infrequent, 17−18 Aug 1910, *A.S.Hitchcock 6254* (holotype: US-691227!; isotype: ISC-66032!).

### Description.

Hermaphroditic. **Perennials**; tufted, tufts usually dense, generally medium small girth and height ([5–]10–20 cm tall), green to bluish-grey-green; tillers all or mainly extravaginal (basally cataphyllous), without lateral or downward tending, cataphyllous shoots, or with very short thin ones, sterile shoots more numerous than flowering shoots. **Culms** 25–60 cm tall, erect or loosely ascending, leafy, terete or weakly compressed, smooth; nodes 2–3, 1–2 exerted. **Leaf** sheaths strongly compressed, smooth, glabrous; butt sheaths becoming papery to somewhat fibrous, bases of butt sheaths glabrous; flag leaf sheaths 12–18 cm long, margins fused ca. 35% their length, ca.4 × longer than its blade; throats and collars smooth or slightly scabrous, glabrous; ligules 1.6–2 mm long, not decurrent, scarious, white, abaxially moderately to densely scabrous, upper margin erose to denticulate, asperous, apex truncate to obtuse to acute, sterile shoot ligules to 0.5 mm long, mostly shorter than those of the upper culm; blades 1–15 cm long, 2–4 mm wide, folded, somewhat thick and firm, margins involute, abaxially smooth or distally lightly scabrous, margins densely scabrous, adaxially nearly smooth or distinctly scabrous above costae, apex distinctly prow-tipped; flag leaf blades 1–3.5 cm long; sterile shoot blades like those of the culm. **Panicles** 7–13 cm long, erect or nodding, open, spreading or drooping, with 50–100 spikelets, peduncle scabrous, axis scabrous, proximal internode 1.5–3.8 cm long;; rachis with 1–2(–3) branches per node; primary branches spreading, slender, flexuous, slightly angled, proximally sparsely to moderately scabrous, distally densely scabrous on the angles lightly between them; lateral pedicels usually 1/4–1/2 the spikelet in length, sparsely to moderately scabrous angled, prickles coarse to moderately coarse, sometimes with papillae about the juncture with the glumes; longest branches 2–7 cm, with 10–25 spikelets crowded in distal 1/2. **Spikelets** 2.5–4.5 mm long, 2–3 × as long as wide, ovate, strongly laterally compressed, not bulbiferous, strongly anthocyanic; florets (1–)2(–4), hermaphroditic; rachilla internodes terete, mostly 0.4–0.8 mm long, smooth or muriculate, glabrous; glumes lanceolate, broadly lanceolate, or ovate, strongly anthocyanic, subequal, distinctly keeled, proximally smooth to scabrous most of the length, distally scabrous on the keel and lateral veins, margins narrowly scarious-hyaline, edges smooth or sparsely asperous, apex acute ; lower glumes 2.5–3.2 mm long, (1–)3-veined, 2/3–4/5 the lower lemma in length, lanceolate to broadly lanceolate; upper glumes 2.5–4.2 mm long, slightly shorter to slightly longer than the lowest lemma, 3-veined; calluses dorsally webbed, web scant, hairs 0.5–1 mm long, woolly; lemmas 2.7–3.4(–3.8) mm long, 5-veined (lowest sometimes 7-veined), ovate to broadly lanceolate, 2.6–4.2 × longer than wide, body firmly chartaceous, green below, apically anthocyanic, strongly keeled, keel and lateral veins and sparsely to densely scabrous, surfaces distally sparsely to moderately scaberulous, keel usually, and marginal veins sometimes, near the base sparsely puberulent, main lateral veins distinct, margins and apex narrowly white scarious to hyaline, apex entire, obtuse to acute; paleas glabrous, keels scabrous for most of the length. **Flowers** weakly chasmogamous; lodicules 0.35–0.4 mm long, broadly lanceolate, apex obtuse, blunt, with a broad lateral lobe; anthers 0.8–1.1(–1.25) mm long. **Caryopses** 1.5 mm long, elliptical in side-view, laterally compressed, light olivaceous-brown, sulcus distinct, hilum 0.2 mm long, oval, grain adherent to the palea. 2*n* = unknown.

### Distribution.

The species occurs in Mexico in the states of Mexico, Puebla, and Veracruz; and Guatemala.

### Ecology.

This species occurs in subalpine to low alpine meadows and slopes of volcanoes, between (2700–)3000–4300(–4570) m. Flowering July through September.

### Conservation status.

The species is endemic on the slopes of two volcanoes in Mexico where it is locally common. The disposition of some specimens from the Departamento of Huehuetenango, Guatemala, requires further study.

### Specimens examined.

Mexico. **Mexico:** Popocatepetl, 12000 ft [3660 m], 5−6 Aug 1910, A.S.Hitchcock 5988 (US). 9000−10000 ft [2725−3050 m], 5−6 Aug 1910, A.S.Hitchcock 6004 (US). Paso de Cortes, 3680 m, 17 Sep 1958, J.H.Beaman 2564 (MEXU). P.N. Izta-Popo, 8 Jul 1980, A.A.Beetle M-5190 (MEXU). Municipio de Tlalmanalco, region de la cabeza del Ixtaccihuatl, La Cienega, 3600 m, 18 Jul 1982, J.Rzedowski 37857 (MEXU). 17 km by road east of Amecameca highway 115 jct., W of Paso del Cortez, 19.097°N, 98.683°W, 3325 m, 2 Oct 1987, R.J.Soreng 3307 & N.Soreng (US). SW flank of Ixtaccihuatl 1 km N of La Joya Trail Head, 19.1507°N, 98.6525°W, 3965 m, 2 Oct 1987 R.J.Soreng 3312 & N.Soreng (US). Paso del Cortez between Popo and Ixtaccihuatl, 19.0858°N, 98.6471°W, 3660 m, 3 Oct 1987, R.J.Soreng 3314 & N.Soreng (DNA barcode voucher, unpublished, and Soreng 1990 cpDNA voucher). Municipio Ixtaccihuatl, Estacion Experimental de Investigation y Ensenanza de Zoquiapan, 8 km south de Rio Frio, Llano Colecto, 3350 m, 12 Sep 1975, S.D.Koch 75519 (TEX). **Puebla:** Pico de Orizaba, southwest side of mountain in Cañada, 3770 m, 10 Sep 1958, J.H.Beaman 2511 (MEXU, toward *Poa ruprechtii*). Sierra Negra, SE of Pico de Orizaba, east side of mountain, sandy gravelly soil in grassy meadow on moderately steep slope above timberline, ca. 4200 m., 10 Sep 1958, J.H.Beaman 2517 (TEX, US, WIS, toward *Poa ruprechtii*). 23 road km above Tlatlachichua, ca. 6 km NNW of Pico de Orizaba, near head of Rio Quatzalapa, ca. 2 km SW of Cerro Mihas, 19.086°N, 97.288°W, 3660 m, 4 Oct 1987, R.J.Soreng 3325 & N.Soreng (US). Municipio Cholula, 30 km E de Cholula, 3000 m, 30 Jun 1989, P.Dávila 391, P.Tenorio & J.Sanches-Ken (MEXU). Ixtaccihuatl, south side of mountain, ca. 6 km N of Paso de Cortes, ca. 3900 m, 18 Jul 1959, J.H.Beaman 2874 (US). **Puebla** or **Veracruz:** Mt. Orizaba, 14-15000 ft [4265−4570 m], 25–26 Feb 1892, J.G.Smith 571 (NY). Rio Frio (Camino de Puebla), Jul 1927, E.Lyonnet 68 (US).**Veracruz:** prope La Joya, 1836, Schiede s.n. (BAA, HAL, LE, US; basis of *Poa schiediana* Trin ex Steud., nom. nud.); Cordillera, Orizaba, Jun−Oct 1840, H.Galeotti 5767 bis (LE; basis of *Poa glycerioides* Rupr., nom. nud.).

### Discussion.

We have seen a few specimens from Guatemala that are tentatively placed here but verge on the limits accorded to *Poa ruprechtii*: Huehuetenango, San Juan Ixcoy, ca. del cruce para Chanchocal, 3200 m, *J. Rios 6229, N. Hernandez & M. Veliz* (MEXU), Sierra de los Cuchumatanes, at km 311 on Ruta National 9N (between Paquix and Chemal, ca. 3360 m, 2 Aug 1959, *J.H.Beaman 3011* (MO, US, US “P. atala" ined.). Also reported from Huehuetenango are *J.H.Beaman 3799b* (US), and *J.Rios s.n., N.Hernandez & M.Veliz* (MEXU). *Poa orizabensis* and *Poa ruprechtii* are difficult to separate when lemma pubescence is scantly developed in the latter, but as interpreted here the former has broader glumes and more compact spikelets, and the sides of the lemmas are glabrous. Further study is needed.

**Figure 14.**
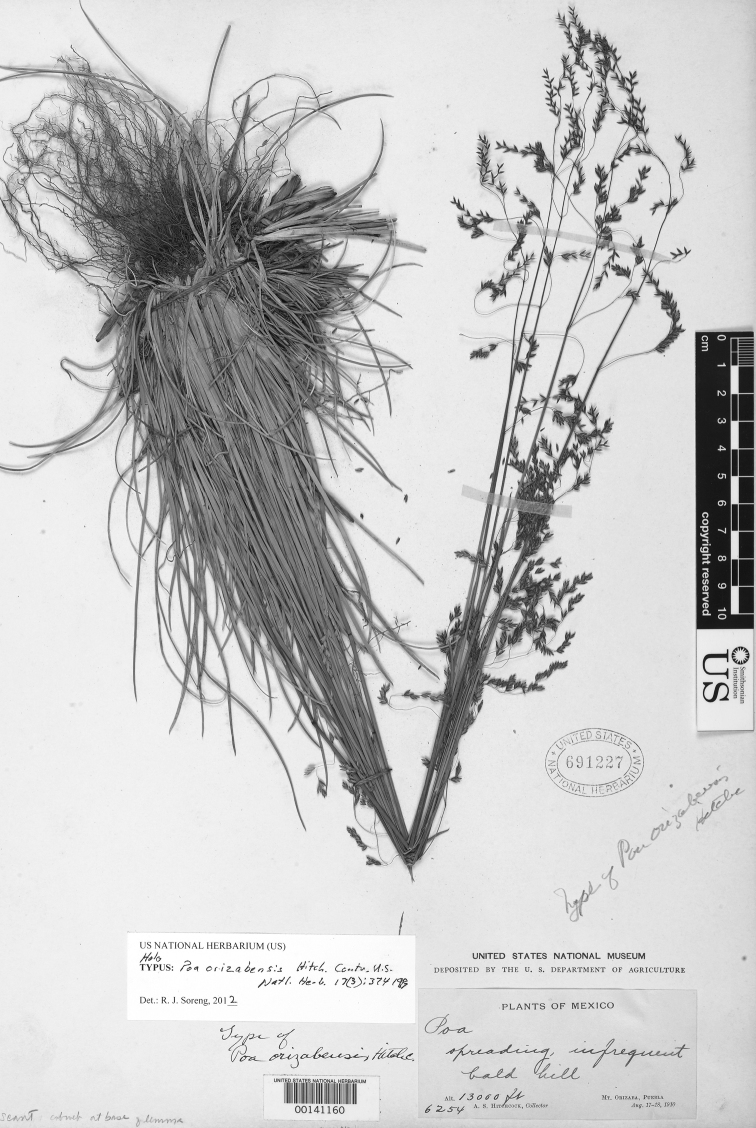
*Poa orizabensis* Hitchc. Photo of holotype collection (*Hitchcock 6254*).

## 15. Poa palmeri

Soreng & P.M. Peterson sp. nov.

urn:lsid:ipni.org:names:77121282-1

http://species-id.net/wiki/Poa_palmeri

Figs 6J 15

### Type:

Mexico. Nuevo León, Sierra Madre [Oriental] above Monterey [ca 25°38'N, 100°38'W], calcareous ledges, 3000 ft [misprint for 8000 ft; 2440 m], 31 Mar 1906, *C.G.Pringle 10212* (holotype: US-462254!♂; isotypes; GH!, MEXU-539725!♂, MO-1770420!♀, MSC, NMC!, NY-431382!, WYAC).

### Diagnosis.

Differing from *Poa ruprechtii* Peyr. by leaf collar margins flared (versus not flared), ligules of upper culm leaves truncate (versus obtuse to acute), flag leaf sheaths fused by an invaginated hyaline membrane for much of the upper fusion length (versus abruptly fused by firmly chartaceous margins), spikelet pedicles elongated and moderately to densely scabrous (versus shorter and usually densely to more coarsely scabrous), distal rachilla internodes usually 1–2 mm long (versus less than 1 mm), lemmas thinly chartaceous, intermediate veins faint to moderately prominent, not extending to near the upper margin, margin and apex fairly broadly scarious-hyaline (versus lemmas chartaceous, intermediate veins prominent, extending nearly to the margin, margin and apex narrowly scarious), anthers 1.6–2.8 (versus 0.7–1.05[–1.25]) mm.

### Description.

Trioecious (mostly hermaphroditic, but some pistillate or staminate). **Perennials**; tufted, sometimes sub-rhizomatous to rhizomatous, tufts dense to loose, generally medium girth and height, green or bluish-grey-green; tillers extravaginal (basally cataphyllous), and/or mainly intravaginal (each subtended by a single elongated, 2-keeled longitudinally split prophyll), with lateral or downward tending, cataphyllous shoots, or without them. **Culms** (26–)50–70 cm tall, erect or bases slightly decumbent, fairly slender, not branching above the base, leafy, terete or weakly compressed, smooth; nodes 3–4, terete or slightly compressed, 1–2 exerted. **Leaf** sheaths slightly compressed, smooth or sparsely to infrequently densely scabrous, glabrous or rarely retrorsely hirtellous to strigulose; butt sheaths papery, smooth, glabrous; flag leaf sheaths (5–)7–18 cm long, margins fused 34–65% the length, fused by an invaginated hyaline margin for much of that length, 1.1–3.1 × longer than its blade; collar margins of lower and mid culm leaves usually flared, smooth or scabrous, glabrous or more often ciliate; ligules 0.8–3 mm long, scarious to hyaline, adaxially scabrous or puberulent, upper margins often ciliolate, apices truncate and entire below to obtuse and irregularly dentate/lacerate above, sterile shoot ligules like those of the culm leaves; blades 2–30 cm long, (1.5–)2–3 mm wide, flat and slightly lax to involute and moderately firm, abaxially and adaxially smooth or lightly scabrous over the veins, margins scabrous, narrowly prow-tipped; lower mid-cauline blades the longest, 10–30 cm long, shorter upward, flag leaf blades 27–50 (90)% longer than their sheaths, flag leaf blade 2.3–10.8 cm long; sterile shoot blades similar to cauline blades or more involute. **Panicles** 6.5–20 cm long, erect, open, usually trapezoidal to pyramidal, sparse to moderately congested, with (18–)40–100 spikelets, proximal internode (2–)3–5 cm long; rachis with (1–)2–4(–5) branches per node; primary branches spreading to eventually reflexed, fairly flexuous, terete to weakly angled, sparsely to moderately scabrous; lateral pedicels 1/2 to equaling the spikelets, moderately to densely scabrous, prickles fine to moderately coarse; longest branches 4–10 cm, with 3–34 spikelets in distal 1/2, loosely arranged. **Spikelets** 3–8 mm long, to 3.5 × long as wide, lanceolate, laterally compressed (sexually dimorphic – staminate spikelets with more florets, up to 7–9); spikelets infrequently bulbiferous; florets (2–)3–6(–9), pistillate, staminate, or hermaphroditic; rachilla internodes terete, distal internodes terete, exceeding 1 mm long, smooth or lightly scabrous, glabrous; glumes narrow lanceolate, distinctly keeled; lower glumes 1.6–3.5 mm long, 1/2–2/3 as long as adjacent lemmas, 1-veined; upper glumes 2.2–4.9 mm long, 3-veined; calluses dorsally webbed, web scant to distinct, hairs 1–2 mm long, woolly; lemmas 2.6–5 mm long, lanceolate, 5-veined, body thinly chartaceous, distinctly keeled, keels to 1/3–2/3 and marginal veins to 1/5–1/3 sparsely short to long villous, intermediate veins smooth or sparsely scabrous, glabrous, between veins smooth, glabrous, intermediate veins obscure to moderately prominent, not extending to near the margin, margins smooth, broadly scarious-hyaline, smooth, apices acute; paleas scabrous, medially rarely softly puberulent over the keels. between keels narrow (0.3–0.4 mm), muriculate, scabrous to sparsely puberulent. **Flowers** chasmogamous; lodicules 0.5 mm long, lanceolate, with a narrow lateral lobe; anthers 1.6–2.8 mm long, sometimes late aborted, infrequently vestigial throughout individual plants. **Caryopses** 1.8–2.1 mm long, elliptical in side-view, sulcus broad and shallow, brown, hilum 0.2 mm long, round, grain adherent to the palea. 2*n* = unknown.

### Distribution.

The species is endemic to the Sierra Madre Oriental and is found in Coahuila and Nuevo León, Mexico.

### Ecology.

This species is found on rocky calcareous substrates in shaded and open forests associated with *Pinus*, *Quercus*, and *Abies*; between 1750−3760 m. Flowering April through October.

### Conservation status.

The species is a regional endemic, known from only 15 collections over 1400 km2.

**Etymology.** The new species is named for Dr. Edward Palmer (1829–1911), an important early collector for the U.S. Department of Agriculture, known particularly for his work in southwestern USA and northern Mexico, who, in 1880, was the first to collect this plant. We selected the *Pringle* collection as type because it is widely distributed.

**Specimen examined.** Mexico. **Coahuila:** mountains 6 mi east of Saltillo, Feb to Oct 1880, E.Palmer 1366 (GH, NY, US-924987⚥). Sierra de Los Lirios, 2800 m, Jul to Aug 1942 [error for 1924?], E.Lyonnet 3691 (MEXU-379081⚥, 379082⚥). 22 km SE of San Antonio de las Alazanas, southeast of Saltillo, 20 Jul 1963, F.W.Gould 10538A & D. Watson (TAES). Rincòn de María, on Hacienda La Babia, ca. 70 road miles northwest from Múzquiz, 1750 m, 27 Apr 1975, T.Wendt 883 & D.Riskind (LL). Cima de Sierra la Viga, ca. 3600 m, 24 Oct 1984, A.McDonald 1172 & Gomez (TAES⚥). ceja y ladera s de Sierra La Viga, 3700 m, 22 Aug 1986, A.McDonald 2104 (TEX, TEX). Las Vigas, Cañón de la Carbonera, Sierra de Arteaga, 2100−2600 m, 5 Jun 1987, J.A.Villarreal & M.A.Carranza s.n. (MEXU-469611). Sierra La Marta, east of Cerro Moro, 3400 m, 22 Jul 1985, S.Ginzbarg 148, A.Whittemore & A.McDonald (MEXU-666069♀). Sierra Coahuilon, ca. 3400−3500 m, 18 Jun 1985, A.McDonald 1485 & M.H.Cervera (MEXU-1072101⚥). SE of San Antonio de las Alazanas, SE of Saltillo, at end of road near Summit of Coahuilon, 25.2203°N, 100.3305°W, 3120 m, 17 Oct 1989, P.M.Peterson 8390, J.Valdes-Reyna, & J.Villarreal (US-3518353⚥). 51.6 km southeast of Saltillo and 13 km southeast of Jame on road to Sierra La Viga, 25.33°N, 100.579°W, 3240 m, 26 Sep 1990, P.M.Peterson 10053, C.R.Annable & J.Valdes-Reyna (US). Sierra Zapalinamé, east of Saltillo, 25.3468°N, 100.9016°W, 2700 m, 2 Sep 2005, P.M.Peterson 18787 & J.Valdes-Reyna (US-3496160 (DNA voucher, unpublished, distributed as “*Poa ruprechtii*"); ditto, E of Saltillo, “El Penitente", 25.3468°N, 100.9016°W, 3070 m, 2 Sep 2005, P.M.Peterson 18790 & J.Valdes-Reyna (US-3496160); ditto, along trail from El Cuatro to El Penitente, 25.3383°N, 100.8882°W, 2925 m, 28 Sep 2007, P.M.Peterson 21127, J.M.Saarela, & S.G.Gómez-Pérez (US; DNA voucher, unpublished). **Nuevo León:** Cerro Potosí, east slope, 2135 m, 9 Jul 1963, R.L.McGregor 385, L.J.Harms, A.J.Robinson, R.delRosario, R.Segal (US-2454930⚥).

### Discussion.

A provisional key to 11 species of *Poa* in northern Mexico (excluding Baja California) is provided in Peterson et al. (2006). In that paper we concluded that *Poa ruprechtii*, as widely applied, was heterogeneous. However, until we could examine the type of *Poa ruprechtii* (*C. Heller 312*) we could not be sure of the application of the name. Here we emend the description of *Poa ruprechtii* Peyr. s.s., and treat the material of northeastern Mexico as a new species. *Poa palmeri* has long been confused with *Poa ruprechtii* (Fournier 1886, Hitchcock 1913, Beetle et al. 1999, Espejo Serna et al. 2000, Dávila Aranda et al. 2006). *Poa palmeri* differs in several leaf, panicle, and spikelet characters, and has longer anthers, and the two species do not overlap in geographic range. There is a note on Palmer’s label at US, “*Poa ruprechtii* Peyr, so named at Kew, S.W. (RJS−presumably Sereno Watson), but scarcely distinct from *Poa flexuosa* Muhl." (= *Poa autumnalis* Muhl. ex Elliott of lowlands from the central and southern Appalachians), and a further note; “related to *Poa flexuosa*" apparently written by A.S. Hitchcock. Interestingly, this name was also applied to the type of *Poa ruprechtii* at W (Fig. 16). This presumably stems from Peyritsch’s final statement (p. 8) that his new species is like what is now called *Poa cuspidata* Nutt. (also of the Appalachians), which has sometimes been confused in herbaria with *Poa autumnalis*. Peyritsch stated, “Scheint mit *Poa brachyphylla* Schult. (*Poa brevifolia* Muhl.) verwant zu sein." The latter two names are synonyms of *Poa cuspidata* Nutt.

Pringle’s label on the type collection can be read as 3000 ft, but we wonder if this is a printing error as the 3 looks more like an 8 when inspected closely, without the serifs of the 3 that are present in the date and other numbers. Whatever the case is, 3000 ft seems far too low for this species.

The breeding system of *Poa palmeri* is not clear. We tentatively identify it as trioecious because a few specimens are staminate throughout, a few are pistillate, most are hermaphroditic, but some of the later have late aborted stamens. Possibly sex-expression varies within some individuals between late and early season panicles, as seen in sequential gynomonoecism (Soreng and Keil 2003).

It is most easily differentiated from *Poa strictiramea* by having smooth foliage, more developed lemma and callus pubescence, and smoother lemma surfaces.

**Figure 15.**
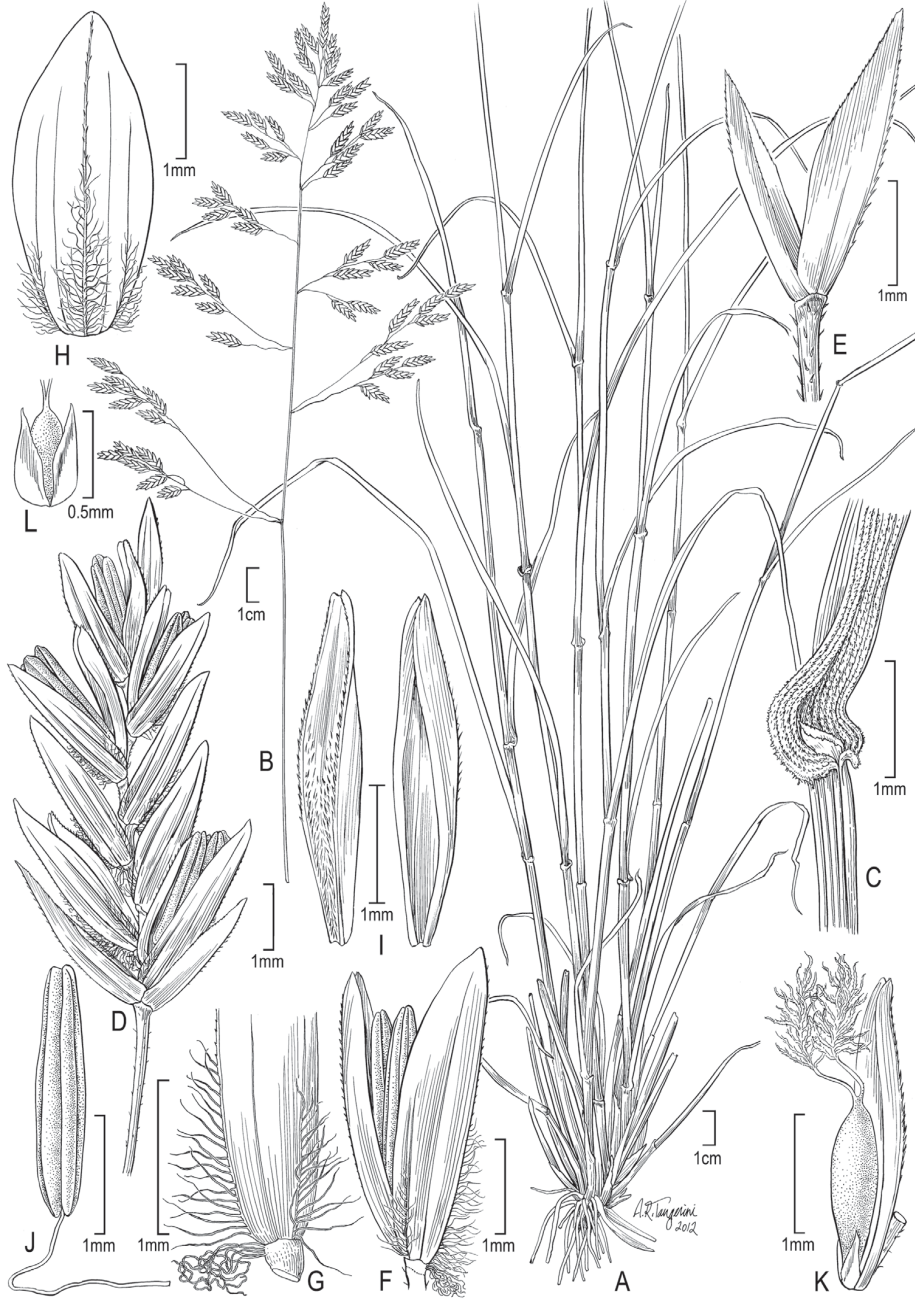
*Poa palmeri* Soreng & P.M. Peterson. **A** habit **B** inflorescence **C** sheath, ligule, blade **D** spikelet **E** glumes **F** floret **G** callus of floret **H** lemma dorsal view **I** palea dorsal and ventral views **J** stamen **K** pistil from perfect-flowered plant, with lodicules attached to palea **L** rudimentary pistil from staminate plant, with lodicules. **A–J** and **L** drawn from holotype collection (*Pringle 10212*)**K** drawn from *Palmer 1366*.

**Figure 16.**
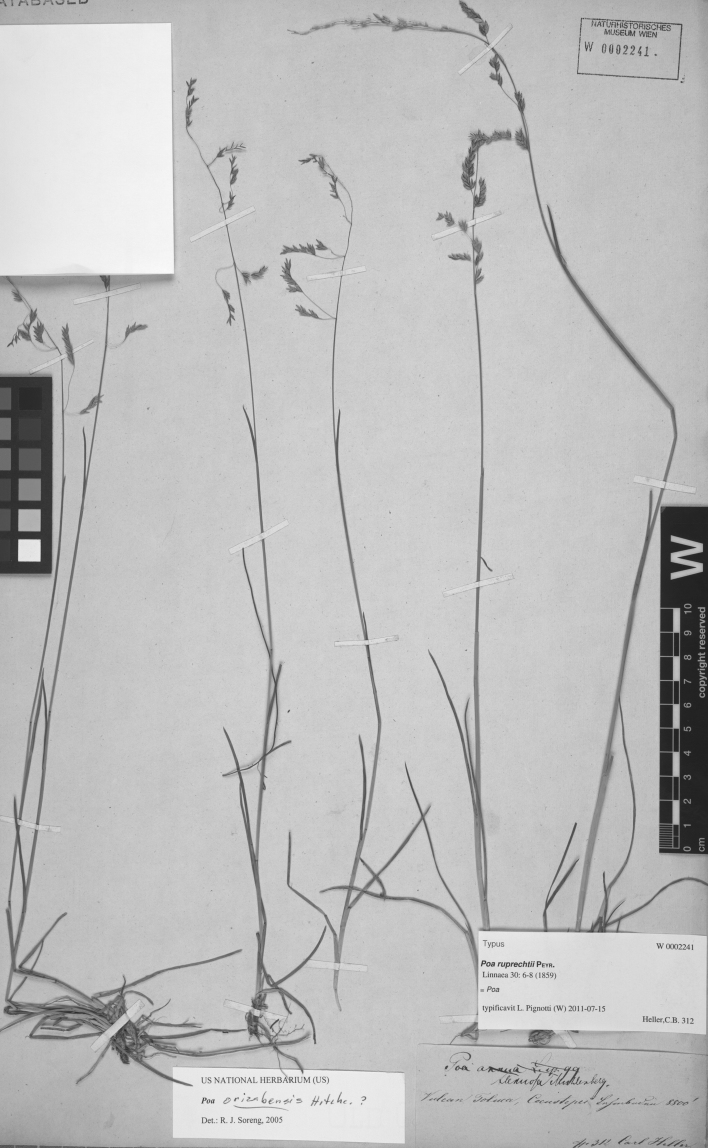
*Poa ruprechtii* Peyr. Photo of holotype collection (*Heller 312*).

## 16. Poa pratensis

L., Sp. Pl. 1: 67–68, 1753.

http://species-id.net/wiki/Poa_pratensis

Fig. 17 18

### Type:

Russia, Prov. Sanct-Petersburg, 5 km australi-occidentum, 26 Jun 1997, *N.N. Tzvelev N-257* (conserved type: BM! designated by Soreng and Barrie 1998, 157; isotypes: B!, C!, CAN!, CONC!, H!, K!, KW!, L!, LE!, LIV!, MA!, MO!, MW!, NSW!, P!, PE!, PR!, S!, SI!, TNS!, US-3456252!, W!).

### Description.

Hermaphroditic. **Perennials**; tufted or not, rhizomatous, shoots solitary or tufted in part, tufts of narrow to medium girth and low to medium height, sometimes forming a dense turf (as in lawns), green, bluish-green, or bluish-gray-green; tillers extravaginal (basally cataphyllous), or also intravaginal (each subtended by a single elongated, 2-keeled, longitudinally split prophyll), with lateral and downward tending, cataphyllous shoots. **Culms** 5–70(–100) cm tall, erect or bases decumbent, leafy, terete or weakly compressed, smooth; nodes terete or weakly compressed, 1–2(–3) nodes exposed, proximal node(s) usually not exerted. **Leaf** sheaths terete to slightly compressed, glabrous or infrequently sparsely to moderately puberulent; butt sheaths papery, smooth, glabrous; flag leaf sheaths 2–20 cm long, margins fused 25–50% the length, 1.2–5(–6.2) × long as its blade; collars smooth, glabrous or ciliate along the margins; ligules 0.9–2(–3.1) mm long, abaxially smooth or scabrous, upper margin ciliolate or glabrous, apices truncate to rounded, infrequently obtuse; blades of cauline leaves 0.4–4.5 mm wide, flat or folded, to involute on the margins, soft and lax to moderately firm, abaxially smooth, glabrous, adaxially smooth or lightly scabrous, frequently with sparse, slender, erect to appressed, curving, sinuous or strait hairs to 0.2–0.8 mm long, broadly prow-tipped or some narrowly prow-tipped; blades subequal, or middle blades longest, flag leaf blades 1.5–10 cm; sterile shoot blades of extravaginal shoots like those of the culm, of intravaginal shoots, when present, sometimes distinctly narrower (0.4–1 mm wide), flat to involute. **Panicles** 2–15(–20) cm long, erect or nodding, loosely contacted to open, narrowly ovoid to narrowly or broadly pyramidal, sparse to moderately congested, with (25–)30 to over 100 spikelets; rachis with (1–)2–7(–9) branches per node; primary branches spreading early or late, terete or angled, smooth or sparsely to moderately densely scabrous; lateral pedicels usually 1/5–1/2 the spikelet in length, smooth, or sparsely to moderately densely scabrous, prickles fairly fine; longest branches (1–)2–9 cm, with 4–30(–50) spikelets, spikelets usually fairly crowded in distal 1/2. **Spikelets** 3.5–6(–7) mm long, to 3.5 × long as wide, lanceolate to ovate, laterally compressed, sometimes bulbiferous; florets 2–5, infrequently bulbous basally and leaf-like distally, hermaphroditic; rachilla internodes terete, mostly less than 1 mm long, smooth, glabrous; glumes narrowly lanceolate to lanceolate, infrequently broadly lanceolate, unequal to subequal, usually distinctly shorter than the adjacent lemmas, distinctly keeled, keels sparsely to densely, usually moderately, scabrous, infrequently smooth, apex acute, to acuminate; lower glumes 1.5–4(–4.5) mm long, 1–3-veined, narrowly lanceolate, occasionally weakly sickle shaped; upper glumes 2–4.5(–5) mm long, distinctly shorter to nearly equaling lowest lemma, 3-veined; calluses dorsally webbed, web well developed, sometimes with secondary tufts under each marginal vein, hairs 2–4 mm long, woolly; lemmas 2–4.3(–6) mm long, lanceolate, green or strongly purple colored, distinctly keeled, keels and marginal veins long villous, intermediate veins glabrous or infrequently short villous to softly puberulent, between veins glabrous, smooth or finely muriculate, smooth or lightly scabrous above, intermediate veins prominent, margins narrowly to broadly hyaline, apices acute; paleas scabrous, medially sometimes softly puberulent over the keels, intercostal region narrow, glabrous, or rarely very sparsely and minutely hispidulous. **Flowers** chasmogamous; lodicules 0.35–0.5(–0.7) mm long, broadly ovate, with a short lateral lobe about midpoint; anthers 1.2–2 mm long, infrequently aborted late in development and ca. 1 mm long. **Caryopses** 1.5–2 mm long, elliptical in side-view, slightly laterally compressed, subtrigonous in cross-section, brown, sulcus broad, shallow, hilum 0.2 mm long, round to oval, grain adherent to the palea. 2*n* = 28–147.

### Distribution.

The species is distributed worldwide but absent from tropical countries except in high mountains, or where introduced. In Mexico, the species is known from Baja California, Chihuahua, Coahuila, Distrito Federal, Hidalgo, Mexico, Nuevo León, San Luis Potosí and Veracruz. *Poa pratensis* subsp. *agassizensis* and subsp. *alpigena* are possibly native to Mexico.

### Ecology.

The facultatively apomictic, mostly high polyploid species inhabits cool mesic to frigid climates, is often seeded for pastures and lawns, and is easily established outside of cultivation since it tolerates disturbance. In Mexico, it occurs from 10–3650 m. Flowering May to July.

### Discussion.

Even though the species is highly plastic and tends to look a bit odd in low latitudes, we made an attempt to sort out the subspecific forms in Mexico. The results were unsatisfactory. We have identified a few specimens that match the typical forms, but we could not confidently place most of the material into subspecies. *Poa pratensis* is primarily a high polyploid and facultatively apomictic (Clausen 1961). It is a common circumboreal species with numerous strains that are treated as species by some authors and as subspecies by others. In Russia (including the former Soviet States) the decision of whether to recognize the various morphological “forms" as subspecies or as distinct species has changed (Tzvelev 1976, Czerepanov 1995) in favor of species, while in the UK Cope and Gray (2009) in the United Kingdom, and Portal (2005) in France Belgium and Switzerland have gone with subspecies. Stoneberg-Holt (2004) correlated morphology and ploidy-level in samples of *Poa pratensis* collected mainly across Eastern Europe and in Montana in the USA, and grown in a common garden with and without shade. She concluded that there was a continuum of morphological forms that grade from one extreme to another. Plants with predominantly very-fine, moderately firm (form retaining) intravaginal-leaved shoots, and low polyploidy (2n = 28–42) are referable to subsp. *angustifolia*; these grade into plants with some intravaginal-leaved shoots that are mostly soft-bladed and mainly of middle-ploidy (2n = 42–56) that are referable to subsp. *pratensis*; these grade into plants with all or most shoots extravaginal, fairly broad-bladed, of mainly higher ploidy (2n = 58–144) referable to subsp. *irrigata*. Selections by plant breeders from across the range of these forms are all evidently introduced into North America for pastures, soil stabilization, and lawns. However, the cultivated forms have been selected from forms attractive for lawns and most durable to mowing and trampling, and we are no longer dealing with geographic and ecologically differentiated natural taxa. *Poa pratensis* subsp. *alpigena* and subsp. *agassizensis* are probably native and are primarily mid-range polyploids. Our key to subspecies is presented for heuristic purposes; however, in practice it is difficult to draw a firm line between the taxa.

**Figure 17.**
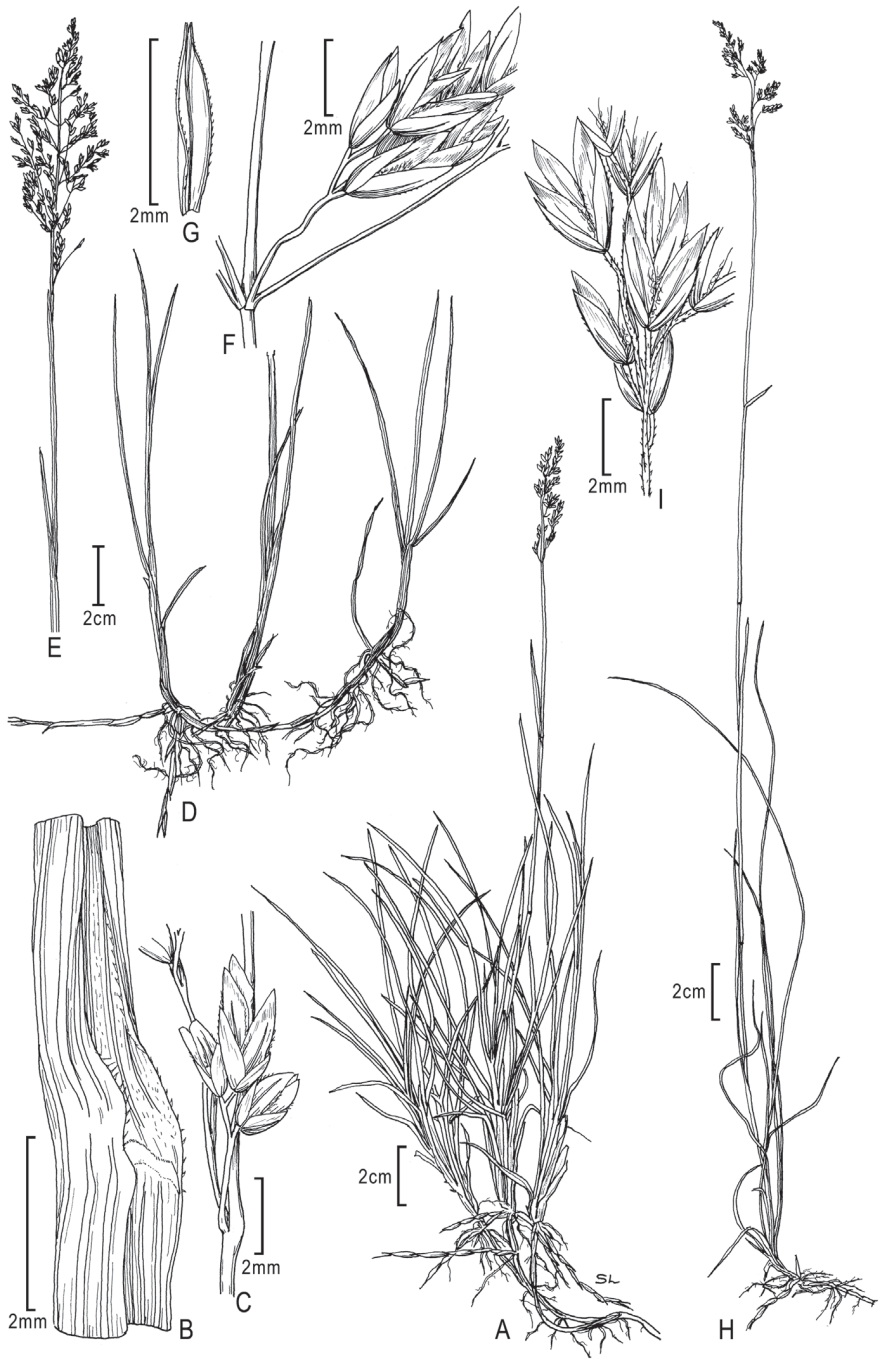
*Poa pratensis*L. **A–C** *Poa pratensis* subsp. *agassizensis* (B. Boivin & D. Löve) Roy L. Taylor & MacBryde **A** habit **B** sheath, ligule, blade lateral view **C** branch segment with spikelets **D–G** subsp. *alpigena* (Lindm.) Hiitonen **D** habit **E** inflorescence **F** branch segment with spikelets **G** palea **H, I** subsp. *angustifolia* (L.) Lej. **H** habit **I** branch segment with spikelets. Drawings from Soreng (2007).

**Figure 18.**
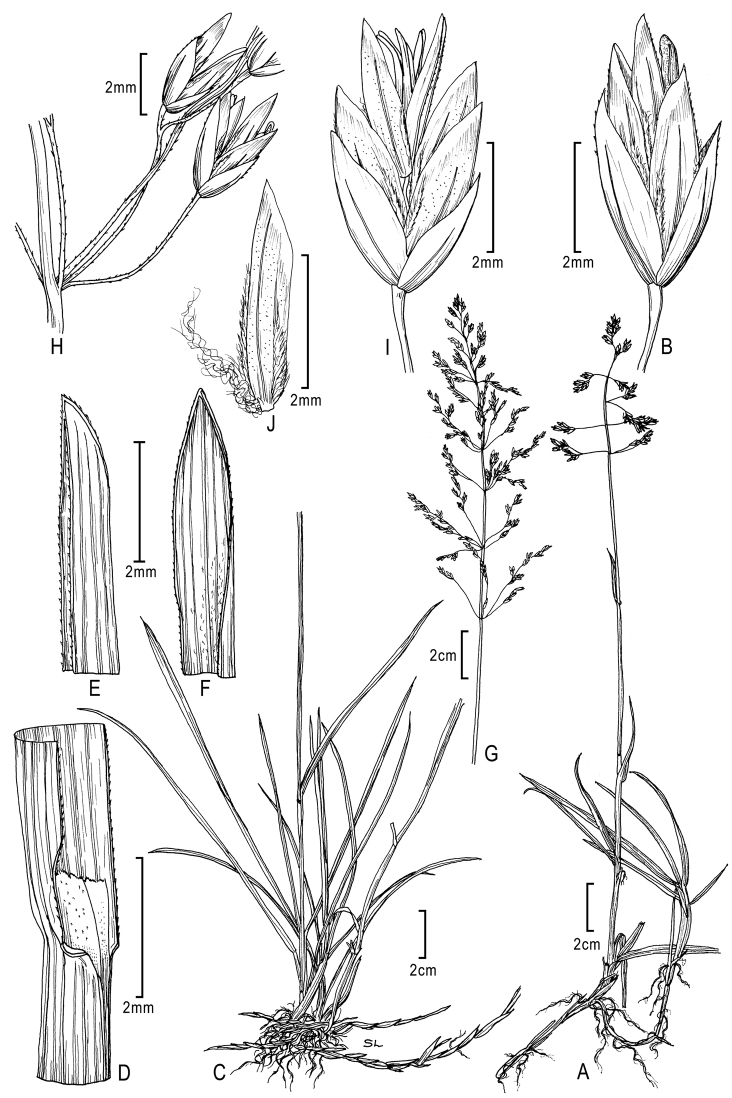
*Poa pratensis*L. **A, B** *Poa pratensis* subsp. *irrigata* (Lindm.)H. Lindb. **A** habit **B** spikelet **C–J** **subsp. *pratensis* **C** habit **D** sheath, ligule blade lateral view **E** blade apex lateral view **F** blade apex adaxial view **G** inflorescence **H** branch segment with spikelets **I** spikelet **J** floret. Drawings from Soreng (2007).

### Key to the subspecies of *Poa pratensis*

**Table:** 

1	Vegetative shoots intravaginal (each with a well-developed, 2-keeled, longitudinally-spilt prophyll), and extravaginal shoots (each with a rudimentary prophyll and one to several cataphylls at the base); the involute blades often distinctly narrower than the flat ones	2
–	Vegetative shoots all extravaginal (or infrequently also with an intravaginal shoot), isolated or crowded (each extravaginal shoot with a rudimentary prophyll, and one to several cataphylls at the base)	4
2	Blades mostly flat and fairly soft, or a mixture of folded-involute moderately-soft to soft vegetative shoot blades, and flat or folded, soft culm blades; the adaxial surfaces of all blades usually glabrous; abaxial veins of intravaginal shoot blades slender, narrower than the intercostal surfaces; plants of pastures and parks and waste ground to subalpine	6e. *Poa pratensis* subsp. *pratensis*
–	Blades of vegetative and culm shoots involute on the margins, moderately firm, and of fairly uniform width; the adaxial surfaces, at least of the vegetative shoot blades with sparse, elongated, weakly-appressed hairs; abaxial veins of intravaginal shoot blades slender to thick, narrower to broader than the intercostal surfaces; plants widespread	3
3	Blades ca. (0.8–) 1–2 mm wide (expanded), the longer vegetative shoot blades short less than 10 (–15) long; panicles contracted, branches smooth or sparsely scabrous; plants of mountain meadows, open forests, and subalpine	16a. *Poa pratensis* subsp. *agassizensis*
–	Blades 0.4–1 mm wide (expanded), the longer vegetative shoot blades often exceeding 10 cm long; panicles loosely contracted to open, branches usually scabrous; plants of pastures, waste ground	16c. *Poa pratensis* subsp. *angustifolia*
4	Extravaginal shoot blades fairly slender (1–2 mm wide), usually folded; vegetative shoots mostly isolated; panicles narrowly pyramidal, branches mostly smooth or sparsely scabrous, spikelets 3–4.5 mm long ; plants of subalpine to alpine	16b. *Poa pratensis* subsp. *alpigen*a
–	Extravaginal shoot blades generally broader (2–5 mm wide), flat or folded, often lax; vegetative shoots isolated or crowded; panicles pyramidal to broadly pyramidal, branches somewhat sparsely to fairly densely scabrous; spikelets 4–7 mm long; plants of lawns and parks and waste ground to low alpine	5
5	Glumes subequal, frequently pruinose, lower glumes (1) 3-viened, similar in shape to the upper glume, upper glumes often equal to the lowest lemma in length; panicles fairly sparsely flowered; collar margins and ligule abaxial surfaces commonly pubescent	16d. *Poa pratensis* subsp. *irrigata*
–	Glumes unequal, infrequently pruinose, lower glumes 1 (3)-veined, narrower than the upper glume, upper glumes usually shorter than lowest lemma; panicles moderately densely flowered; collar margins and ligule abaxial surfaces usually glabrous	16e. *Poa pratensis* subsp. *pratensis*

## 16a. Poa pratensis agassizensis

(B. Boivin & D. Löve) Roy L. Taylor & MacBryde, Canad. J. Bot. 56(2): 193. 1978.

http://species-id.net/wiki/Poa_pratensis_agassizensis

Fig. 17 A–C

Poa agassizensis B. Boivin & D. Löve, Naturaliste Canad. 87(6–7): 176–180, f. 1–2. 1960. Type: Canada, Manitoba, MacDonald Co, 6 Jun 1952, *B.Boivin, Löve & Alex 9167* (holotype DAO!; isotype: US-2553819!).

### Description.

Tufts sparse, some shoots clustered; pale green or bluish-gray-green; tillers intra- and extravaginal. **Culms** 20–40(–50) cm tall. **Ligules** of lower culm and tiller leaves commonly glabrous abaxially; lades of cauline leaves flag leaf blades folded, with involute margins, moderately thick, moderately firm; sterile shoot blades usually less than 10 cm long, 0.8–2 mm wide, all folded with involute margins, sparsely pubescent adaxially. **Panicles** 4–6(–8) cm long, erect or nodding, or loosely contracted or open, ovoid to narrowly pyramidal; rachis with 2–3(–5) branches per node; primary branches steeply ascending to ascending, smooth or sparsely to moderately densely scabrous; longest 1–2.5(–3) cm, with several spikelets per branch. **Spikelets** lanceolate, not bulbiferous; glumes unequal, glaucous or not; lower glumes 1(–3)-veined; upper glumes shorter than or nearly subequaling the lowest lemma; lemmas 2–3(–3.5) mm long, finely muriculate, intermediate veins glabrous; paleas scabrous, medially glabrous over the keels, intercostal region glabrous. **Anthers** frequently sterile. 2*n* = 41, 42, 43, 56.

### Distribution.

This subspecies in North America is known from Canada and USA, and in Mexico from the states of Baja California and Coahuila.

### Ecology.

The subspecies inhabits cool temperate prairies, meadows, and open coniferous forests.

### Specimens examined.

Mexico. **Baja California:** Municipio Ensenada, Paraje El Rayo Ejido, 1000 m, 13 May 1997, A.Miranda 1153 (MEXU). Sierra San Pedro Mártir, La Gurulla, 30°54'N, 115°29'W, 2050 m, 29 Jun 1982, R.Moran 30956 (MEXU, TAES). **Coahuila:** Sierra de Arteaga, Las Vigas, Canon de la Carbonera, 25°20'N, 100°39'W, 2100 m, 5 Jun 1987, J.A.Villarreal 3800 & M.A.Carranza (TAES). Municipio Arteaga, Ejido La Escondida, 25°23'30"N, 100°33'15"W, J.A.Garcia 49 (MEXU).

### Discussion.

This form of *Poa pratensis*, originally described as a species indigenous to Canada and the United States (Boivin and Löve 1960) and now accepted as *Poa pratensis* subsp. *agassizensis*, is probably indigenous, but morphologically is questionably distinct from the Eurasian elements of *Poa pratensis* subsp. *angustifolia*, or xeric forms of cultivated *Poa pratensis* subsp. *pratensis* (Soreng 2007). As originally described it is a taxon with short, ovoid panicles, with small spikelets, and involute leaves. Such specimens are not uncommon among early herbarium collections across Canada and the USA. It approaches *Poa pratensis* subsp. *angustifolia* closely, but that subspecies of Eurasian origin has looser panicles, and is generally a lower polyploid (Stoneberg-Holt 2004). Separation of *Poa pratensis* subsp. *agassizensis* from *Poa pratensis* subsp. *pratensis* is difficult, and so far there is no molecular or cytological data to support it as a separate taxon. We recognize *Poa pratensis* subsp. *agassizensis* as a marginally distinct taxon, and site a few vouchers that best match this form.

## 16b. Poa pratensis alpigena

(Lindm.) Hiitonen, Suom. Kasvio 205. 1933.

http://species-id.net/wiki/Poa_pratensis_alpigena

Fig. 17 D–G

Poa alpigena Lindm., Sv. Fanerogamfl. 91. 1918. *Poa pratensis* var. *alpigena* Fr., Herb. Norm. 9: 93. 1842, nom. illeg. superfl. *Poa pratensis* var. *alpigena* Fr. ex Blytt., Norges Fl. 1: 130. 1861, nom. illeg. superfl. *Poa pratensis* var. *iantha* Laest., Kongl. Vetensk. Acad. Handl. 1822: 329. 1822. Type: Scandinavia: in insula Gammelgarden et Rosbacken juxta Quickjock, Lule Lappmark, *Laestedius* s.n. (syntypes: S-6651, S-6670, UPS-3 (herb. Hartmann), UPS-4 (herb. Wahlenberg).
Poa oligeria Steud., Syn. Pl. Glumac. 1: 426. 1854. Type: Chile: Sandy Point Magellan, Dec, *W.Lechler 1192* (isotypes: LE!, S-03-2215!, US-81727! ex W, US-946978! fragm. ex LE, W-243018!).

### Description.

Tufts sparse, or all shoots solitary; green (often anthocyanic) tillers mainly extravaginal. **Culms** 15–70 cm tall. **Blades** of cauline leaves flag leaf blades usually folded, thin, soft; sterile shoot blades less than 15 cm long, 1–3.6 mm wide, flat or folded, usually sparsely pubescent adaxially. **Panicles** 3–13(–20) cm long, contracted or narrowly pyramidal, expanding well after emergence from the sheath; rachis with (1–)2–6(–7) branches per node; primary branches steeply ascending to eventually spreading or somewhat reflexed, smooth or lightly scabrous; longest branches 1–6 cm, with 5–15 spikelets. **Spikelets** 4–6.5 mm long, narrowly lanceolate, not bulbiferous; glumes unequal, glaucous or not; lower glumes 1–3-veined; upper glumes subequaling the lowest lemma; lemmas 2.5–3.5 mm long, smooth or finely muriculate, intermediate veins frequently sparsely to moderately densely short villous; paleas scabrous, medially frequently softly puberulent over the keels, intercostal region glabrous, or rarely sparsely hispidulous. 2*n* = 28, 32, 35, 42, 43, 48, 50, 52, 53, 56, 60, 62, 63, 64, 67–82, 84, 86, 88, 89, 92, ca. 94, ca. 124, 127.

### Distribution.

The subspecies is circumboreal and in North America occurs in Canada, Greenland, USA, and Mexico (Nuevo León).

### Ecology.

This subspecies is found in boreal to alpine forests, and it tolerates frigid conditions.

### Conservation status.

This native subspecies is common across Canada, and uncommon in the Rocky Mountains south of Colorado, and locally uncommon in Mexico.

### Specimens examined.

Mexico. **Nuevo León:** localizado en Galeana, Cerro el Potosi, 20°52'23"N, 100°13'48"W, 3650 m, 15 Aug 1998, *Ing.M.Castillo-B. 345 & Ing.J.Garza-C*. (MEXU-1072117 & MEXU 999120, p.p. “a", p.p. “b" is *Poa mulleri*, on both sheets, fide RJS).

### Discussion.

*Poa pratensis* subsp. *alpigena* is circumboreal and native to the New World in the arctic, alpine, subalpine, and boreal forests (Soreng 2007). This subspecies is recognizable in plastid DNA data (due to a deletion in *trn-TLF*) and nrDNA sequences as distinct from other subspecies so far evaluated (Gillespie et al. 2005, 2007). In the lower 48 states of USA, *Poa pratensis* subsp. *alpigena* occurs at scattered locations as far south as northern Arizona and New Mexico in alpine and subalpine habitats. This subspecies also occurs in Tierra del Fuego, Chile (see type of *Poa oligeria*). This is the first report of this subspecies for Mexico, where it is probably best considered an interglacial period relict from glacial expansions. It can be separated from the other subspecies by its loose rhizomatous habit, small spikelets, narrow leaves, and narrow (often only slightly spreading) panicles. In North America, it usually has some scant hairs on the intermediate veins of the lemmas and on the palea keels. Several other specimens, particularly from Orizaba, approach this subspecies (see those under subsp. *pratensis* annotated as “toward *alpigena*").

## 16c. Poa pratensis angustifolia

(L.) Lej., Comp. Fl. Belg. 82. 1828.

http://species-id.net/wiki/Poa_pratensis_angustifolia

Fig. 17 H, I

Poa angustifolia L., Sp. Pl. 1: 67. 1753. (lectotype: LINN-87.12!, excluding second culm from the left, designated by Soreng 2000: 254).

### Description.

Tufts sparse to dense, some shoots clustered; pale green or bluish-gray-green; tillers intra- and extravaginal. **Culms** 25–80 cm tall. **Ligules** of lower culm and tiller leaves commonly glabrous abaxially; blades of cauline leaves flag leaf blades folded or involute, with involute margins, moderately thick or thin, moderately thin or soft; sterile shoot blades 10–45 cm long, 0.4–1 mm wide, all involute, like or often distinctly narrower than cauline blades, sparsely pubescent adaxially. **Panicles** 8–18 cm long, loosely contracted, or open and narrowly pyramidal; rachis with 3–6 branches per node; primary branches ascending to spreading, smooth, or sparsely to densely scabrous; spikelets several to many per branch. **Spikelets** narrowly lanceolate, not bulbiferous; glumes unequal, infrequently glaucous; lower glumes 1(–3)-veined; upper glumes shorter than or subequaling the lowest lemma; lemmas 2.5–3.5 mm long, finely muriculate, intermediate veins glabrous; paleas scabrous, medially glabrous over the keels, intercostal region glabrous. 2*n* = 28, 46, 48, 49–54, 56, 57, 59–66, 72.

### Distribution.

This subspecies is native to Eurasia. It is introduced in North America where it is known from Canada, USA, and in Mexico (San Luis Potosí).

### Ecology.

The subspecies isintroduced and sometimes is included in pasture grass seed mixes, it tolerates drought better than other subspecies except perhaps subsp. *agassizensis*.

### Specimens examined.

Mexico. **San Luis Potosí:** 25 mi E of San Luis Potosí on highway 70, 27 May 1979, F.W.Gould 15603 (TAES).

### Discussion.

This *Poa pratensis* subspecies is more drought tolerant than the others, except perhaps subsp. *agassizensis*. It is most easily recognized by its very fine, relatively firm, involute leaf blades that are adaxially pubescent. This subspecies name is often applied to collections of subsp. *pratensis*. The latter often has narrow intravaginal leaves but those are softer and adaxially glabrous. According to Stoneberg-Holt’s (2004) results, subsp. *angustifolia* is lower polyploid, and many of the higher counts reported in the literature for this taxon (at least those above 2*n* = 56) are possibly referable to subsp. *pratensis*.

## 16d. Poa pratensis irrigata

(Lindm.) H. Lindb., Sched. Pl. Findland. Exs. 2: 20. 1916.

http://species-id.net/wiki/Poa_pratensis_irrigata

Fig. 18 A, B

Poa humilis Ehrh. ex Hoffm., Deutschland Flora 1: 45. 1800. Type: Sweden, Uppsala, Ehrhart 115 (isotypes: LE! plant B on sheet, plant A on sheet is *Poa pratensis* subsp. *alpigena*, LE! [2 sheets] plant B ex E. Fries Herb. Normal, LE-TRIN-2598.02! plant B ex E. Fries Herb. Normal).
Poa bourgeaei E. Fourn. Mexic. Pl. 2: 113 1886. Type: Mexico, Distrito Federal, pres San Angel, 23 May 1865, *E. Bourgeau 225* (isotype: US-89690! fragm.).

### Description.

Tufts sparse or dense to loose, sometimes forming turf, or some or all shoots solitary; dark green, or bluish-gray-green; tillers mainly extravaginal. **Culms** 8–30(–50) cm tall. collar margins commonly retroresly strigulose. **Ligules** of lower culm and tiller leaves commonly pubescent abaxially; blades of cauline leaves flag leaf blades flat, thin, soft; sterile shoot blades usually less than 15 cm long, 2–4.5 mm wide, usually glabrous adaxially. **Panicles** 2–10 cm long, open, broadly pyramidal; rachis with 1–3(–5) branches per node; primary branches widely spreading, smooth or sparsely to moderately scabrous; longest branches 1.5–6 cm, with 4–8 spikelets. **Spikelets** lanceolate to broadly lanceolate, not bulbiferous; glumes subequal, often glaucous; lower glumes (1–)3-veined; upper glumes usually subequaling the lowest lemma; lemmas 3–6(–6) mm long, finely muriculate, intermediate veins glabrous; paleas scabrous, medially glabrous over the keels, intercostal region glabrous. 2*n =* 54, 56, 65, 80, 82–147.

### Distribution.

The subspecies occurs in Eurasia, North America (Canada, Greenland, USA, and Mexico (Veracruz).

### Ecology.

The introduced subspecies iscultivated as a turf grass in mesic, cool temperate regions.

### Specimens examined.

Mexico. **Veracruz:** Municipio Perote, Escobillo, 19°31'30"N, 97°13'W, 3000 m, Mar 1991, H.R.Sandoval 78 (MEXU).

### Discussion.

The subspecies is often cultivated for pastures and lawns and many of the cultivars originate from Eurasian selections, or plants selected from foreign strains established in North America; and cultivated strains are certainly present in Mexico (see type of *Poa bourgeaei*). Of more than 700 chromosome counts RJS has compiled from the literature for this taxon the vast majority are between 2*n =* 80 and 147. Cultivated forms selected for lawns with soft flat leaves and loose tufts have generally been referred to *Poa pratensis* subsp. *irrigata*, which is considered Eurasian in origin. Some authors suggest *Poa pratensis* subsp. *latifolia* (Weihe ex Mert. & W.D.J.Koch) Schübl. & G.Martens is the same taxon and is the correct name (Portal 2005). At the species rank this subspecies has been called *Poa humilis* Ehrh. ex Hoffm. and *Poa subcaerulea* Sm. *Poa pratensis* is possibly the World’ s most complex species, fascinating in itself, but of which we know both much and too little.

## 16e. Poa pratensis pratensis

http://species-id.net/wiki/Poa_pratensis_pratensis

Fig. 18 C–J

### Description.

Tufts sparse, or dense to loose, shoots clustered, or some solitary; green, or bluish-gray-green; tillers intra- and extravaginal. **Culms** 8–100 cm tall. **Ligules** of lower culm and tiller leaves commonly glabrous abaxially; blades of cauline leaves flag leaf blades flat or folded, thin, soft; sterile shoot blades 10–45 cm long, 0.4–4 mm wide, some distinctly narrower than the cauline blades, all flat or some involute, usually glabrous adaxially. **Panicles** 5–18 cm long, loosely contracted or open and broadly pyramidal; rachis with 3–6(–9) branches per node; primary branches spreading to somewhat reflexed, smooth or sparsely to fairly densely scabrous; spikelets several to many per branch. **Spikelets** lanceolate to broadly lanceolate, not bulbiferous; glumes unequal to subequal, infrequently glaucous; lower glumes 1–3-veined; upper glumes shorter than or nearly subequaling the lowest lemma; lemmas 2.8–4.3 mm long, finely muriculate, intermediate veins glabrous; paleas scabrous, medially glabrous over the keels, intercostal region glabrous. **Anthers** sometimes sterile. 2*n* = 41–44, 48–56, 58, 59, 60 ca, 62, 64–70,74, 86–91, 95, 98.

### Distribution.

The subspecies occurs in Eurasia, North America (Canada, Greenland, USA), and Mexico (Baja California, Chihuahua, Coahuila, Distrito Federal, Hidalgo, Mexico, Nuevo León, Veracruz).

### Ecology.

This introduced subspecies occurs mostly between 900–3500 m, in cool temperate habitats, probably in large part due to seeding for soil stabilization, pastures and lawns. Flowering May to July.

### Specimens examined.

Mexico. **Baja California:** Municipio Ensenada, carretera a Tecate, coastal, 10 m, 23 Jun 1979, J.Sánchez 14 (MEXU). Rancho Don Faustina, 3 May 1981, R.Guzmán 1371 (MEXU). Sierra Juárez, Arroyo el Zauz, Laguna Hansen 32°00'N, 115°57'W, 1560 m, 13 May 1997, A.Miranda 574 (MEXU). Rancho Paraiso, km 153 carretera Ensenada−San Felipe, 31°21.305'N, 115°27.209'W, 900 m, 16 May 1997, M.A.Vergara-B. 100 (MEXU). Sierra San Pedro Mártir, Rancho Melling, 7 May 1981, C.Aguirre 258 & V.Morales (MEXU). **Chihuahua:** Municipio de Balleza, camino Balleza-Guachochic, km 45, 24 Sep 1981, I.Flavio-A. 1585, Rafael-F. and M.E.Siquerios (MEXU).Municipio de Madera, ejido El Largo, Rancho de la Ciénega, 2240 m, 24 Jul 1990, A.Benítez 1401 (MEXU). **Coahuila:** Municipio Arteaga, Cañon Jamé, ca. 20 km al del poblado de Jamé; 25°22'N, 100°35'W, 2200 m, J.A.Villarreal 5885 & M.A.Carranza (TEX); ditto, Sierra de la Marta, La Siberia, ca del Ejido Santa Rita, ca. 6 km al southeast de San Antonio de las Alanzanas, 25.2203°12'N, 100°30'W, 2300 m, 27 May 1982, J.A.Villarreal 1687 (TEX). Sierra de Arteaga, de San Antonio de las Alanzanas a Sierra la Marta y porrero de Abrego, 25°25'N, 100°40'W, 2300 m, 20 Jun 1984, J.Valdes-R. 1664 & M.E.Demesa (TEX). **Distrito Federal:** Region Contreras, volcani, 2700 m, 5 May 1926, M.St.Pierre s.n. (US). Canada de Contreras, ca. de Primer Dinamo, 2550 m, 16 Jun 1970, J.Rzedowski 27216 (MEXU, US). Pedregal de San Angel, SE del Pedregal, 16 May 1952, J.Rzedowski 1002 (US). Municipio De Chalco, faldas del Cerro Telapón, 3200 m, 16 Jul 1964, J.Rzedowski 18460 (MEXU). alrededores de la estacion La Cima, delegacion de Tlalpan, 3000 m, 8 Jul 1979, J.Rzedowski 36171(MEXU, TAES). 5 km al W del Desierto de los Leónes sobre el camino a La Cieneguilla, 3250 m, 5 Jun 1983, J.Rzedowski 38086 (MEXU). **Hidalgo:** Municipio de Epazoyucan, ca. de Peñas Largas, 2900 m, 3 Aug 1975, J.Rzedowski 33439 (TAES, toward *alpigena*). Municipio Mineral del Chico, 2 km de Llanos Grandes por camino a Peña del Cuervo, 2900 m, 13 Jun 1992, M.González-Ledesma 502 & J.A.Pérez-de-la-Rosa (MEXU). **Mexico:** Municipio Ixtapaluca, Estacion Exper. Forestal de Zoquiapan, 3300 m, Jul 1976, C.Obieta-O. 91 (MEXU).Sierra del Ajusco, Rancho Alegre, delegacin Tlalpan, 3400 m, 29 Nov 1981, R.C.Moreno-C. 83 (TAES, hybrid?). **Mexico:** A 7 km de Buenavista, a Temascaltepec, 3250 m, 29 Aug 1983, Manrique 282, Jarmillo & Guerrero (MEXU). EEFZ, Cercanias as Cerro El Papayo, 3250 m, 16 May 1997, E.Guízar s.n. et al. (MEXU). 4 km del camino de terraceria hacia el campo EEFZ, 3330 m, 29 Jul 1998, B.Nava-Roberto-C. s.n. (MEXU). Río Frío, 3000 m, 19 Apr 1997, O.Ruiz-N. (MEXU). ca. Paso de Cortés, entre Popocatépetl e Ixtaccíhuatl, 3650 m, 31 Jul 1966, J.Rzedowski 22841 (TAES, appear to be intermediate to *Poa orizabensis*). Municipio Tlalmanalco, Vertiente occidental del Volcán Ixtaccihuatl, 8 km al se de San Rafael, 3125 m, 6 Apr 1997, S.D.Koch 7917 & I.Sanchez-V. (MEXU, MO); ditto, C.Azudia 44 (MO, MEXU). Bosque de San Rafael, 10 May 1971, E.Matuda 38360 (MEXU). Municipio Ixtapaluca, Llano Pinahua, 8 km al southwest de Río Frío, 3200 m, 24 May 1981, J.Rzedowski 37286 (TAES).Municipio Mineral del Chico, Las Ventanas, 2960 m, 19 May 1993, J.Praxedes 49, Pérez,Jiménez (MEXU). **Nuevo León:** Areas cercanas a Cola de Caballo, 25°23'N, 100°10'W, 800 m, 30 May 1987, J.A.Villarreal 3691 & M.A.Carranza (TAES). **Veracruz:** Municipio Chiconquiaco, La Guacamaya, 1900 m, 26 Mar 1975, F.Ventura-A. 11133 (MEXU, TAES). Municipio Perote−Perote, Jun 1893, E.W.Nelson 19 (US). camino entre Perote y Cofre el Perote, 2950 m, 24 Apr 1982, R.Guzmán 4980 (MEXU). camino a El Conejo, 19°31'42"N, 97°09'21"W, 3500 m, 3 Jun 1994, C.R.Galindo 63 (MEXU). mountains near Jalapa, 5000−7000 ft [1525−2135 m], 20 Apr–20 May 1899, C.G.Pringle 7880 (US). Lomogrande, Mt. Orizaba, 8900 ft [2715 m] 28 Apr 1938, E.K.Balls 4382 (US). Municipio Rafael Ramírez, comunidad Los Paisanos, 19°34’ 12"N, 97°05’ 25"W, 28 Mar 1995, P.J.Parroquín 79 (MEXU). Barranca Seca, ca. de La Desviacíon a Villa Aldama, 2350 m, 8 Aug 1970, F.Ventura-A. 1993 (MO, toward *alpigena* or *irrigata*). Cofre de Perote, 3300 m, Apr 1987, B.Veracruz 251 (MEXU, toward *alpigena*). Cofre de Perote, east side, 3930 m, 6 Aug 1958,J.H.Beaman 2179 (MEXU, TEX, US, toward *alpigena*). Cruz Blanca, 8000–10000 ft [2440−3050 m], 21 May 1950, J.T.Baldwin Jr.14281(US, toward *alpigena*).

### Discussion.

This subspecies is commonly included in cool temperate pasture and lawn grass seed mixes. More than 140 of the 162 chromosome counts RJS has compiled from the literature for this taxon are between 2*n* = 50 and 78. The collection of *J.Rzedowski 22841* seems a bit intermediate between this subspecies and *Poa orizabensis*, since it has dense, scabrous branches, lightly pubescent lemma keels and marginal veins, and slightly pointed ligules. The collection of *R.C.Moreno-C. 83* is odd for its dense low tuft, with short, flat blades, lack of rhizomes, and smooth branches, combined with large spikelets, and may represent another inter-species hybrid.

## 17. Poa ruprechtii

Peyr., Linnaea 30(1): 6–8. 1859.

http://species-id.net/wiki/Poa_ruprechtii

Figs 13 P–R 16

### Type:

Mexico, Toluca, Cocustepec, Volcán Toluca, 8800 ft [2440 m], *Carl Heller 312*. (holotype: W-0002241!). *Poa sharpii* Swallen, Contr. U.S. Natl. Herb. 29(9): 400. 1950. Type: Mexico, Veracruz, moist shaded soil near El Puerto, 7700 ft [2350 m], 6 Sep 1944, *A.J.Sharp 44688* (holotype: US-1939432!; isotype MO-1410403!). *Poa venosa* Swallen Contr. U.S. Natl. Herb. 29(9): 399. 1950. Type: Guatemala, Huehuetenango, in alpine meadow, vicinity of Chémal, summit of Sierra de los Cuchumatanes, 3700−3750 m, 8 Aug 1942, *J.A.Steyermark 50310* (holotype: US-1935067!; isotypes: F-1201922, ISC-v-0000603 (image and fragment ex US)!, MO!).

### Description.

Hermaphroditic or simple gynomonoecious. **Perennials**; tufted, sometimes sub-rhizomatous, tufts loose, narrow to medium girth, medium height, green; tillers extravaginal (basally cataphyllous), with lateral tending cataphyllous shoots. **Culms** 35–75 cm tall, weakly erect, sometimes decumbent or geniculate at base, leafy, terete or slightly compressed, smooth or scabrous; nodes terete, 2–4, 1–3 exerted. **Leaf** sheaths compressed, keel not winged, smooth or lightly to moderately scabrous (to densely scabrous); butt sheaths papery, smooth, glabrous; flag leaf sheaths 5.8–15.2 cm long, margins fused 40–54% their length; collar margins smooth or lightly asperous, glabrous, or ciliate; ligules 1.25–3.0 mm long, abaxially scabrous or sometimes smooth; apices obtuse or sometimes acute, sterile shoot and lower culm ligules ca. 0.5–1 mm long, adaxially usually densely scabrous, apically usually densely scabrous margined; blades 2–17 cm long, 2–5 mm wide, flat or folded, margins sometimes becoming involute, thin to moderately thin, soft, surfaces and margins nearly smooth to moderately scabrous abaxially the keel prominent or not, adaxially prow-tipped; usually the middle culm leaves the longest (10–17 cm), flag leaf blades 34–58% their sheath in length, flag leaf blade 2.2–8.0 cm long. **Panicles** 7.5–20 cm long, nodding, open, sparse to moderately congested, with 25–85 spikelets, peduncles and axis sparsely to densely scabrous, proximal internode 2–4.5(–6.3) cm long; rachis with 2(–4) branches per node; primary branches spreading, flexuous, terete or weakly angled, moderately scabrous, to densely scabrous on pedicels, with coarse hooks; lateral pedicels usually 1/4–1/2 the spikelet in length, moderately to densely scabrous, prickles fairly coarse; longest branches 3–7(–11.5) cm, with 4–16(–22) spikelets, in distal 1/2, moderately crowded. **Spikelets** 4.2–6.5 mm long, lanceolate, laterally compressed, not bulbiferous, pale to grayish green; florets (2–)3–4, all hermaphroditic, or the distal ones pistillate; rachilla internodes terete, mostly 0.8 mm long, less than 1 mm long, smooth, glabrous; glumes narrowly lanceolate to lanceolate, unequal to subequal, usually green, distinctly keeled, keels smooth or sparsely to moderately scabrous distally, lateral veins smooth or lightly scabrous, surfaces smooth, margins narrowly scarious-hyaline, edges smooth, apex sharply acute; lower glumes 2.1–2.8 mm long, 1(–3)-veined, sometimes sickle shaped; upper glumes 2.7–3.5 mm long, distinctly shorter than lowest lemma by 0.4–0.9 mm long, 3-veined; calluses dorsally webbed, web scant or well developed, hairs 0.8–2.1 mm long, woolly; lemmas (3–)3.3–4.3 mm long, 5-veined (lowest sometimes 7-veined), lanceolate, 5–7-veined, 4.2–5.9 × longer than wide, body firmly chartaceous, grey green, with or without a anthocyanic flush just below the apex, and down the upper margin distinctly keeled, keels sparsely to moderately scabrous distally, keels for 1/3 to 3/4 and marginal veins 1/4–2/3, short to long villous, intermediate veins glabrous or sparsely sericate, between veins smooth, minutely bumpy or lightly scabrous, glabrous or sparely to moderately densely sericate, intermediate veins moderately prominent to prominent, margins and apex narrowly scarious, smooth or with few fine hooks, apices obtuse to acute, abruptly curved inward; paleas scabrous, keels coarsely scabrous, between keels minutely bumpy. **Flowers** weakly chasmogamous; lodicules 0.5–0.6 mm long, broadly lanceolate, with a slender lateral lobe above the middle; anthers 0.7–1.05(–1.25) mm long, or vestigial 0.1–0.2 mm long in distal flower(s). **Caryopses** 1.8–2 mm long, elliptical-fusiform in side-view, laterally compressed, light olivaceous brown, sulcus distinct, hilum 0.25 mm long, oval to elliptic, grain free or adherent to the palea. 2*n* = unknown.

### Distribution.

The species is found in Guatemala (Huehuetenango) and Mexico (Distrito Federal, Hidalgo, Mexico, Oaxaca, San Luis Potosí, Veracruz).

### Ecology.

*Poa ruprechtii* occurs in upland mesic forests and openings to subalpine habitats; primarily distributed on volcanoes of central Mexico between 2200–3050 m; southward to alpine meadows in northern Guatemala between 3700–3750 m. Flowering May through September.

### Specimen examined.

Mexico. **Distrito Federal:** Puerto de las Cruces, delegacíon de Cuajimalpa, 3100 m, 20 Jul 1980, J.Rzedowski 36739 (TAES). prope Santa Fe, E.Bourgeau 670 (MPU, LE, US-3159864 fragm. ex MPU, US-3159863 fragm. ex LE). 10 miles SW of Mexico City, 9000 ft [2740 m], 10 Aug 1947, F.A.Barkely 255, B.L.Westlund & J.B.Paxon (TAES). **Hidalgo:** Municipio de Mineral del Monte, La Minita, ca. 4 km al s. de la cabecera municipal, 2780 m, 13 Jul 1994, J.Praxedes-Peréz 120 (MEXU-1072096). **Mexico:** Municipio de Naucalpan, alrededores de Villa Alpina, 3100 m, 14 Jun 1981, J.Rzedowski 37312 (TAES). Municipio de Villa Nicolás Romero, 1 km al NW de Cahuacán, 2600 m, 21 Jul 1968, J.Rzedowski 25984 (MEXU-391678). Al sur del Nevado de Toluca, Cieneguillas de Cabcarr, Sultepec-La Puerta, 1 Aug 1981, R.Guzmán 4020 (MEXU-1072091). Municipio Huizquiluca, Rancho El Hielo, km 22 carretera Naucalpan-Toluca, 3050 m, 21 May 2001, A.Miranda et al. 577 (MEXU-1072128). **Oaxaca:** ca. 16 mi NE of Guelatao on highway 175 to Tuxtepec, 2800 m, 16 Aug 1975, G.Davidse 9771 (MO-2935776). **San Luis Potosí:** near Puerta Huerta in the Sierra de Alvarez, 2200−2400 m, 4 Sep 1954, E.R.Sohns 1039 (TAES, US-2154373). **Veracruz:** See type of *Poa sharpii* above.

### Discussion.

In 2005 the first author viewed the type collection of *Poa ruprechtii* at W and (regrettably) annotated it as “*Poa orizabensis* Hitchc.?" At that time there was no notation on this specimens indicating it was the type of *Poa ruprechtii*. Subsequent searches for the type at W by Bruno Wallnöfer, and other curators where *C. Heller* material from Mexico might exist (IB, K, LE, WU), did not relocate this collection until 2011. Thanks to the attentive eyes of Lia Pignotti (W), the type was rediscovered at W in the *Poa orizabensis* folder. The type was originally determined as *Poa annua* L. That epithet and author were then crossed out and replaced by *Poa flexuosa* Muhl. (Fig. 16; see discussion of *Poa flexuosa* under *Poa palmeri*). Heller collected on the north slopes of Volcán Toluca between August 10–14, 1846, near Hacienda Cocustepec above the “small hamlets San Buenaventura and Cacalomacan" (Heller and Rugeley 2007; an English translation of Heller’s travel’s in Mexico, originally published in German). The type locality for *Poa sharpii*, “El Puerto, 7700 ft" in Veracruz, is somewhere above the city of Orizaba on the southeast side of Volcán Orizaba. The species should also be searched for on or about the slopes of Volcán Orizaba in Puebla.

Specimens previously identified as “*Poa ruprechtii*" from Coahuila and Nuevo León, by Hitchcock (1913) and others, belong to *Poa palmeri* (see discussion under that species). Espejo Serna et al. (2000) and Dávila Aranda et al. (2006) accepted *Poa ruprechtii* s.l. and *Poa sharpii*. Beetle (1987) attempted to resolve the disposition of the northern plants by placing them in *Poa nervosa* (Hook.) Vasey. References to the name and material of *Poa ruprechtii* s.l. were simply left out of the volume of Beetle’s treatises on the grasses of Mexico that included *Poa* (Manrique-Skendzic 1999). The description of this species given here is based on the material from Mexico cited above. The name *Poa venosa* Swallen has been applied to this taxon in Guatemala, the type of which is a fairly robust specimen of the species, with lemmas quite hairy between the veins. However, other specimens from Guatemala to which that name has been applied are quite variable and approach *Poa orizabensis*. *Poa ruprechtii* differs from *Poa orizabensis* by having lemmas that are distinctly short villous on the keel and lateral nerves and sometimes puberulent between the nerves, versus lemmas that are glabrous or very sparsely puberulent on the base of keel and sometimes the marginal vein, and glabrous elsewhere. Also the glumes in *Poa ruprechtii* are narrower in proportion to their length, and the spikelets are less compact than in *Poa orizabensis*. However, a few specimens display intermediate combinations of characteristics in Mexico (see notes on *Poa orizabensis* specimens cited).

*Poa ruprechtii* approaches *Poa talamancae* R.W. Pohl (Type from Costa Rica, Prov. de Cartago: 83 km from San José on the Pan American Highway, Asuncion (summit of Cerro de la Muerte), open windswept subparamo, 3335 m, 22 Jul 1966, *S.Mori 214 & R.Anderson* (holotype: ISC-324356!; isotypes: LE!, WIS!). However, more study is needed here also.

## 18. Poa scaberula

Hook. f., Fl. Antarct. 2: 378 1846.

http://species-id.net/wiki/Poa_scaberula

Figs 13 I–L 19

### Type:

**Figure 19.**
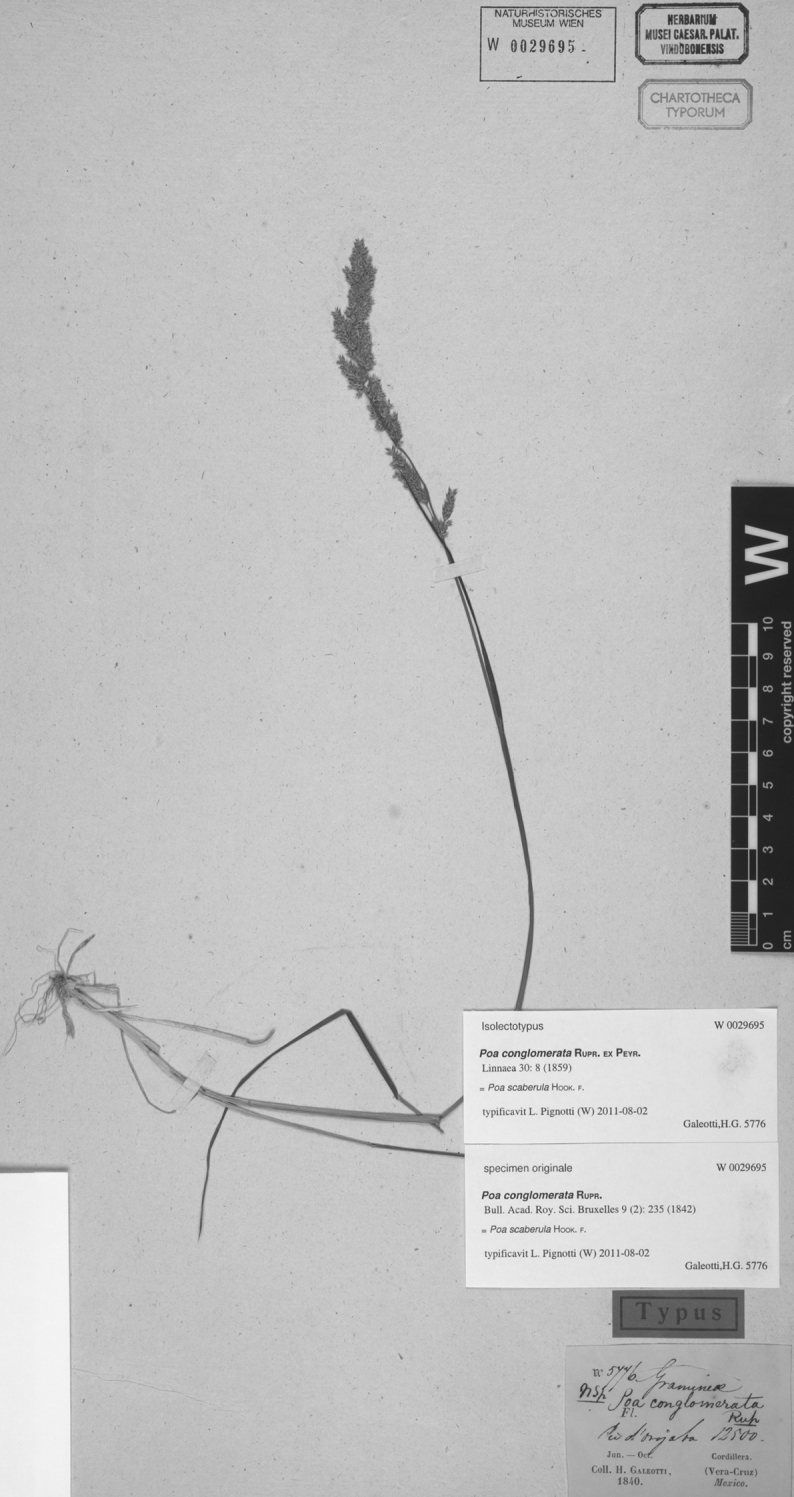
*Poa scaberula* Hook.f. Photo of lectotype collection *Poa conglomerata* Rupr. ex Peyr. (*Galeotti 5776*).

Chile, Straight of Magalhaens, Port Famine, *King s.n*. (holotype: K!; isotypes: BAA! fragm., GH). *Poa conglomerata* Rupr. ex Peyr., Linnaea 30(1): 8. 1859. *Poa conglomerata* Rupr., nom. nud., in Galeotti, Bull. Acad. Roy. Sci. Bruxelles 9(2): 235. 1842. Type: Mexico, Veracruz, Cordillera, Pic d’ Orizaba, 12500 ft [3810 m], Jun-Oct 1840, *H.G. Galeotti 5776* (lectotype: W-29695! designated here, partial lectotype designated by Hitchcock 1913: 374; isolectotypes: LE!, P!, US-89680! fragm. ex P). Fig. 19. *Dasypoa tenuis* Pilg Bot. Jahrb. Syst. 25(5): 716–717. 1898. Type: Peru, Lago Titicaca, Tiquina, 3800 m, 13 Jan 1877, *M.A.Stübel 60f* (holotype: B!; isotypes: BAA! fragm. ex B, US-865610! fragm. ex B). *Poa anfamensis* Negritto & Anton, Darwiniana 35(1–4): 159–161, f. 1. 1998. *Poa micranthera* Hack., Anales Mus. Nac. Buenos Aires, 21: 154. 1911. nom. nud. Type: Argentina, Tucuman, Tafi, Cuesta de Anfama, 2000m, 24 Jan 1907, *Lillo 5468* herb. Stuckert 19827 (holotype: W!; isotypes: LIL!, US-88758! fragm. ex W); *Poa dactyliformis* Steud., Syn. Pl. Glumac.1: 426. 1854. Type: Chile, Punta de Arenas, Jan 1853, *Lechler W*. (R.F. Hohenacker exsiccate) *1151* (holotype: P; isotypes: LE!; SGO!, US-89676! fragm. ex SGO & photo, W!). *Poa maullinica* Phil., Anales Univ. Chile 94: 164. 1896. Type. Chile, Provincia de Llanquihue, Maullin, *Carlos Juliet* (holotype: SGO-PHIL-416!; isotypes: SGO-45753!, SGO-37322!, US-A2947091! fragm. ex SGO-PHIL-416 & photo, US-1763025!).

### Description.

Hermaphroditic. **Perennials**, short-lived; tufted, tufts dense or loose, usually narrow girth, small to moderate height (2–25 cm tall), usually delicate, green; tillers intravaginal (each subtended by a single elongated, 2-keeled, longitudinally split prophyll), without cataphyllous shoots. **Culms** 8–80 cm tall, slender, erect or ascending, usually slender, leafy, terete or lower internodes slightly compressed, smooth; nodes terete, 2–4, 0–3 nodes exposed or exerted. **Leaf** sheaths laterally compressed, distinctly keeled, lightly to moderately scabrous, glabrous; flag leaf sheaths (2–)4–15 cm long, margins fused 25–32% the length; throats and collars smooth or slightly scabrous, glabrous; ligules 1–4 mm long, sometimes decurrent, sub-hyaline, milky-white, abaxially scabrous, apex obtuse to acute, sterile shoot ligules shorter than those of the upper culm leaves; blades mostly 5–10 cm long, 1–3.5(–4) mm wide, flat, thin, lax, soon withering, distinctly keeled, surfaces and margins sparsely to densely scabrous, apex slenderly prow-tipped; flag leaf blades little reduced; sterile shoot blades like those of the culm. **Panicles** 3–15 cm long, erect, contracted, sub-cylindrical, usually somewhat interrupted, lobed, congested, with 30 to well over 100 spikelets, peduncle and axis moderately to densely scabrous; rachis with 2–3 branches per node; primary branches erect or slightly spreading, fairly strict, angled, densely scabrous on and between angles; lateral pedicels mostly less than 1/5 the spikelet, < 0.5 mm long, densely scabrous, prickles moderately coarse; longest branches 1–7 cm, with 4–60 spikelets, with spikelets congested from the base up to from the lower 1/3 to the apex. **Spikelets** 3–4 mm long, lanceolate when closed, lanceolate, laterally strongly compressed, not bulbiferous, not shiny, green or sometimes anthocyanic; florets (2–)3–4(–5), hermaphroditic; rachilla internodes terete, mostly 0.3–0.4 mm long, smooth, minutely bumpy, or slightly scabrous, glabrous; glumes narrowly lanceolate to lanceolate with a narrow scarious margins, unequal to subequal, shorter than adjacent lemmas, keels scabrous, lateral veins and surfaces smooth or distinctly scabrous, margins scabrous, apex acuminate, sharply pointed, straight or somewhat sickle shaped; lower glumes 1.4–2.8 mm long, 1-veined; upper glumes 1.8–3 mm long, (1–)3-veined; calluses dorsally webbed, commonly with additional webs below the marginal veins, hairs 1–3 mm long, woolly; lemmas 2.5–3 mm long, 5-veined, broadly lanceolate, strongly keeled, keel nearly smooth or scabrous for the upper 1/2, keel to 1/2 and marginal veins to 1/2 sericious, between veins glabrous, distally smooth or scabrous, intermediate veins faint to distinct, sometimes slightly scabrous, margins narrowly scarious distally, scabrous on the edge, apex acute, pointed; paleas distinctly shorter than the lemma, keels scabrous, between keels smooth or lightly scabrous. **Flowers** mainly cleistogamous or weakly chasmogamous; lodicules 0.5 mm long, lanceolate with a distinct narrow lateral lobe near the middle; anthers 0.4–0.7 mm long. **Caryopses** 1.1–1.3 mm long, elliptical in side-view, slightly laterally compressed, honey-colored, sulcus shallow, hilum 0.1 mm long, punctiform grain adherent to the palea, styles short, stigmas sparse. 2n = unknown.

### Distribution.

The species is found in North, Central, and South America; Mesoamerica: Guatemala; Mexico: Chiapas, Distrito Federal, Mexico, Morelos, Puebla, Tlaxcala, Veracruz; South America: Argentina, Bolivia, Chile, Colombia, Ecuador, Peru.

### Ecology.

In Mexico the species is found in mesic, cool temperate forest openings to low alpine habitats, particularly on volcanic substrates, and frequently associated with some disturbance; ranging from 2400−4450 m. Flowering July through October.

### Specimens examined.

Mexico. **Chiapas:** 1 km al E de Rizo de Oro, 850 m, 17 Oct 1985, P.Dávila 148, E.Martinez, R.Riba & J.L.Villasenor (MEXU). **Distrito Federal:** Giles-R. 57 (MEXU). J.Rzedowski 1540 (MEXU). J.Rzedowski 1979 (MEXU, MEXU). Ellis 1117, Dunn & LeDoux (MO). L.Pacheco 52 & P.Dávila (MEXU). A.Miranda 880 (MEXU). G.Villegas & R.Medez (MEXU). A.Miranda 61 & P.Guerrero (MEXU, MO). A.Miranda 74 & P.Guerrero (MEXU). Municipio De Chalco, faldas del Cerro Telapón, 3150 m, 26 Aug 1964, E.Arrington s.n. (TEX). Municipio Talpan, Volcán el Pelado, 3470 m, 31 Aug 1986, Campos E35-10 et al. (MEXU). 25 mi SE of Mexico City, 9000 ft [2740 m], 2 Aug 1947, F.A.Barkley 2427, C.M.Rowell Jr. & G.L.Webster (TEX). **Mexico:** Toluca, 8800 ft [2680 m], C.Heller 308 (W syntype *Poa conglomerata*); ditto, C.Heller 309 (W syntype *Poa conglomerata*). 55 km SE of Mexico City, 10500 ft [3200 m], 11 Jul 1942, J.N.Weaver 758 (TAES, TEX, US); ditto, 14 Jul 1942 (TEX); ditto, 14 Jul 1942, J.N.Weaver 787 (TAES). Municipio de Acoyoacac, 5 km al S del la Marquesa carratera La Marquesa–Tiangistengo (19°15'30"N, 99°22'31"W), 24 Oct 1997, L.Aragón-M. 676 (MEXU). Municipio Jilotzingo, 13 km de Santa Ana Jilotzingo por la carretera hacia la Presa Iturbide, 3230 m, 28 Aug 1984, M. Gonzáles 111, J. García & I.Hernández (TAES). Volcan de Toluca, 3550 m, 18 Oct 1953, E.R.Sohns 1005 & E.Matuda (TAES). camino de Toluca al Nevado de Toluca, 2900 m, 4 Aug 1962, J.Rzedowski 15821 (TAES). 22.5 km SW of Toluca on road to Nevado de Toluca, 3500 m, 07 Oct 1991, P.M.Peterson 11087 & C.R.Annable (US). W slopes of Nevado de Toluca, 35 km SW of Toluca on highway 130, 3000 m, 29 Aug 1965, S.Mori 1498 (p.p.), K.Roe & E.Roe (TAES). 1.7 mi E of Highway 10 along road to Nevado de Toluca, 19°8’4.9"N, 99°47’45.5"W, 3688 m, 9 Oct 2007, P.M.Peterson 21351, J.M.Saarela & M.J.Flores-Villegas (US). Cerros Tenaya, Soneyo, 2600 m, 27 Jul 1952, E.Matuda 26248 (MEXU). N slopes of Nevado de Toluca, campo de Rafael Alvarez y Fidel Garcia, weedy fallow portion of field, 3340 m., 20 Aug 1962, D.Ugent 1242, V.Ugent & R.Flores-C. (WIS). 27 km. SW of Toluca on road to Temaxcaltepic, ca. 2800m., 9 Jul 1964, sin collector 229 (WIS). S slope of Nevado de Toluca, along road, ca. 3200ft [975 m] 16 Jul 1954, G.B.VanSchaack 3395 (WIS). W slope Nevado Toluca, 2800 m, 16 Jul 1954, G.B.VanSchaack 3390 (WIS); ditto, 3392 (MEXU, WIS). La Puerta, 2 km por la Carretera al Nevado de Toluca sur, 1 Aug 1981, R.Guzman 3973 (MEXU). entre Raices y La Peñas, al S de Toluca, 3304 m, 1 Aug 1981, R.Guzman 3988 (MEXU). km 15 de la desviacion a Sultepec (carratera Toluca-Temasclatepec), 3420 m, 28 Jul 1983, E.Manrique 273, Guerrero & Miranda (MEXU). A 7 km de Buenavista, rumbo a Temascaltepec, 3250, E.Manrique 286, Jaramillo & Guerrero (MEXU). Ixtaccihuatl, Oct. 1905, C.A Purpus 1638 (MO, US). road to Paso del Cortez, 3370 m, S.D.Koch 76252 (MEXU, MO, TAES, US); ditto, 3300 m, S.D.Koch 76273 (MO, TAES). 6.7 mi E of Amecameca toward Paso de Cortez, 19°4’14.2"N, 98°41’32.1"W, 2488 m, 11 Oct 2007, P.M.Peterson 21381, J.M.Saarela & M.J.Flores-Villegas (US). P.N. Izta-Popo, 8 Jul 1980, A.A.Beetle M-5211 (MO). Mount Popocatepetl, 5–6 Aug 1910, A.S.Hitchcock 1204 (LL, TAES). vertiente northwest del Popocatepetl, 3200 m, 15 Jul 1965, J.Rzedowski 20192 (TAES). Popocatepetl, Refugio de Tlamancas, 3850 m, 20 Nov 1983, A.Ramirez-E. Popo-4 (MEXU). 12.5 miles from turnoff to the summit of Popocatepetl, 3460 m, 18 Oct 1976, J.Brunken 420 & C.Perino (MO TAES). 11 km by road E of Amecameca highway 115 jct., on road to Paso del Cortez and Popo, in big curve, 19.0877°N, 98.679°W, 2990 m, 2 Oct 1987, R.J.Soreng 3301 & N.Soreng (US). 17 km by road E of Amecameca highway 115 jct., west of Paso del Cortez, 19.097°N, 98.683°W, 3325 m, 2 Oct 1987, R.J.Soreng 3306 & N.Soreng (US). 1 km N of Paso del Cortez toward Ixtaccihuatl, 19.098°N, W98.6564°W, 3690 m, 2 Oct 1987, R.J.Soreng 3310 & N.Soreng (US). 1 km N of La Joya Trail Head, southwest flank of Ixtaccihuatl, 19.1507°N, 98.6525°W, 3965 m, 2 Oct 1987, R.J.Soreng 3313 & N.Soreng (US; Soreng 1990, cpDNA voucher “3315" error for 3313). 4 km N of la Estacion Retransmisora, bertienente southeast del Ixtaccihuatl, 3800m., 15 Jul 1965, J.Rzedowski 20177 (TAES, WIS). Sierra de las Cruces, 1 Oct 1892, C.G.Pringle 4307 (MO, TAES, US, US). **Morelos:** km 45 carretera México-Cuernavaca, 2.5 km adelante de Parras, 26 Jul 1980, G.Andrade s.n. & C.H.Ramos (MEXU-1071983). **Puebla:** Parque Nacional Izta-Popo, 0.5 mi east of Paso de Cortez at entrance to park, 19°5'17.8"N, 98°38'25.2"W, 3671 m, 11 Oct 2007, P.M.Peterson 21394, J.M.Saarela & M.J.Flores-Villegas (US). Paso del Cortez, ca. 1 km ENE de la retransmisora de television, 3860 m, 9 Nov 1976, S.D.Koch 76257 (TAES). base of Mt. Orizaba, 9000 ft [2740 m], 26 Jul 1901, C.G.Pringle 9594 (ISC, MEXU, MO, TAES, US). NW slopes of Orizaba, 23 rd km above Tlatlachichua ca. 6 km north-northwest of Pico de Orizaba, near head of Rio Quatzalapa, ca. 2 km SW of Cerro Mihas, 19.086°N, 97.288°W, 3660 m, 4 Oct 1987, R.J.Soreng 3326a & N. Soreng (US, DNA voucher, unpublished). Sierra Negra (southwest of Pico Orizaba), east side of mountain, 4080−4240 m, 10 Sep 1958, J.H.Beaman 2518 (MEXU, TEX, WIS). **Tlaxcala:** Tlaxcala, 3000 m, 13 Jul 1954, G.B.VanSchaack 3383 (MEXU, WIS). 6 km de Terrenate rumbo a Cerro Alto, 3100 m, 19 Jul 1985, E.Manrique et al. 1111 (MEXU). Municipio Chiautempan, 200 m de la est. de microondos rumbo al refugio de La Malinche, 3000 m, 5 Sep 1981, S.H.Contreras 593 (MEXU); ditto, S.H.Contreras 599 (MEXU). Municipio Huamantla, carretara a Teacalco, 10 km sobre la desva. al albergue de la Malinche, 3360 m, 2 Aug 1983, Guerrero 614, Romero, Rodrigues & DelaMora (MEXU). **Veracruz:** Mt. Orizaba, 10000 ft [3050 m] 1 Aug 1901, H.E.Seaton190 (US). 25−26 Jul 1901, J.N.Rose 5732 & R.Hay (MEXU). Sep 1907, C.A.Purpus 2887 (US). Sep 1907, C.A.Purpus 2888 (MO, NY, NY, US). Mar 1908, C.A.Purpus 3014 p.p. (MO). Municipio La Perla, n. side of Pico Orizaba above the Piedra Grande mountaineering shelter, 19°03'N, 97°16'W, 4450 m, 21 Sep 1986, M.Nee 33170, R.Roblez-G., R.Acevedo-R. & J.L.Martinez-P. (MO). Pico de Orizaba, south side of mountain, north of Cueva del Muerto, 21 Sep 1957, J.H.Beaman 1776 (TEX). Municipio Perote, Parque Nat., Cofre de Perote, 2 km del camino de El Conejo al Cofre, 19°33'N, 97°09'W, 3350 m, 5 Jul 1983, H.Narave-F. et al. 750 (MEXU). 19°31'55"N, 97°09'19"W, 3300 m, 28 Mar 1995, P.J.Parroquín 88 (MEXU); ditto, P.J.Parroquín 88-A (MEXU). Cofre de Perote, 2 km del camino de El Conejo al Cofre, 19°30'N, 97°18'W, 4200 m, 13 Jan 1979, O.Castillo-O. 445 & R.Ortega-O. (MEXU); ditto, 19°29'31"N, 97°08'51"W, 4200 m, 3 Jun 1994, C.R.Galindo 47 (MEXU); ditto, summit, 4140 m, 20 Sep 1997, S.J.Darbyshire 4798 & M.González-Ledesma (US).

### Discussion.

In Mexico and Guatemala this taxon was known as *Poa conglomerata* until Soreng et al. (2003) placed it in synonymy of *Poa scaberula* (Dávila Aranda et al. 2006, followed this). *Poa scaberula* was previously known only from South American where it is scattered along the Andes from Colombia to Tierra del Fuego in cool temperate forests and mesic puna. There is no character by which to separate the Central American material from the South American plants, both of which are highly variable in stature. Plants with glabrous lemmas, but still with a web on the callus occur occasionally throughout the range of the species but are more frequently encountered in Mexico than elsewhere. Hitchcock’s incomplete lectotypification of *Poa conglomerata* of *Galeotti 5776* was mistakenly treated by Manrique de Skendzic (1999) and Soreng et al. (2003) to have been on the US fragment (which is the tip of a branch with a dense cluster of spikelets obtained by A. Chase in 1927). That is not adequate since the original Galeotti collection from which the US fragment was taken presumably still exists at P, and other sheets are at LE, possibly BR, and are known from W (where Peyritsch worked). We lectotypify on the W collection since it is the only specimen of *Galeotti 5776* that we can be reasonably certain Peyritsch saw when he described the species.

## 19. Poa secunda secunda

J. Presl. Reliq. Haenk. 1(4–5): 271 1830.

http://species-id.net/wiki/Poa_secunda_secunda

Fig. 20 A–G

### Type:

Chile, Cordilleris Chilensibus, 1790, *T.Haenke* (holotype: PR!; isotypes: B, BAA-2691! fragm. ex B, GH! fragm., LE!, LE-TRIN-2625.01a!, MO-209304!, US-88729! fragm. ex PR).*Poa orcuttiana* Vasey, W. Amer. Sci. 3: 165−166. 1887. Type: USA, California, near San Diego, 26 May 1884, *C.R.Orcutt 1070* (holotype: US-556833!).*Atropis scabrella* Thurb., Bot. California 2: 310–311. 1880. ). *Poa scabrella* (Thurb.) Benth. ex Vasey, Grass. U.S. 42. 1883. *Panicularia scabrella* (Thurb.) Kuntze, Revis. Gen. Pl. 2: 783. 1891. *Puccinellia scabrella* (Thurb.) Ponert, Feddes Repert. 84(9–10): 740. 1974. Type: USA, California, Oakland, *H.N.Bolander* (holotype: NY! ex herb. Nash ex herb. Thurber; isotype: US-556836! fragm. ex herb. Nash ex herb. Thurber & photo).

### Description.

Hermaphroditic. **Perennials**; tufted, tufts dense, narrow to medium girth, tiny to medium height (2 to over 20 cm tall), usually narrow based, green or bluish-grey-green; tillers intravaginal (each subtended by a single elongated, 2-keeled, longitudinally split prophyll), without cataphyllous shoots, sterile shoots more numerous than flowering shoots. **Culms** (10–)15–80(–100) cm tall, erect or bases slightly decumbent, slender to stout, leaves mostly basal, terete or weakly compressed; nodes terete, 0–2 exerted. **Leaf** sheaths terete, scabrous, glabrous; butt sheaths papery, smooth, glabrous; flag leaf sheaths (7–)10–20(–25) cm long, margins fused 10–25% the length, (0.95–)1.5–7(–15) × long as its blade; collars smooth or scabrous, glabrous; ligules of culm leaves 2–6(–10) mm long, of sterile tillers mostly 2–6 mm long, abaxially smooth or scabrous, apices truncate to acuminate, sterile shoot ligules similar to those of the culm leaves, blades of cauline leaves 0.4–3 mm wide, flat, folded, thin, soft, and soon withering, lax. smooth, or scabrous mainly over veins, glabrous, narrowly prow-tipped; blades gradually reduced distally or the middle blades, flag leaf blades 0.8–10(–17) cm long; sterile shoot blades similar in form to cauline blades. **Panicles** 2–15(–20) cm long, erect, contracted at maturity, narrowly lanceoloid to ovoid, usually congested or moderately congested (except in flowering), with 10 to over 100 spikelets; rachis usually with 1–3 branches per node; primary branches erect or ascending, infrequently widely spreading at maturity. terete to weakly angled, on and between angles usually sparsely to densely scabrous; lateral pedicels 1/6–1/3 the spikelet length, moderately to somewhat densely scabrous, prickles fairly coarse; longest branches (0.5–)1–8(–10) cm, with (1–)2–20(–60+) spikelets in distal 2/3–1/2. **Spikelets**, (4–)5–8 mm long, (3.8–)4–5 × long as wide, usually narrowly lanceolate, sub- terete to weakly laterally compressed, not bulbiferous, drab, green or strongly anthocyanic, sometimes glaucous; florets (2–)3–5(–10), hermaphroditic; rachilla internodes terete or slightly dorsaventrally compressed, usually 1–2 mm long, smooth or muriculate to scabrous or hirtellous; glumes lanceolate to broadly lanceolate, slightly unequal, keels indistinct, keel and upper sides scabrous, apex acute to acuminate, lower glumes (2.5–)3–3.5 mm long, 3-veined; upper glumes 3.5–4 mm long, 3-veined; calluses glabrous, or with a crown of hairs around the base of the lemma, hairs 0.1–0.5(–2) mm long, crisp or slightly sinuous; lemmas 3.5–6 mm long, lanceolate to narrowly lanceolate or slightly oblanceolate, usually weakly keeled, lemma keels and marginal veins short villous to crisply puberulent or softly puberulent over proximal 2/3, between veins usually at least sparsely crisply puberulent or softly puberulent, hairs usually less than 0.5 mm long; intermediate veins obscure, margins strongly inrolled below, broadly scarious above, apices obtuse to broadly acute, blunt or sometimes pointed; paleas scabrous along the length or medially often short villous to softly puberulent over the keels, intercostal region often softly puberulent. **Flowers** chasmogamous; lodicules 0.55–0.8 mm long, broadly lanceolate, with a well developed lateral lobe nearly equaling the main lobe; anthers 1.5–3 mm long. **Caryopses** 2.2 mm long, fusiform in side-view, round on back, olivaceous, weakly sulcate, hilum 0.25 mm long, oval, grain free from the palea. 2*n* = 42, 44, 56, 63, ca. 68, 70, ca. 72, ca. 74, ca. 78, ca. 80, 82, ca. 83, 84, 85, 86, ca. 87, ca. 88, ca. 90, ca. 91, 93, ca. 94, ca. 98, ca. 99.

### Distribution.

This species is widespread in North America: Canada (all Provinces except New Brunswick, Nova Scotia, Nunavut, PEI); USA (Alaska, and all western states); Mexico (Baja California); South America (Argentina and Chile).

### Ecology.

The species occurs in coastal chaparral communities to coniferous forests at over 1000 m elevation in Baja California. It is high polyploid (Soreng 1991a) and predominantly apomictic over much of its geographic range (Kellogg 1987). Flowering March to May in B.C.

### Specimens examined.

Mexico. **Baja California:** Municipio Ensenada, Arroyo de Agua Caliente, al E de Ojos Negros, 1 May 1981, R.Guzmán-M. 1341 (MEXU). Camino entre rancho Mike’s y San Jose Mellin, R.Guzmán-M. 1414 (MEXU). Tia Juana Valley, 6 Apr 1882, C.G.Pringle 37 (LL, US, US). Todos Santos Bay, Apr 1882, F.E.Fish 28 (US); ditto, 30 (US); ditto, 33 (US). Canutillas Mts., 1884, C.R.Orcutt 1148 (US). Guadalupe Canyon, 10 Apr 1885, C.R.Orcutt 1269b (US). Carisito, 8 Apr 1885, C.R.Orcutt s.n. (US). near Vallecito, 5 Apr 1886, C.R.Orcutt 1440 (MO, US); South slope 8 miles from Rosario on road to El Marmol, 4 Mar 1930, I.L.Wiggins 4338 (LL, US). 11 mi E of Tecate on road to Mexicali, 3600 ft [1095 m], 23 Dec 1971, A.A.Beetle M-1403 (TAES); Humorosa, 4270 ft [ m], 15 Apr 1973, A.A.Beetle M-2710 (TAES). 5 mi E of Humorosa, 3000 ft [910 m], 15 Apr 1973, A.A.Beetle M-2699 (TAES). Just east of Rancho Cuevas, 5 mi S of Rosarito, 32°17'N, 117°01'W, 60 m, 30 Mar 1975, R.Moran 21704 (TAES). Sierra Blanca, east of main summit, 32°03.5'N, 116°30.5'W, 1175 m, 16 May 1976, R.Moran 23237 (TAES). Sierra Juárez, 12 May 1997, A.Carrillo-S. 96 (MEXU). Laguna Hansen, 1 May 1981, R.Guzmán-M. 1351 (MEXU). Sierra Juárez, 5 km W of La Rumorosa, 32°33'N, 116°06'W, 1325m, 15 May 1977, R.Moran 24100 (TAES). Rancho El Potrereo, 40 km al SW de el Observatorio de Sierra San Pedro Mártir, 1050 m, P.Tenorio-L. 13218 & C.Romero-deT. (MEXU). Sierra San Pedro Mártir, arroyo 3 km e of El Soccoro, 30°58.4'N, 115°37.8'W, 1450 m, 8 May 1978, R.Moran 25702 (TAES).

### Discussion.

Hitchcock (1913, 1935) accepted *Poa scabrella* with *Poa orcuttiana* as a synonym. Espejo Serna et al. (2000) accepted *Poa orcuttiana* and *Poa scabrella*; and Dávila Aranda et al. (2006) accepted *Poa scabrella* as synonym of *Poa secunda* subsp. *secunda* but *Poa orcuttiana* as a synonym of *Poa secunda* subsp. *juncifolia*. In our opinion *Poa orcuttiana* and *Poa scabrella* should be placed as synonyms of *Poa secunda* subsp. *secunda* (Soreng 1991a, 2007). There is some question regarding the geographic origin of the type of *Poa secunda*. It could be from California, and it seems unlikely that Haenke would have missed it there, whereas it is uncommon, at least today, in the southern Andes. There is no doubt in our minds that the North and South American plants are *Poa secunda* subsp. *secunda* (DNA sequences from South American specimens align with those from North America; Gillespie pers. comm.). If *Poa scabrella* formswere to be recognized, it would be best to treat them as a varietyof *Poa secunda* subsp. *secunda*, but this is not proposed here because it would only cause confusion selecting the correct name at the varietal rank among the many synonyms for this taxon. For this species Gould and Moran (1981) cite Isla Guadalupe as a location, possibly based on *R.Moran 13816* (TAES) but this specimen is *Koeleria pyramidata* Lam. [incl. *Koeleria cristata* s. auct., *Koeleria macrantha* (Ledeb.) Schult.].

**Figure 20.**
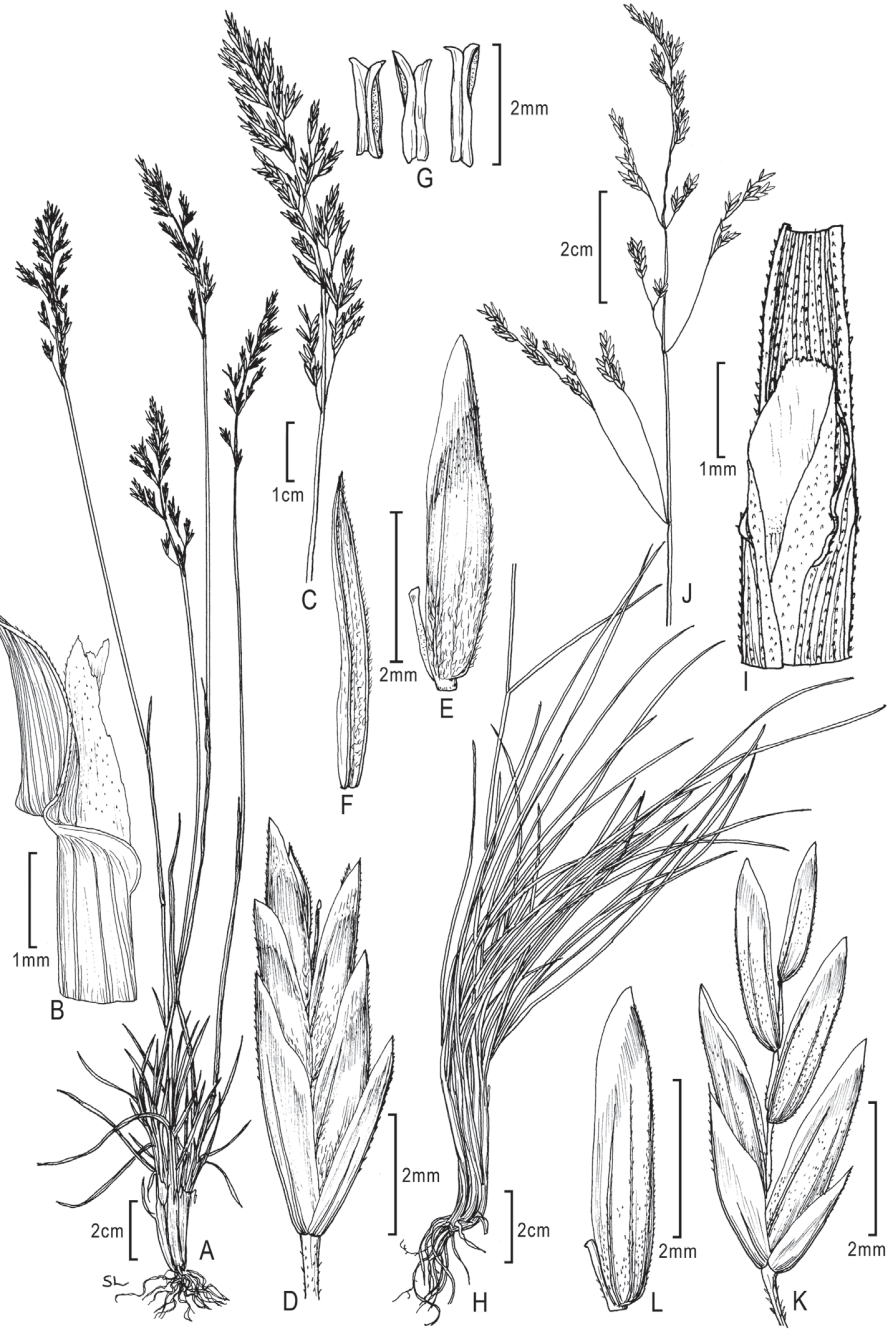
**A–G** *Poa secunda*J.Presl. subsp. *secunda* **A** habit **B** sheath, ligule, blade **C** inflorescence **D **spikelet **E** floret **F** palea **G** anthers **H–L** *Poa strictiramea* Hitchc. **H** basal tuft **I** sheath,ligule, blade, ventral view **J** inflorescence **K** spikelet **L** floret. Drawings **A–L** from Soreng (2007) **H, J, L** originally drawn from *Swallen 1110* as *Poa involuta* in Hitchcock (1935).

## 20. Poa seleri

Pilg., in T. Loesener, Verh. Bot. Vereins Prov. Brandenburg 51(Abhandl.): 17. 1909.

http://species-id.net/wiki/Poa_seleri

Figs 13 E–H 21

### Type:

Guatemala, Quezaltenango, und Sololá, Bergwold in Totonicapam und Los Encuentros, 25 Sep 1896, *E.Seler 2360* (lectotype: US-1389285! designated here; isotypes: BAA-2693! fragm. ex B, GH!).*Poa guatemalensis* Hitchc. Proc. Biol. Soc. Wash. 40: 81−82. 1927. Type: Guatemala, [Sacatepequez], Volcano Agua, in shade, medium altitude, 2000–3500 m, 5 Dec 1911, *A.S.Hitchcock 9115* (holotype: US-924985!).*Poa tacanae* Swallen, Contr. U.S. Natl. Herb. 29(9): 399 1950. Type: Guatemala, San Marcos, Volcán Tacaná, on wooded lower slopes between Sibinal and summit of Volcán Tacaná, 2500−4400 m, 19 Feb 1940, *J.A.Steyermark 36083* (holotype: F-1059917; isotype: US-2236477! fragm. & photo ex F).

### Description.

Hermaphroditic. **Perennials**; tufted, sometimes sub-rhizomatous, tufts loose, narrow girth, low to moderate height, delicate, green; tillers extravaginal (basally cataphyllous), with or without lateral or downward tending, cataphyllous shoots. **Culms** (20–)18–65(–95) cm tall, erect or weakly ascending, sometimes weakly geniculate, sometimes decumbent at the base, slender, leafy, terete, smooth or more often scabrous (especially below the mid- and upper nodes); nodes 3–4, 1–3 exerted. **Leaf** sheaths compressed, (smooth) scabrous, glabrous; butt sheaths becoming papery to somewhat fibrous, bases of butt sheaths glabrous; flag leaf sheaths 4–15 cm long, margins fused 40–53% their length, 2.3–6.5 × longer than its blade; throats and collars smooth or scabrous, glabrous, collars often flared; ligules (0.5–)0.8–3 mm long, scarious-hyaline, abaxially smooth or scabrous, glabrous, apex obtuse to acute; blades 5–20 cm long, (1–)1.5–3 mm wide, flat, thin, often lax, abaxially with veins closely spaced and expressed, smooth or lightly to moderately scabrous, margins proximally smooth, distally scabrous, adaxially lightly to moderately scabrous, apex slender prow-tipped; flag leaf blades 2–7 cm long. **Panicles** (4–)6.5–15 cm long, nodding, open, slender (to 5 cm wide), axis somewhat flexuous, moderately congested to sparse, with 25–100 spikelets, peduncles scabrous, proximal internode 0.9–3.5 cm long; rachis with 1–2(–3) branches per node; primary branches spreading to reflexed, or pendulous, slender, flexuous, terete to slightly angled, proximally smooth or sparsely scabrous, distally moderately scabrous on the angles and sparsely off them; lateral pedicels less than 1/2 the spikelet length, sparsely to moderately scabrous, prickles coarse or moderately coarse; longest branches 1.5–4 cm, with 5–10 spikelets loosely arranged in the distal 2/3–1/2. **Spikelets** 3.2–5(–5.5) mm long, 3–3.5 × as long as wide, lanceolate, laterally compressed, not bulbiferous, florets 2–3(–4), hermaphroditic; rachilla internodes terete, often visible from the side, regularly (0.5–)0.7–0.9 mm long, slender, smooth, glabrous; glumes green to anthocyanic, strongly unequal, distinctly keeled, keels scabrous, margins narrowly scarious-hyaline, apex acute to acuminate; lower glumes 0.6–1.8(–2) mm long, 1-veined, subulate to slightly sickle shaped, 1/4−1/2 the length of the proximal lemma (rarely longer and narrowly lanceolate); upper glumes 1.5–2.5 mm long, mostly 2–3 × as wide as the lower one, 3-veined; calluses glabrous; lemmas 3.1–4.1 mm long, 5-veined, 5-veined, lanceolate, body chartaceous, green, sometimes apically with a slight to prominent anthocyanic band, exceeded by a slight bronzy band; keeled, glabrous, keel and marginal veins smooth or distally lightly scabrous, between veins distally smooth (faintly muriculate) or distally sparsely scaberulous, intermediate veins faint to distinct, margins and apex very narrowly scarious-hyaline, edges scaberulous, apex entire, acute; paleas distinctly shorter to equaling their lemma, glabrous, keels smooth or sparsely scabrous, between keels smooth. **Flowers** weakly chasmogamous; lodicules 0.4 mm long, lanceolate to broadly lanceolate, without a lobe; anthers 0.4–0.8 mm long. **Caryopses** 1.5–1.8 mm long, elliptical in side-view, slightly laterally compressed, pale greenish-brown, sulcus distinct, hilum 0.2 mm long, oval, grain adherent to the palea. 2*n* = unknown.

### Distribution.

The species is found in Guatemala and Mexico (Chiapas).

### Ecology.

In Mexico (Chiapas) the species occurs in cool temperate forests between 2700–3600 m. Flowering September to December.

### Conservation status.

The species is rare in Mexico and is known from only three collections, but is not rare in Guatemala (14 collections seen from departments of Huehuetenango, Quezaltenango, San Marcos, Sololá, Sacatepéquez, Totonicapán).

### Specimens examined.

Mexico. **Chiapas:** Municipio Motozintla de Mendoza, 2700 m, 24 Nov 1981, D.E.Breedlove 55829 [“1"; “2" = *Peyritchia* sp.; fide and subnumbered by RJS 2011] (MO-2997613). Municipio Unión Juárez, summit of Volcán Tacaná, ca. 15.131°N, 092.108°W, 3600 m, 10 Nov 1972, D.E.Breedlove 29381 (DS, MO, TAES). Unión Juárez, 3600 m, 10 Nov 1974, D.E.Breedlove 29364 (MO).

### Discussion.

The distribution for this species given by Espejo Serna et al. (2000) and Dávila Aranda et al. (2006) is in error, probably a result of confusion with other species. The salient differences purported to discriminate between *Poa tacanae* and *Poa seleri*, respectively, are: rhizomes present versus absent; blades 1−2 mm versus 2−3 mm wide; ligules 0.5−1 mm, versus 2−3 mm long; panicles 4−8 mm versus 7−19 cm long, with 1−2 versus 2−4 branches; spikelets 4.5−5.5 mm versus 3.2−4.5 mm long, lower glumes 1.8−2 mm versus 1.1−1.4 mm long, upper glumes 2−2.5 mm versus 1.8−2 mm, and lemmas 3.3−3.7 mm versus 2.2−2.7 mm long (Pohl and Davidse 1994). All of these character state ranges overlap between these two species. For example, *Beaman 3191* from the type locality of *Poa tacanae*, is clearly loosely tufted with lateral tending shoots has characteristics that overlap with *Poa seleri*. Moreover, in collections with intact bases, the habit is evidently loose, while all other features point to *Poa seleri*. Collections of both taxa from high elevations have spikelets that are elongated (rachillas exposed in side view), with short, narrow, 1-veined lower glumes, glabrous florets, and anthers 0.4−0.8 mm long. Swallen’s holotype of *Poa tacanae* is immature (US fragm. and photo), and the spikelets are narrower than they would be at maturity. These collections are considered to be high elevation forms of *Poa seleri*. The type and isotype of *Poa seleri* at B were destroyed (Hildemar Scholz, pers. comm. 2012), thus we take up the US isotype as lectotype.

**Figure 21.**
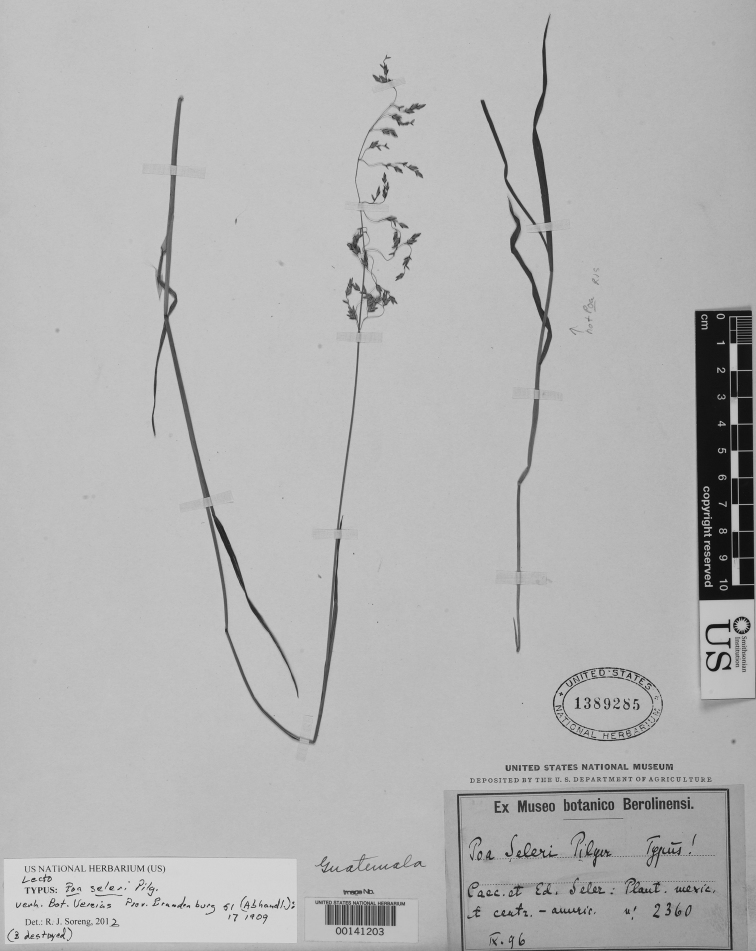
*Poa seleri* Pilg. Photo of lectotype collection (*Seler 2360*).

## 21. Poa strictiramea

Hitchc. Contr. U.S. Natl. Herb. 17(3): 375. 1913.

http://species-id.net/wiki/Poa_strictiramea

Figs 6 K–M 20 H–L

### Type:

Mexico, Chihuahua,cool ledges of La Bufa Mountain above Cusihuiriachic, 2 Sep 1887, *C.G.Pringle 1437* (holotype: US-820909!; isotypes: GH!, MA, MSC, NY-431381!, NY-431383!). *Poa involuta* Hitchc., Proc. Biol. Soc. Wash. 41: 159. 1928. Type: USA, Texas, Brewster Co., on the upper slopes of hills, Chisos Mountains, first ridge southwest of Juniper Canyon, 2200 m, 15−18 Jul 1921, *R.S.Ferris & C.D.Duncan 2811* (holotype: US-1125239!; isotypes: CAS!, US-1865159!). *Poa filiculmis* Swallen, Contr. U.S. Natl. Herb. 29(9): 400. 1950 (non. Roshev. 1949), *Poa coahuilensis* Beetle, Phytologia 52(1): 17 1982. Type: Mexico, Coahuila,15 km west of Concepcion del Oro, just within the Coahuila, 24°54'N, 101°45'W, valley floor sparsely covered by Yuccas and *Larrea*, 2300 m, 19 Jul 1941, *L.R.Stanford, K.L.Retherford & R.D.Northcraft 477* (holotype: US-1815803!; isotypes: ARIZ-10805!, GH!, MO-1221136!, NY-431379!, NY-431380!).

### Description.

Hermaphroditic (often functionally pistillate). **Perennials**; tufted, tufts dense, usually medium to medium large girth and height (11–30 cm tall), pale green or bluish-grey-green; tillers intravaginal (each subtended by a single elongated, 2-keeled, longitudinally split prophyll), and sometimes extravaginal (basally cataphyllous), all erect, sterile shoots more numerous than flowering shoots. **Culms** 30–90 cm tall, erect, or bases slightly decumbent, slender, not branching above the base, leafy terete, smooth or lightly scabrous above; nodes (2–)3, terete, upper (0–)1–2 exerted, uppermost at mid-height. **Leaves** mostly basal; leaf sheaths terete, moderately to densely scabrous, glabrous; butt sheaths papery, smooth, glabrous; flag leaf sheaths 8.5–22 cm long, margins fused 18–48(–75)% the length, fused by an invaginated hyaline margin for much of that length (0.5–)1–3.5 × longer than its blade; collars not flared, smooth or scabrous, glabrous or coarse ciliate; ligules 1.25–4(–6) mm long, adaxially moderately to densely scabrous, apices obtuse, entire or slightly dentate/lacerate, sterile shoot ligules like those of the culm leaves; blades of cauline leaves to 22 cm long, 1–2(–4) mm wide (expanded), involute or initially flat, moderately thick and moderately firm to firm, margins strongly involute, abaxially sparsely to densely antrorsely scabrous all over, adaxially scabrous over the veins and usually between them, sometimes densely so, narrowly prow-tipped; flag leaf blades 28–93(–190)% longer than their sheath, flag leaf blade 4–23 cm long; sterile shoot blades (3–)15–30 cm long, usually involute. **Panicles** (3–)7–30 cm long, erect to slightly nodding, contracted to eventually open, pyramidal, moderately congested, to spikelets (11–)70 to well over 100, proximal internode 1–5 cm long; rachis with (1–)2–5 branches per node; primary branches spreading initially or tardily, straight or slightly flexuous, angled, mostly densely scabrous mostly on the angles to all around; lateral pedicels averaging ca. 1/2 the spikelet in length, densely scabrous, prickles fairly coarse; longest branches (1–)2–10(–15) cm, with (2–)8–40 spikelets in distal ½. **Spikelets** 4–7(–7.5) mm long, lanceolate, laterally compressed, not bulbiferous, pale greenish to bluish; florets 2–5, pistillate or hermaphroditic; rachilla internodes terete, 0.8–1(–1.5) mm long, sparsely to densely scabrous, sometimes densely hirtellous; glumes narrowly lanceolate to lanceolate, slightly unequal, distinctly keeled, keels sparsely to densely scabrous for most of the length, lateral veins smooth to moderately scabrous, apical surfaces smooth or lightly scabrous, edges smooth or lightly scabrous, apex sharply acute; lower glumes 2–3.3 mm long, 1–3-veined; upper glumes 2.7–4.3 mm long, mostly 2 × as wide as the lower one, 3-veined; calluses glabrous (rarely dorsally webbed, web scant, with a few short hairs); lemmas 2.7–4(–4.6) mm long, short to long lanceolate, distinctly keeled, smooth or sparsely to densely scabrous, keels and marginal veins glabrous or softly puberulent to short villous, between veins glabrous or infrequently sparsely softly appressed puberulent near the base, intermediate veins moderately prominent to prominent, edges narrowly scarious-hyaline, smooth or with a few short hooks, apices obtuse to acuminate; paleas scabrous over the keels, prickles sub-erect, between keels asperous. **Flowers** chasmogamous; lodicules 0.4 mm long, ovate; anthers 2.2–2.5 mm long, often aborted late in development and 1–2 mm long (functionally pistillate). **Caryopses** 1.8–1.9 mm long, fusiform in side-view, slightly laterally compressed, light brown, sulcus narrow, very shallow, hilum 0.2 mm long, round to oval, grain adherent to the palea. 2*n* = 28–29, 29+II.

### Distribution.

The species occurs in the USA: Texas (Chisos Mountains) and Mexico: Chihuahua, Coahuila, Durango, Zacatecas.

### Ecology.

The species is found on steep protected slopes and bases of cliffs derived from igneous and calcareous substrates in the mountains, and the Chihuahuan Desert vegetation from upper creosote bush flats to middle elevations in pine-oak zones, between 1700–2800(–3195) m. The species is probably apomictic, in part, because many of the specimens have sterile, late-aborted anthers. Flowering March to July.

### Specimens examined.

Mexico. **Chihuahua:** 2–3 mi SW of Babicora, on south side of and above road to Madera, 29.2469°N, 107.7659°W, ca. 7000 ft [2135 m], 12 Apr 1984, R.J.Soreng 2304 & R.W.Spellenberg (NMC, US; 2*n* = 28+I, 2304a, 2*n* = 28−29+I , 2304b, Soreng 2005; Soreng 1990, cpDNA voucher). ca. 20 km SE of Chuatemoch, north side of La Bufa Mountain, Silver mine, 28.2443°N, 106.8039°W, 2050 m, 13 Apr 1984, R.J.Soreng 2308 & R.W.Spellenberg (NMC, US). **Coahuila:** Cañon de la Hacienda, Sierra de la Madera, northwest of Cuatro Cienega, conifer-oak forest above log-cutters camp, 8000−9000 ft [2440−2745 m], D.Pinkava P1364, McGill, Reeves, & Nash (ASU pistillate). Sierra de Parras, Apr 1905, C.A.Purpus 1146 (NY, GH , NY); ditto, Mar 1905, C.A.Purpus 1112 (GH, NY♂, NY). 9 km south of Parras on Sierras Negras, scrubby woodland association of pine, juniper, oaks, heavily grazed by goats, 2400 m., 3 Jul 1941, L.R.Stanford 167, K.L.Retherford & R.D.Northcraft (DS♀, ARIZ♀, GH♀, MEXU, MO, US). Mina el Aguirreno, north side of Sierra de la Paila, 1700–2200 m, chaparral, very steep slopes of limestone, calcareous gravel, 5 Jul 1973, M.C.Johnston 11706, T.L.Wendt & F.Chiang (MEXU, MO, LL-TEX♀). valley floor, 15 km west of Concepcion del Oro, just within Coahuila, 24°54'N, 101°45'W, 2300 m, 19 Jul 1941, L.R.Stanford 477, R.L.Retherford & R.D.Northcraft (ARIZ, GH, MO, NY, US, Marshall Johnston thinks this may be in Zacatecas, pers. comm. ca. 1986, the habitat is odd for this species or *Poa* in general, and we suspect it was collected on protected slopes near this vicinity). **Durango:** Sierra del Rosario, steep limestone sierra with some zones of igneous mineralization, 1800−2655 m, 25 Jun 1973, M.C.Johnston 11463, F.Chiang-C. & T.L.Wendt (CAS, MO, LL-TEX♀); ditto, 11464 (CAS, MEXU, MO, LL-TEX♀). **Zacatecas:** Sierra del Astillero, SE from Tanque el Alto, 2500−3195 m, limestone, 2 Jul 1973, M.C.Johnston 11566I , T.L.Wendt & F.Chiang-C. (LL-TEX♀); ditto, 11566J (MEXU, LL-TEX♀); ditto, 11566K (LL-TEX♀); ditto, 11566L (LL-TEX♀). Sierra El Astillero, ca. 1.8 mi SW of Santa Rosa and 5.4 mi SW of San Pedro at trail head above water pump, 24°38'5.8"N, 101°6'47.5"W, 2550 m, 21 Oct 2007, P.M.Peterson 21451, J.M.Saarela, & D.Stančik (US).

### Discussion.

This species is endemic to the mountains in and around the Chihuahuan Desert. It ranges from the Chisos Mts. of Texas, throughout the drier mountains of Coahuila, to the front ranges of the Sierra Madre Occidental, from northern Chihuahua south to northern Durango and Zacatecas. It is highly variable in stature, leaf form, ligule and spikelet length, but we have found no correlation among the morphological characteristics, geography, and/or substrate type. Ligule length is variable from 0.5 mm in some specimens to 2.5−7.5 mm in others. Spikelet length varies independently of ligule length and blade traits. Soreng (1991b, 524−525) placed *Poa involuta* and *Poa filiculmis* in synonymy of *Poa strictiramea* and discussed the variations. The majority of the collections are functionally pistillate (♀) with anthers aborted late in development, and only one was determined as staminate. The species is presumably apomictic in large part. It differs from *Poa palmeri* in having scabrous leaf blades that are more often involute, a web absent or of a few short hairs, and lemmas that are glabrous or have sparse or short lemma pubescence. Citations for Nuevo León (Soreng 1991b, Espejo Serna et al. 2000, Dávila Aranda et al. 2006) are presumably from misidentifications of *Poa palmeri* (R.L.McGregor 385 et al. at US). One specimen (*Gentry 2716* at MO) identified by P.C. Standley in Gentry (1942) as *Poa involuta* from Memelichi, Chihuahua, initially determined as *Poa tracyi*, is actually a *Trisetum filifolium* Scribn. ex Beal. with a very short dorsal awn. Herrera-Arrieta (Grasses of Chihuahua, in prep.) report the following additional specimens from Chihuahua: *R.Bye 8242 & W.A.Weber* (MEXU); Sierra Mojinora, *D.Stančik 6187 & S.Gonzalez* (MEXU), *G.Nesom s.n. & A.McDonald s.n*. (ARIZ), the latter two may prove to be *Poa matris-occidentalis*.

## 22. Poa thomasii

Refulio, Syst. Bot. 37(1): 130. 2012.

http://species-id.net/wiki/Poa_thomasii

Fig. 3 C, D

Stenochloa californica ≡ Nutt., Proc. Acad. Nat. Sci. Philadelphia 4: 25. 1848. *Dissanthelium californicum* (Nutt.) Benth., Hooker’ s Icon. Pl. 4: 56. 1881. (non *Poa californica* Steud., Syn. Pl. Glumac. 1: 261. 1854.) Type: USA, California, Santa Catalina Island, *Gambel s.n*. (holotype: GH!; isotype: US! fragm. ex GH & rough drawing, herbarium label for drawing has “Nuttall script", and header “Coll. NUTTALL, Presented by Elias Durand, 1866").

### Description.

Hermaphroditic. **Annuals**; tufted, tufts sparse, small, bases narrow, slender, bright green; tillers intravaginal (each subtended by a single elongated, 2-keeled, longitudinally split prophyll), without cataphyllous shoots, most shoots flowering. **Culms** (6–)10–46(–60) cm tall, erect or ascending, leafy, slender, leafy, terete, smooth; nodes 2–3, 2–3 exerted. **Leaf** sheaths slightly compressed, smooth, glabrous; butt sheaths thin papery, bases of butt sheaths glabrous; flag leaf sheaths up to 10 cm long, longer than those below it, margins fused ca. 50% their length, ca. equaling its blade; throats and collars smooth, glabrous; ligules 2–6 mm long, scarious-hyaline, abaxially smooth, glabrous, apex irregular, acute; blades 2.5–15(–20) cm long, (1–)2–4 cm wide, flat, thin, soft, abaxially smooth, margins lightly scabrous, adaxially smooth or slightly scabrous over costae, apex slender, not noticeably prow-tipped; flag leaf blades to 12 cm long. **Panicles** 5–16 cm long, erect, loosely contracted to open, slightly lax, moderately congested to sparse, with (10–)10–80 spikelets, proximal internode 2–4 cm long; rachis with 3–7 branches per node; primary branches sub-erect to ascending, slender, delicate, slightly angled, angles moderately scabrous; lateral pedicels to about 1/2 the spikelet length, moderately scabrous, prickles fine; longest branches 2–5 cm, with up to 12 spikelets some usually from near the base. **Spikelets** 2.5–5 mm long, cunniate at maturity, laterally compressed, not bulbiferous, green, sometimes anthocyanic, sub-lustrous; florets 2(–3), hermaphroditic; rachilla internodes terete, ca. 0.3 mm long, smooth, glabrous; glumes narrowly lanceolate, subchartaceous, green, lustrous, equal or subequal, both exceeding the florets, smooth, or keels scaberulous above, margins scarious, edges smooth, apex acuminate; lower glumes 3–4 mm long, 1–3-veined; upper glumes 3–4 mm long, 1–3-veined; calluses indistinct, glabrous; lemmas 1.5–2.2 mm long, 3(–5)-veined, ovate to elliptic, pale green, not lustrous, strongly keeled, keel smooth or sparsely scabrous above, surfaces minutely crisply appressed puberulent throughout the herbaceous portion, intermediate veins indistinct or absent, margins and apex narrowly scarious-hyaline, edges sometimes with a few hooks; apex obtuse to acute, sometimes denticulate in the upper margin; palea keels apically sparsely scabrous, medially glabrous or with a few hairs, surfaces glabrous or minutely pilose. **Flowers** mainly cleistogamous; lodicules 0.4 mm long, lanceolate, with a subapical lateral lobe; anthers 0.2(–0.4) mm long. **Caryopses** 1.1–1.2 mm long, elliptical in side-view, laterally compressed, sulcus indistinct, hilum ca. 0.15 mm long, elliptical. 2*n* = unknown.

### Distribution.

The species is found in the USA (California: Channel Islands) and Mexico (Baja California).

### Ecology.

This annual species responds to winter and spring rains and fog on the Pacific Coastal Islands of southern California and Baja California Sur. Flowering Mar through May.

### Conservation status.

*Poa thomasii* is listed as Federally Endangered in the United States, and it is rare and possibly extinct in Mexico.

### Specimens examined.

Mexico. **Baja California:** Isla Guadalupe, 1875, E.Palmer 96 (MO).

### Discussion.

From the time Bentham placed the species in *Dissanthelium* up to until Refulio-Rodríguez placed it in *Poa* (2012), it was known as *Dissanthelium californicum* (Hitchcock 1913, Beetle 1987, Espejo Serna et al. 2000, Dávila Aranda et al. 2006, Refulio-Rodríguez 2007). DNA data confirm that all species of *Dissanthelium* are nested within *Poa*, and collectively are not monophyletic (Refulio-Rodríguez et al. 2012). This species, which is morphologically and phylogenetically isolated from the core species of *Dissanthelium* placed in *Poa* sect. *Dissanthelium* (Trin.) Refulio, is endemic to the Channel Islands of southern California and Isla Guadalupe, Baja California. In Mexico it was collected on Isla Guadalupe by Dr. Edward Palmer in 1875 (Gould and Moran 1981, Beetle 1987). It was thought to be extinct in the United States until it was rediscovered, after grazing pressures from feral goats and pigs, etc., were reduced or removed, on Santa Catalina Island in California (McCune and Knapp 2008). Morphologically, it approaches *Poa howellii* Vasey & Scribn., a species of the adjacent lowlands in California (and north to British Columbia) that also reaches the Channel Islands.

## 23. Poa wendtii

Soreng & P.M. Peterson sp. nov.

urn:lsid:ipni.org:names:77121283-1

http://species-id.net/wiki/Poa_wendtii

Figs 22 23

### Type:

Mexico, Coahuila, [Sierra de Santa Rosa], Rincón de María, on Hacienda La Babia, ca. 70 road mi NW from Múzquiz, 28°27'30"N, 102°05'W, 1750 m, Steep NE facing talus and stabilized areas below high cliffs in southwest part of rincón, southwest of “Slump Spring", woods of *Abies coahuilensis*, *Yucca* cf. *thompsoniana* Trel., *Quercus* *gravesii* Sudw., *Quercus* sp., *Tilea*, *Cercis*, *Juglans*, *Agave* sp., 27 Apr 1975, *T.L.Wendt 883 & D.Riskind* (holotype: LL-TEX-75341!).

### Diagnosis.

*Poa wendtii* differs from other open-panicled, broad-leaved, long-anthered species of *Poa* from around the region (e.g. *Poa matris-occidentalis*, *Poa palmeri*, and *Poa tracyi*) by having abruptly reduced upper culm leaf blades and more compact, ovate (non lanceolate) spikelets in lateral view.

### Description.

Hermaphroditic (apparently). **Perennials**; tufted, sub-rhizomatous, tufts loose, moderate girth and height, bluish-grey-green; tillers extravaginal (basally cataphyllous), with lateral or downward tending, cataphyllous shoots, cataphylls smooth, sub-lustrous, brownish or anthocyanic. **Culms** 50–70 cm tall, erect, blades reduced upward, terete, smooth; nodes 3–4, 0 or uppermost node exposed. **Leaf** sheaths slightly compressed, proximal culm ones smooth, glabrous, middle ones densely finely scabrous, upper ones moderately to lightly scabrous; butt sheaths cataphyllous, smooth, glabrous; flag leaf sheaths 13–16 cm long, margins fused for 30–42% their length, 14–16 cm long, 10–20 × longer than its blade; collar margins of lower and mid culm leaves slightly asperous to coarsely short ciliate; ligules 1.2–1.8 (top leaf), 2.7–3.3(2nd leaf down), to 3 mm on the longest leaves, whitish, abaxially sparsely to moderately scabrous, margins smooth, apices obtuse to acute, slightly irregular; blades to 22 cm long, mostly 3–5 mm wide, blades folded or flat when fresh, distinctly keeled, moderately thick, retaining form on drying, glaucous (all surfaces waxy coated), margins abruptly slightly inrolled, abaxially densely short scabrous, with short broad-based closely appressed hooklets, ribs slightly expressed, margins somewhat thickened, and finely scabrous, adaxially smooth to sparsely short scabrous, with very shallow ridges and furrows, broader leaves with ca. 15 ribs, prominently prow-tipped; lower mid-cauline blades the longest, each succeeding that, shorter than the one below by ca. 1/2, that of the flag leaf blades reduced, 0.7–1.3 cm long; sterile shoot blades similar to cauline blades in form. **Panicles** 13–14 cm long, erect to slightly nodding, open, broadly pyramidal, slightly secund, fairly sparse, well exerted, with 50–70 spikelets, peduncles smooth, proximal internode 2.5–4 cm long, smooth or lightly scabrous; rachis with 2–3 branches per node; primary branches widely spreading to reflexed, slender, slightly flexuous, proximally terete or slightly angled, distally moderately angled, proximally smooth, distally moderately scabrous mostly on the angles; lateral pedicels about half as, to as long as, their spikelets, moderately scabrous; longest branches 5–7 cm, longest with 8–11 spikelets in the distal 1/2. **Spikelets** 4–6 mm long, 2.5–3 × as long as wide, broadly lanceolate, laterally compressed, not bulbiferous, purplish throughout at maturity; florets (2–)3(–4), hermaphroditic; rachilla internodes terete, 0.5–1 mm long, mostly hidden, terete, muriculate, glabrous; glumes lanceolate, sub-lustrous, slightly unequal, both shorter than or the 2nd equaling the first lemma, distinctly keeled, keels sparely to moderately short scabrous distally, surfaces with smooth, with scarce short cells, or with some hooks near the apex, edges smooth, apices acute, lower glumes 2.5–3.2 mm long, 1–3-veined; upper glumes 3.3–3.7 mm long, 3-veined; calluses dorsally webbed, web distinct, hairs to ca. 1–2 mm long, woolly; lemmas 3.5–4 mm long, 5-veined, elliptical in side view, anthocyanic at maturity, distinctly keeled, keel and marginal and intermediate veins villous for 2/3, 1/3, and sometimes 1/4 their length, respectively, between veins sparsely to moderately lanate in proximal half, intermediate veins distinct, upper margins narrowly to broadly bronzy-scarious to hyaline, edges smooth or sparingly scabrous, apices obtuse to broadly acute, palea keels scabrous in the distal half, sometimes with a several soft hairs medially, intercostal region sulcate, densely muriculate, usually sparsely puberulent in the proximal third. **Flowers** chasmogamous; anthers 1.6–2.1 mm long. **Caryopses** 1.7–2.0 mm long, elliptical in side-view, compressed, laterally compressed, brown, distinctly shallow sulcate, hilum 0.25 mm long, oval, grain adherent to the palea. 2n = unknown.

### Distribution.

The new species is known only from the type collection from the Sierra de Santa Rosa, Coahuila.

### Ecology.

The species occurs on sheltered talus and cliff bases, in forests of *Abies coahuilensis* I.M. Johnst. at 1750 m. The only specimen known has perfect flowers. Flowering in Apr to May.

### Etymology.

It is our pleasure to name this new species in honor of Thomas Leighton Wendt (born 1950) who collected this and many other plants (including *Poa*) in the Chihuahuan Desert region.

### Conservation status.

The species is rare.

### Discussion.

The type collection is presumed to be a unicate (T.L.Wendt, pers. comm. 2011). The species which appears to be endemic to the Sierra de Santa Rosa, should be considered extremely rare and possibly endangered.

**Figure 22.**
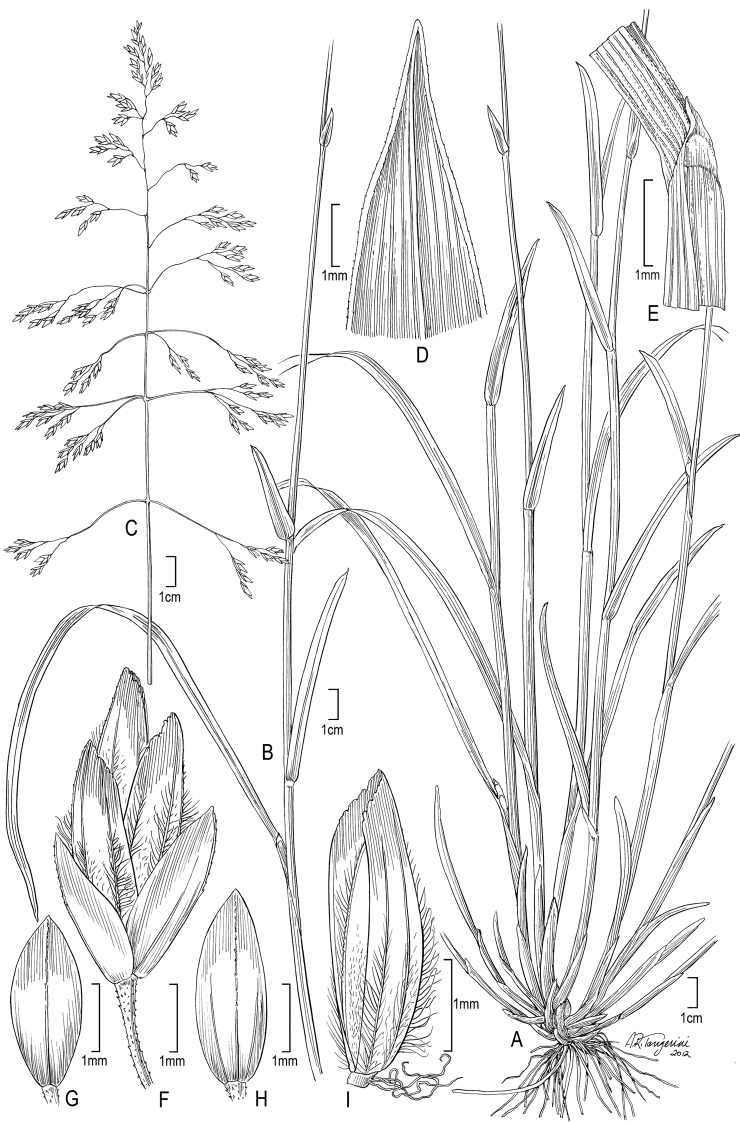
*Poa wendtii* Soreng & P.M. Peterson **A** habit **B** culm **C** inflorescence **D** blade apex **E** ligule **F** spikelet **G** lower glume **H** upper glume **I** floret. Drawn from the holotype collection (*Wendt 883 & Riskind*).

**Figure 23.**
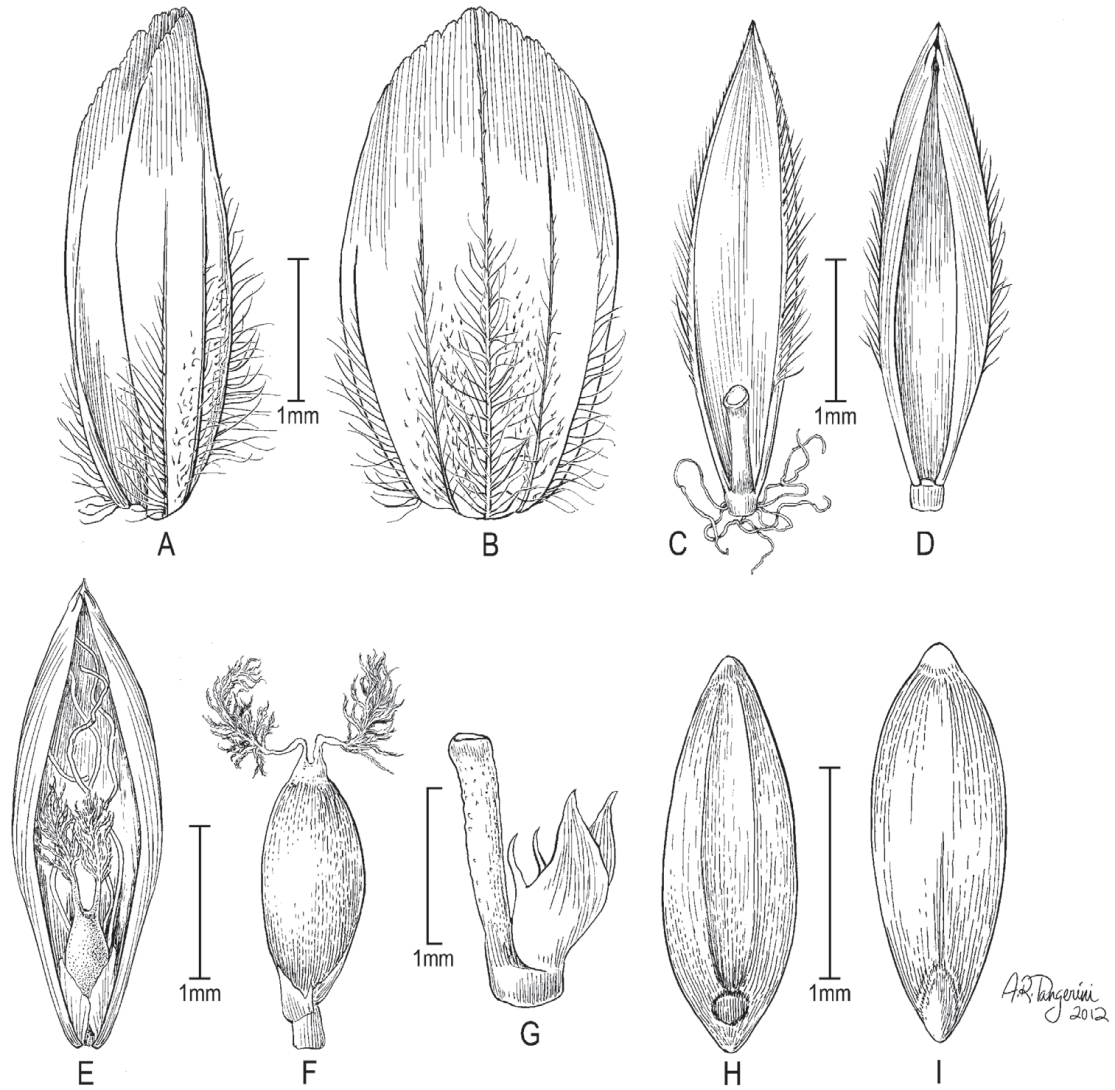
*Poa wendtii* Soreng & P.M. Peterson **A** lemma **B** lemma abaxial view **C** palea dorsal view **D **palea ventral view **E** perfect flower enclosed in palea with lodicules, pistil, and stamen filaments **F** perfect flower with pistil/partly developed caryopsis **G** lodicules and rachilla internode **H** caryopsis ventral view, with sulcus and hilum **I** caryopsis dorsal view.Drawn from the holotype collection (*Wendt 883 & Riskind*).

## Species excluded

### Poa acinaciphylla

E. Desv. (syn. Poa villaroelii Phil.)

#### Discussion.

This taxon is a narrow endemic of the central Andes of Chile and Argentina (Giussani et al. 2012). Reports from Mexico by Hitchcock to Dávila Aranda, from 1913 to 2006, as *Poa villaroellii*, and Clayton et al. (2006) as *Poa acinaciphylla* are based on the specimens here referred to *Poa chamaeclinos*.

### Poa nemoralis

L.,Sp. Pl. 1: 67 1753.

#### Discussion.

This species was reported by Dávila Aranda et al. (2006) from Coahuila, Mexico, and has not been verified by us.The basis of this report is unknown to Jesus Valdes-Reyna (pers. comm. 2012, a coauthor of the Dávila Aranda et al. 2006 account, who is working on a revision of grasses of Coahuila). We guess that this reference resulted from confusion with the name *Agrostis alba* L., the type of which is *Poa nemoralis*. The accepted name for material traditionally treated as *Agrostis alba* (sensu auct.) is *Agrostis gigantea* Roth. This is clearly the case for a separate report for Durango: See *A.Benitez 1725* (CIIDIR) in GBIF (http://data.gbif.org/occurrences/370590415/), mapped for Durango as *Poa nemoralis*, but cited under *Agrostis alba* L. by Herrera-Arrieta (2001). *Poa nemoralis*, is cool-temperate woodland species of *Poa* sect. *Stenopoa* Dumort., native to Eurasia and introduced in Canada, USA (mostly in the northeast), sporadically in Patagonian South America, and in Guatemala (*M.E.deKoninck s.n*., ISC-212372!).

## Infrageneric classification of the species of *Poa* in Mexico.

(*taxonomic placement supported by DNA data; vouchers from Mexico cited above)

*Poa* subg. *Ochlopoa* (Asch. & Graebn.) Soreng & L.Gillespie sect. *Alpinae* (Hegetschw. ex Nyman) Stapf: *Poa alpina; sect. *Micrantherae* Stapf: *Poa annua, *Poa infirma.

*Poa* subg. *Poa* supersect. *Homalopoa* (Dumort.) Soreng & L.Gillespie sect. *Dissanthelium* (Trin.) Refulio: *Poa calycina; sect. *Homalopoa* Dumort. s.l.: *Poa bajaensis, *Poa bigelovii, *Poa chamaeclinos, *Poa gymnantha, *Poa matris-occidentalis, *Poa occidentalis, *Poa orizabensis, *Poa palmeri, *Poa ruprechtii*, *Poa scaberula [sometimes separated in *Poa* sect. *Dasypoa* (Pilg.) Soreng], *Poa seleri*, *Poa strictiramea, *Poa thomasii, *Poa wendtii*; subsect.* Papillopoa*: *Poa mulleri; sect. *Madropoa* Soreng subsect. *Madropoa* Soreng: *Poa fendleriana.

*Poa* subg. *Poa* supersect. *Poa* sect. *Poa*: *Poa pratensis.

*Poa* subg. *Stenopoa* sect. *Tichopoa* Asch. & Graebn.: *Poa compressa.

*Incertae sedis*: *Poa* sect. *Secundae* V.L. Marsh ex Soreng subsect. *Secundae* Soreng (subg. *Poa* × subg. *Stenopoa* sect. *Stenopoa*): *Poa secunda.

## Supplementary Material

XML Treatment for Poa

XML Treatment for Poa alpina alpina

XML Treatment for Poa annua

XML Treatment for Poa bajaensis

XML Treatment for Poa bigelovii

XML Treatment for Poa calycina mathewsii

XML Treatment for Poa chamaeclinos

XML Treatment for Poa compressa

XML Treatment for Poa fendleriana

XML Treatment for Poa fendleriana albescens

XML Treatment for Poa fendleriana fendleriana

XML Treatment for Poa fendleriana longiligula

XML Treatment for Poa gymnantha

XML Treatment for Poa infirma

XML Treatment for Poa matris-occidentalis

XML Treatment for Poa matris-occidentalis matris-occidentalis

XML Treatment for Poa matris-occidentalis mohinorensis

XML Treatment for Poa mulleri

XML Treatment for Poa Papillopoa

XML Treatment for Poa occidentalis

XML Treatment for Poa orizabensis

XML Treatment for Poa palmeri

XML Treatment for Poa pratensis

XML Treatment for Poa pratensis agassizensis

XML Treatment for Poa pratensis alpigena

XML Treatment for Poa pratensis angustifolia

XML Treatment for Poa pratensis irrigata

XML Treatment for Poa pratensis pratensis

XML Treatment for Poa ruprechtii

XML Treatment for Poa scaberula

XML Treatment for Poa secunda secunda

XML Treatment for Poa seleri

XML Treatment for Poa strictiramea

XML Treatment for Poa thomasii

XML Treatment for Poa wendtii

XML Treatment for Poa acinaciphylla

XML Treatment for Poa nemoralis
